# Genomic dissection of iron toxicity tolerance in rice identifies key loci, candidate genes, and associated haplotypes

**DOI:** 10.1038/s41598-026-38841-9

**Published:** 2026-03-09

**Authors:** Sandeep Jaiswal, Kuldeep Kumar, Anita Kumari, Binay K. Singh, Alka Bharati, Pankaj Baiswar, Ayam Gangarani Devi, Krishnappa Rangappa, Manjeet Talukdar, Philanim Shimray, Letngam Touthang, Samarendra Hazarika

**Affiliations:** 1 ICAR Research Complex for NEH Region, Umiam, 793 103 Meghalaya India; 2 ICAR-National Institute for Plant Biotechnology, Pusa Campus, New Delhi, 110 012 India; 3 Department of Plant and Soil Science, Texas Tech University, Lubbock, TX 79409 USA; 4 ICAR-Indian Institute of Wheat and Barley Research, Karnal, 132 001 Haryana India; 5 ICAR Research Complex for NEH Region, Regional Station, Tripura, 799 210 India

**Keywords:** Soil acidity, Iron toxicity tolerance, Rice, meta-QTL analysis, Marker–trait associations, Computational biology and bioinformatics, Genetics, Molecular biology, Plant sciences

## Abstract

Rice (*Oryza sativa* L.) is known for its inherent tolerance to highly acidic soils, yet the underlying genetic and physiological mechanisms, particularly for iron (Fe) toxicity tolerance, remain insufficiently characterised. Although several quantitative trait loci (QTLs) and candidate genes (CGs) associated with Fe-toxicity tolerance have been identified, their fine mapping is unresolved. In this study, we performed a meta-QTL (M-QTL) analysis by integrating data from 11 QTL mapping studies and nine genome-wide association studies (GWAS), resulting in 63 M-QTLs with a minimum cluster size of two. The largest cluster comprised 10 QTLs, with an average cluster size of 4.01. Phenotypic variance explained (R^2^) ranged from 7% to 31% (mean 11.77%), and the average confidence interval (CI) was reduced from 4.68 to 2.12. Gene retrieval within these M-QTL regions identified 4,070 non-redundant genes. Comparison with 5 Fe-toxicity-related transcriptomic datasets revealed 897 differentially expressed genes, of which 284 were common to at least two datasets and designated as CGs. Characterisation of these CGs identified key regulatory elements, transcription factors, and transporters implicated in Fe tolerance. Notable CGs included *OsNRAMP6*, *OsCDAP1*, *OsFRO2*, *OsFRDL2*, *OsPT2*, *OsPDR9*, *OsHSP70*, *OsACS2*, *OsZIP8*, *OsWRKY46*, *OsTIP2;2*, *OsGSTU17*, *OsINH2*, and *OsPEZ1*. Association analysis identified 13 novel marker–trait associations (MTAs), and haplotype analysis revealed nine haplotypes (H001–H009). M-QTL1.6 and M-QTL7.3 were characterised by large QTL clusters and multiple candidate genes, while M-QTL10.1 contained an Fe-tolerance–associated haplotype within *OsFRDL2*. Key MTAs (MTA8, MTA16, MTA19, MTA24) formed central nodes in protein–protein interaction networks. This study identifies stable M-QTLs and favourable haplotypes associated with Fe toxicity tolerance in rice, offering clear genomic targets that regulate plant growth under excess iron stress.

**Supplementary Information:**

The online version contains supplementary material available at 10.1038/s41598-026-38841-9.

## Introduction

Rice (*Oryza sativa* L.) is the primary staple food crop, supplying approximately 21% of global caloric intake and meeting up to 76% of dietary requirements in Southeast Asia^1^. However, its productivity is significantly constrained by acidic soils, which account for nearly 30–40% of the world’s arable land^2–4^. Acidic soils induce severe physiological and biochemical stresses on plants, primarily through deficiencies in essential macronutrients such as phosphorus (P), calcium (Ca), and magnesium (Mg), as well as micronutrients including molybdenum (Mo) and boron (B). Furthermore, these soils facilitate the toxic accumulation of aluminium (Al) and iron (Fe) where Fe toxicity represents a major yield-limiting factor, with yield losses ranging from 12% to complete crop failure^5^.

Iron toxicity in rice is typically manifested through leaf bronzing, stunted shoot and root growth, and inhibition of primary root elongation, which consequently limits lateral root development^6,7^. This inhibition primarily results from the suppression of both cell elongation and division at the root apex^8–10^. Although the mechanisms underlying Fe toxicity are yet to be completely understood, elucidating them is critical for developing strategies to mitigate its adverse effects, especially under anaerobic or waterlogged conditions. In such environments, ferric iron (Fe^3+^) is reduced to the more soluble ferrous form (Fe^2^⁺), which is readily absorbed by roots. Excessive Fe^2^⁺ uptake leads to cytotoxicity and disrupts key metabolic and physiological processes, including carbon metabolism, respiration, enzyme activity, membrane integrity, and water homeostasis^11–14^.

Within chloroplasts, excess Fe^2^⁺ catalyzes the Fenton reaction, generating highly reactive hydroxyl radicals (·OH) through interactions with hydrogen peroxide (H_2_O_2_). These radicals cause oxidative damage to lipids, proteins, and nucleic acids, thereby impairing vital cellular functions^15–19^. The overproduction of reactive oxygen species (ROS) through this process further disrupts chloroplast function, electron transport, and cellular metabolism, leading to compromised plant health.

Plants maintain Fe homeostasis through tightly regulated uptake, transport, storage, and sequestration mechanisms to prevent toxic Fe^2^⁺ accumulation and secondary ROS generation^13,14,20^. In roots, tolerance mechanisms to Fe toxicity include the formation of Fe^3+^-rich plaques that limit further Fe uptake, facilitated by root aerenchyma development, lateral root formation, and enzymatic oxidation of Fe^2^⁺^17,21,22^. Iron may also be immobilised in “dumping sites” within roots, forming inactive complexes to mitigate its toxic effects^17^. In shoots, excess Fe is compartmentalised within low-photosynthetic tissues such as the leaf sheath and stem^23^ and sequestered in vacuoles^24^. Ferritin, a crucial Fe-storage protein, can store up to 4,000 Fe atoms within plastids and plays a key role in Fe detoxification and tolerance^25^. Additional shoot-based tolerance mechanisms involve the activation of antioxidant systems that scavenge ROS^26,27^.

Identifying stable genomic regions associated with Fe toxicity tolerance is critical for developing rice varieties that can thrive in Fe-contaminated soils. Stable genetic markers facilitate precise breeding for the target trait, supporting the development of climate-resilient varieties^28^. Numerous studies have reported quantitative trait loci (QTLs) and candidate genes (CGs) associated with Fe toxicity tolerance in rice using both intra- and interspecific mapping populations^10,29–31^. Additionally, genome-wide association studies (GWAS) have identified several genomic regions linked to Fe toxicity tolerance^10,32,33^. However, inconsistencies arising from variations in experimental design, environmental conditions, genetic backgrounds, and statistical methodologies have led to QTLs with limited reproducibility and phenotypic variance. Fine mapping of causal genes for most QTLs is incomplete, limiting their effective use in rice breeding programs.

Meta-QTL (M-QTL) analysis offers a powerful strategy to refine confidence intervals (CIs) of overlapping QTLs, enabling more precise identification of key loci and genetic markers associated with complex agronomic traits^34,35^. This approach has been widely applied in rice to elucidate genomic regions governing grain weight^36^, drought tolerance^37^, salinity tolerance^38^, blast resistance^39^, P-use efficiency^40^, and Al toxicity tolerance^41^. However, despite these advances, integrative M-QTL-based frameworks specifically addressing Fe toxicity tolerance—particularly those combining M-QTL refinement with CG prioritisation, haplotype analysis, and network-level functional interpretation—remain limited. Building on these advances, the present study identifies stable M-QTLs associated with Fe toxicity tolerance in rice and prioritises CGs within these regions through integrated transcriptomic, cis-regulatory, and network-based analyses. CG–based association and haplotype analyses were further employed to highlight allelic variants linked to Fe toxicity response. By integrating large-scale QTL/GWAS evidence with transcriptome-guided prioritisation and haplotype analysis, this work provides a systems-level synthesis of the genetic architecture underlying Fe stress adaptation in rice. The resulting M-QTLs, CGs, and haplotypes represent biologically informed, hypothesis-driven targets emerging from cross-study convergence, offering a structured framework for downstream functional validation and breeding-oriented exploration.

## Materials and methods

### Literature survey, QTL data collection, and input file Preparation

A comprehensive literature survey was conducted, covering research articles published up to December 2024 and a total of 20 mapping studies were selected for meta-analysis, comprising 11 biparental mapping studies and nine GWAS (Table 1). The compiled QTL dataset included essential parameters such as QTL name, associated trait, chromosomal location, logarithm of odds (LOD) value, phenotypic variance explained (R^2^), QTL position, and CI. For studies in which LOD scores and phenotypic R^2^ were not reported, default values of LOD = 3.0 and R^2^ = 10% were assigned to enable QTL harmonisation in meta-analysis, following established practice in previous M-QTL studies^40,42,43^. These values were used to facilitate consensus mapping across heterogeneous datasets rather than as precise estimates of QTL effect size. For GWAS-derived loci, marker physical positions (base pairs) were converted to genetic distances (centiMorgans, cM) using an average conversion factor of 200 kb cM^−1^, consistent with approaches used to integrate physical and genetic maps in rice M-QTL analyses^44^. Sensitivity analysis was performed on chromosome 7 by contrasting the baseline scenario (S0; with default assumptions) against 10 alternative scenarios (S1–S10) that independently and jointly varied default LOD thresholds (2.5–3.5), assumed R^2^ values (5–15%), and physical-to-genetic distance conversion ratios (150–250 kb cM^−1^) (Table S1). Robustness of M-QTL localization was evaluated by comparing M-QTL peak positions across scenarios using a symmetric mean nearest-neighbor distance metric, supported by heatmap visualization and hierarchical clustering.

**Table 1:** Summary of the Fe toxicity tolerance-related QTL mapping studies in rice employed for meta-analysis.

S. no.	Mapping population			Marker		QTL(s) identified		References
	Cross	Type	Size	Type	Number	Trait	Number	
1	IR64 × Azucena	DH/BC1F1	123/100	RFLP	175	RDSDW	5	Wu et al., 1997^74^
2	IR64 × Azucena	DH	135	RFLP	175	LBI, Fe conc., APA, GRA, DHA, DHA/AS	11	Wu et al., 1998^75^
3	Nipponbare/Kasalath//Nipponbare	BC_1_F_9_	96	RFLP	245	LBI, SDW, TN and RDW	8	Wan et al., 2003^76^
4	Azucena × IR64	RILs	164	SSR	228	LBI, SWC, SDW, RDW, RVSDW, RVRWD, SIC, SR, CCI	24	Dufey et al., 2009^5^
5	Gimbozu × Kasalath	F_3_	78	SSR/STS	81/16	QBS	12	Shimizu et al., 2009^28^
6	Azucena × IR64	RIL	164	SSR	228	LBI, SWC, SDW, SIC, SR, CCI, TPB, PDW, NSP, FR, GCL, 100GW	73	Dufey et al., 2012^67^
7	IR29 × Pokkali	RIL	121	SNP, SSR	173, 83	LBI	7	Wu et al., 2014^21^
8	Caiapo/MG12//Caiapo	BC_3_DH	220	SSR	126	LBI, SWC, SDW, RDW, CCI, SC, Fv/Fm, NPQ, BFe, SFe, RFe	46	Dufey et al., 2015^29^
9	*japonica* variety 02,428 × Minghui 63	RIL(BC_2_F_8_)	198 + 226	SNP	265	RRDW	9	Liu et al., 2016^64^
10	N/A	MAGIC	873	SNP	55k	SDW, RDW, RSL, RRL, RSDW, RRDW	21	Meng et al., 2017^68^
11	Neda × Ahlemitarom	Inbred Line	96 78	SSR, ISSR, IRAP, iPBS	59	FB, RL, SL, RN, LW, RFW, RDW, FC	22	Sabouri et al., 2021^117^
12	N/A	Rice accession	329	SNP	44,100	LBS	54	Matthus et al., 2015^32^
13	N/A	BL & LG	242 (192 + 50)	SNP	384	LBS	13	Utami et al., 2020^118^
14	N/A	Germplasm lines	119	SSR	51	LBI	23	Pawar et al., 2021^119^
15	Nipponbare/Kasalath//Nipponbare	BIL(BC_1_F_5_)	98	SSR	173, 83	LBS	3	Wu et al., 2014^21^
16	N/A	*Indica* rice accessions	222	SNP	395,553	SH, RL, SFW, SDW, RDW, SWC, SFe	29	Zhang et al., 2017^9^
17.	N/A	Thai rice accessions	270	SNP	3k	RFW, RSDW, RRFW, SL, RDW, SFW, RL, RRL,		Kaewcheenchai et al., 2021^32^
18.	N/A	BAAP	226	SNP	2,053,863	AWD, CF	46	Talukdar et al., 2021^120^
19.	N/A	Thai accession	239	SNP	160,498	SH, SFW, SDW, RFW, RDW	34	Theerawitaya et al., 2022^92^
20.	N/A	3k accession	551	SNP	2,955,964	SH_Fe, RL_Fe, SFW_Fe, RSH, RRL, and RSFW	29	Miao et al., 2024^60^

Relative Root Elongation (RRE), Relative Root Length (RRL), Root Tolerance Index (RTI), Relative Shoot Length (RSL), Relative Root Weight (RRW), Relative Shoot Weight (RSW), Shoot Height (SH), Root Length (RL), Shoot Dry Weight (SDW), Root Dry Weight (RDW), Control Root Elongation (CRE), Control Shoot Elongation (CSE), Relative Root Elongation (RRE), Control Root Length (CRL), Control Shoot Length (CSL), Stress Root Length (SRL), Stress Shoot Length (SSL), Shoot Water Content (SWC), Relative Root Fresh Weight (RRFW), and Relative Root Dry Weight (RRDW). Relative Decrease in Shoot Dry Weight (RDSDW), Leaf Bronzing Index (LBI), Leaf Bronzing Score (LBS), Shoot Water Content (SWC), Shoot Dry Weight (SDW), Shoot Fron Concentration (SIC), Stomatal Resistance (SR), Chlorophyll Content Index (CCI), Total Plot Biomass (TPB), Mean Panicle Dry Weight (PDW), Number of Spikelets Per Panicle (NSP), Fertility Rate (FR), Growth Cycle Length (GCL), and 100-Grain Weight (100GW).

To ensure consistency, single-nucleotide polymorphism (SNP) markers were integrated by aligning their physical positions with the closest markers on the reference rice linkage map established by Tenmykh et al. (2001)^45^. Furthermore, to incorporate QTLs from GWAS data, a symmetrical range of ± 2 cM was applied to define the CI for potential QTL positions, thereby increasing the likelihood of capturing CG located within regions in linkage disequilibrium (LD) with marker–trait associations (MTAs). Marker positions within each linkage group from individual studies were used to construct separate genetic map files, which were subsequently utilised for the meta-analysis.

### Consensus map construction and QTL meta-analysis

A consensus marker map was constructed by integrating genetic map data from 20 studies with the reference rice linkage map developed by Temnykh et al. (2001)^45^, as summarized in Table 1. This integration was carried out using BioMercator v4.2.3^46,47^, which enabled the alignment and merging of marker data to generate a comprehensive consensus map representing the combined marker positions across all studies. For the QTL meta-analysis, diverse traits related to Fe toxicity responses (Table 1) including visual symptoms, growth and biomass traits, physiological parameters, and Fe accumulation indices were consolidated under a single composite category designated as ‘FeTol’. This integrative classification was adopted to enable consensus mapping across heterogeneous studies, recognising that these traits represent interconnected manifestations of Fe^2^⁺ toxicity rather than a single uniform phenotype. The two-step algorithm proposed by Veyrieras et al. (2007)^48^ was employed, wherein the first step determined the optimal number of QTL models based on the lowest Akaike Information Criterion (AIC) value. In the second step, M-QTL regions associated with Fe toxicity tolerance were projected onto specific chromosomes, allowing the estimation of the number and positions of M-QTLs governing Fe toxicity tolerance in rice.

### Transcriptome survey to identify CGs within M-QTL regions

To identify CGs within the M-QTL regions, the physical coordinates of markers flanking the projected M-QTLs were retrieved from the Gramene Marker Database (http://archive.gramene.org/qtl/)^49^. For markers lacking physical position information, the coordinates of adjacent genetic markers were used to define the start and end points of the corresponding QTL region. Genes located within these marker intervals were batch downloaded from the Rice Annotation Project Database (RAP)^50^. The retrieved genes were then cross-referenced with expression datasets from multiple RNA-seq studies related to Fe toxicity tolerance in rice^10,51–54^. Genes were designated as CGs if they exhibited consistent differential expression in at least two independent RNA-seq datasets, a conservative filter intended to prioritise reproducible responses while recognising that context- or genotype-specific regulators may not be captured. To further elucidate the potential functional roles of these CGs, gene ontology (GO) analysis was performed using ShinyGO v0.77^55^.

### Promoter analysis, compartmentalisation, and identification of transcription factors (TFs) among the CGs

We performed an in-silico analysis of the promoter regions of selected key CGs to gain deeper understanding into the regulatory mechanisms underlying Fe toxicity tolerance in rice. Nucleotide sequences extending 2 kb upstream of the transcription start site (ATG) were retrieved from the Rice Annotation Project Database (RAP) (https://rapdb.dna.affrc.go.jp). Molecular signatures, motifs, and regulatory elements within these promoter sequences were analysed using the PlantCare database^56^. Potential TF-coding genes among the selected CGs were identified using the PlantTFDB v5.0 web server (http://planttfdb.gao-lab.org/prediction.php). Additionally, the subcellular localisation of the CGs was predicted using DeepLoc v2.1^57^, and the presence of transmembrane domains was assessed using DeepTMHMM v1.0^58^ to determine whether any CGs may function as transporters.

### Protein–Protein interaction (PPI) network analysis of Fe-M-QTL CGs

To elucidate the PPI network associated with Fe toxicity tolerance, a total of 284 CGs located within Fe-related M-QTL regions were analysed. The corresponding protein sequences were retrieved from the Rice Genome Annotation Project (RGAP) and submitted to the STRING database (v11.5; https://string-db.org/), using *Oryza sativa* as the reference organism. The resulting interaction network was clustered using the Markov Cluster Algorithm (MCL) with an inflation parameter of five to identify co-regulated or functionally related protein modules. This clustering approach facilitated the delineation of potential functional modules among the CGs.

### Candidate gene-based association analysis for Fe toxicity tolerance and haplotype analysis of identified MTAs

We further conducted a CG-based association study using the shortlisted CGs^59,60^. For this analysis, we utilised published phenotypic and genotypic data for 551 rice accessions from the 3k-RG panel^61^. Six Fe-toxicity tolerance traits, including shoot height (SH_Fe), root length (RL_Fe), shoot fresh weight (SFW_Fe), and their corresponding relative indices (RSH, RRL, and RSFW), were analysed^61^. Genotypic data for these 551 accessions were retrieved from SNP-Seek (https://snp-seek.irri.org/)^62^ and filtered using bcftools v1.17^63^ to generate a multi-VCF file. SNPs with > 15% missing data or minor allele frequency (MAF) ≤ 0.05 were removed, as were multi-allelic SNPs and multi-nucleotide polymorphisms (MNPs). The resulting VCF was further filtered to include only SNPs from CGs located within the M-QTL regions. Association analysis was performed using GAPIT v3^64^, implementing two models: the Fixed and Random Model Circulating Probability Unification (FarmCPU)^65^ and the Bayesian Information and Linkage Disequilibrium Iteratively Nested Keyway (BLINK)^66^. MTAs were filtered for robustness, retaining only those detected by both models or associated with more than one trait. Consequently, 18 genic SNPs identified as significant MTAs were selected for haplotype construction. Haplotype analysis was conducted using the R package geneHapR^67^. Initially, all 551 genotypes were included; however, to minimise artefacts arising from missing data and haplotype fragmentation, only SNPs and genotypes with 0% missingness were retained, restricting the analysis to homozygous haplotypes and improving internal statistical consistency. Subsequently, haplo-phenotype analysis was performed using geneHapR for three traits under Fe treatment conditions, including SH_Fe, SFW_Fe, and RL_Fe, to evaluate phenotypic variation among the haplotypes.

## Results

### QTLs for Fe toxicity tolerance in rice

Numerous studies have investigated QTLs associated with Fe toxicity–related traits in rice, utilising a wide range of phenotypic traits (Table 1). Among these, the leaf bronzing index (LBI) has been the most commonly assessed trait. Growth- and biomass-related traits, including shoot length (SL), root length (RL), shoot dry weight (SDW), root dry weight (RDW), shoot water content (SWC), total plot biomass (TPB), mean panicle dry weight (PDW), number of spikelets per panicle (NSP), fertility rate (FR), growth cycle length (GCL), and 100-grain weight (100GW), have also been extensively evaluated. Several stress-response indices have been employed, such as metal tolerance score (MTS), relative decrease in shoot dry weight (RDSDW), relative root dry weight (RRDW), SDW ratio, SPAD ratio, and bronzing score (BS). Additionally, physiological and biochemical traits under Fe^2^⁺ stress, including ascorbate peroxidase activity (APA), glutathione reductase activity (GRA), dehydroascorbate concentration (DHA), DHA/ascorbate ratio (DHA/AS), photosystem II maximum quantum efficiency (Fv: Fm), stomatal resistance (SR), and chlorophyll content index (CCI), have been explored. Furthermore, iron accumulation traits, such as shoot iron concentration (SIC), shoot Fe content, and grain Fe content, have been analysed to elucidate internal Fe dynamics.

We conducted a comprehensive literature survey and compiled data for 474 QTLs/QTNs (220 from biparental mapping and 254 from GWAS studies) associated with the aforementioned traits from 20 distinct studies (Table 1). These QTLs/MTAs were identified in biparental, multiparental, and natural populations, with population sizes ranging from 78 to 424 progenies for biparental mapping studies and 222 to 2,174 accessions for association mapping studies. Various molecular markers, including simple sequence repeats (SSRs), single-nucleotide polymorphisms (SNPs), restriction fragment length polymorphisms (RFLPs), and amplified fragment length polymorphisms (AFLPs), were employed across these studies (Table 1). The number of QTLs detected in biparental populations varied widely, ranging from 3^22^ to 73^68^. Among the chromosomes, chromosome 1 harboured the highest number of QTLs (77), whereas chromosome 12 had the fewest (5). CIs of these QTLs ranged from 0.05 to 40.7 cM, with phenotypic variance explained (R^2^) ranging from 2.5% to 47.9%^29^. Similarly, the number of high-confidence SNPs detected through GWAS varied from 5^33^ to 54^32^. Chromosome 3 contained the highest number of associations (29), whereas chromosome 8 had the lowest (7). The R^2^ values for these SNPs ranged from 1.8% to 18.9%^69^.

### Identification of M-QTL regions for Fe toxicity tolerance

We first constructed a consensus map by integrating marker information from the 20 studies onto the reference linkage map of rice^45^. The resulting consensus map comprised 20,001 markers, spanning a total genetic length of 1,596.12 cM, with chromosome 9 being the shortest (90.06 cM) and chromosome 1 the longest (172.76 cM). Of the 474 initially retrieved QTLs, 354 (74.68%) were successfully projected onto the consensus map. Meta-analysis of these projected QTLs identified 85 M-QTLs (Table S2) using the Veyrieras algorithm in BioMercator v4.2.3. The optimal M-QTL model for each chromosome was selected based on multiple model selection criteria, including the Akaike Information Criterion (AIC), corrected AIC (AICc), AIC3, Bayesian Information Criterion (BIC), and Average Weight of Evidence (AWE). Sensitivity analysis on chromosome 7 showed that M-QTL peak positions were largely robust to assumptions regarding missing LOD and R^2^ values and physical-to-genetic distance conversion. Comparison of the baseline scenario (S0; 200 kb cM^−1^) with ten alternative scenarios (S1–S10) spanning 150–250 kb cM^−1^ revealed tight clustering of biologically plausible scenarios, with larger positional shifts occurring only under compounded extreme assumptions. Although minor positional shifts and occasional loss or emergence of individual M-QTLs were observed, the major hotspot regions were conserved across non-extreme scenarios. Among the parameters tested, variation in physical-to-genetic distance conversion had the greatest influence on M-QTL peak positions, followed by assumed R^2^ values, whereas changes in LOD thresholds had minimal impact. Consistent with baseline confidence intervals, M-QTLs 7.1–7.6 and 7.8 showed high positional stability, with M-QTLs 7.4 and 7.6 being the most stable, whereas M-QTLs 7.7 and 7.9 were more CI-sensitive and M-QTLs detected only under extreme assumptions were least stable (Fig. S1a & S1b).

**Fig. 1:**
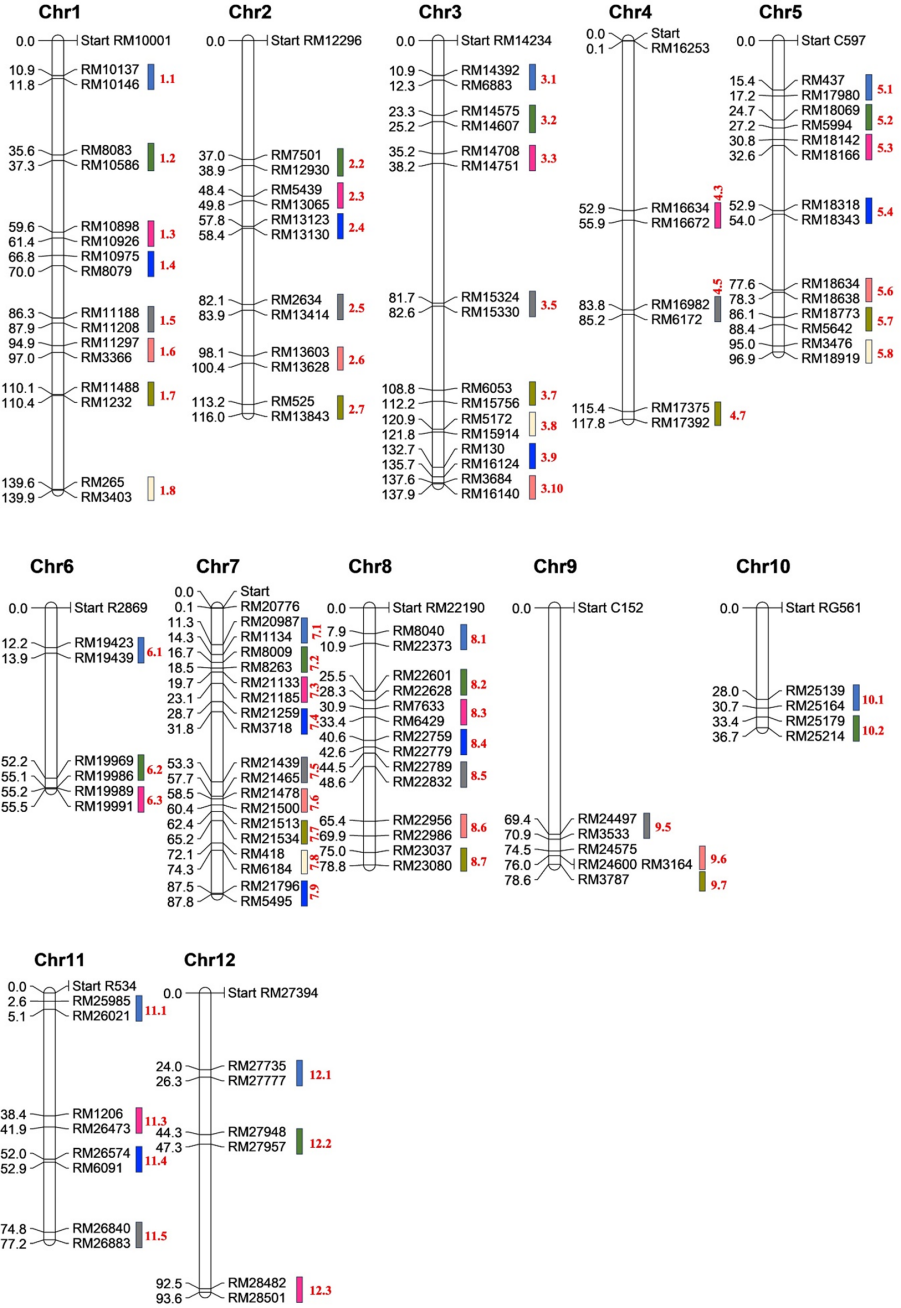
Distribution of Fe-toxicity tolerance–related M-QTLs across the rice genome. Each vertical bar represents a rice chromosome, with chromosome numbers indicated at the top. A total of 63 M-QTLs is shown as coloured bars along the respective chromosomes, with their names and corresponding positions (in cM) marked. The CIs for each M-QTL are also indicated.

The identified 85 M-QTLs were distributed across all 12 rice chromosomes, with the number of M-QTLs per chromosome ranging from three on chromosomes 6 and 10 to ten on chromosome 3 (Table S2). To focus on statistically stable M-QTLs, only those comprising a minimum of two QTLs were retained^70,71^ (Fig. 1), reducing the total count to 63 M-QTLs. The R^2^ values of the 63 M-QTLs ranged from 7% to 31% (Table 2), with M-QTL 3.7 exhibiting the highest value (31%), followed by M-QTL 1.7 (23.13%), M-QTL 1.6, and M-QTL 7.6 (15%). The CIs of the M-QTLs spanned 0.21 cM (M-QTL 3.10) to 4.48 cM (M-QTL 8.6), with a mean of 2.12 cM. Corresponding physical intervals varied from 53.6 kb to 1.12 Mb, with an average of 0.581 Mb. Notably, all 63 M-QTLs had CIs smaller than 5 cM, and 54 M-QTLs (85.71%) exhibited CIs below 3 cM. Furthermore, all 63 M-QTLs spanned physical distances less than 2 Mb, as inferred from the positions of flanking markers. Applying prioritisation criteria commonly used in M-QTL analyses—cluster size ≥ 5, estimated R^2^ ≥10%, and ≥ 5 nested CGs—we identified 10 M-QTLs that were designated as high-confidence for focused interpretation: 7.2, 7.3, 1.6, 2.2, 6.2, 7.4, 7.1, 2.6, 3.1, and 9.6 (Table 2). M-QTLs not meeting these prioritisation criteria were retained for downstream analyses and discussion, and several were found to harbour biologically relevant CGs or exhibit relatively high effect estimates, indicating that the ‘high-confidence’ designation reflects prioritisation rather than exclusion. For example, lowering the cluster size threshold from ≥ 5 to ≥ 3 would expand the high-confidence M-QTL set from 10 to 29 regions, including M-QTLs such as 3.7, 11.1, 9.7, and 2.7 that exhibit high R^2^ or substantial CG content (Table S3). A summary of M-QTL characteristics, including contributing QTL number, trait representation, effect size estimates, and CG density, is provided in Table S4 to facilitate transparent interpretation of M-QTL prioritisation.

**Table 2:** List of 63 M-QTLs shortlisted based on the number of overlapping QTLs.

S. no.	M-QTL	Start marker	Start position (cM)	Stop marker	Stop position (cM)	CI (95%; cM)	Mean R2	Cluster size	bp Start	bp Stop	Physical interval (bp)	Coding genes	CGs	CGs ≥2
1	1.1	RM10137	10.87	RM10146	11.83	0.96	16.83	6	2,740,418	2,979,574	239,156	58	11	3
2	1.2	RM8083	35.57	RM10586	37.31	1.74	10.00	3	8,915,741	9,349,811	434,070	64	22	3
3	1.3	RM10898	59.63	RM10926	61.43	1.80	8.33	3	14,906,908	15,395,949	489,041	58	13	3
4	1.4	RM10975	66.81	RM8079	69.96	3.15	11.00	3	16,720,237	17,514,968	794,731	47	11	5
5	1.5	RM11188	86.29	RM11208	87.86	1.57	16.67	9	21,596,783	21,991,292	394,509	52	10	3
6	1.6	RM11297	94.94	RM3366	96.95	2.01	20.00	7	23,760,658	24,263,311	502,653	74	29	8
7	1.7	RM11488	110.14	RM1232	110.42	0.28	23.13	8	27,559,809	27,632,712	72,903	7	0	0
8	1.8	RM265	139.62	RM3403	139.87	0.25	17.00	2	34,995,785	35,196,573	200,788	29	7	4
9	2.2	RM7501	36.95	RM12930	38.85	1.90	11.14	7	9,244,171	9,717,969	473,798	51	14	8
10	2.3	RM5439	48.38	RM13065	49.75	1.37	17.29	7	12,099,918	12,444,539	344,621	26	4	2
11	2.4	RM13123	57.79	RM13130	58.38	0.59	10.00	2	14,328,767	14,494,685	165,918	7	0	0
12	2.5	RM2634	82.06	RM13414	83.92	1.86	11.25	4	20,495,111	20,960,569	465,458	65	11	4
13	2.6	RM13603	98.08	RM13628	100.39	2.31	15.86	4	24,500,209	25,112,534	612,325	83	23	5
14	2.7	RM525	113.15	RM13843	115.95	2.80	10	3	28,267,534	28,967,074	699,540	114	37	12
15	3.1	RM14392	10.94	RM6883	12.32	1.38	10.29	5	2,751,406	3,097,333	345,927	71	18	8
16	3.2	RM14575	23.33	RM14607	25.16	1.83	8.00	3	5,863,396	6,410,992	547,596	85	22	11
17	3.3	RM14708	35.19	RM14751	38.15	2.96	10.67	3	8,826,746	9,566,506	739,760	127	33	10
18	3.5	RM15324	81.73	RM15330	82.59	0.86	9.50	2	20,663,700	20,915,537	251,837	25	4	2
19	3.7	RM6053	108.75	RM15756	112.22	3.47	31.00	3	27,380,880	28,252,864	871,984	133	31	9
20	3.8	RM5172	120.94	RM15914	121.77	0.83	12.25	4	30,421,880	30,653,694	231,814	37	10	4
21	3.9	RM130	132.72	RM16124	135.72	3.00	8.50	2	33,386,334	34,186,357	800,023	144	41	16
22	3.10	RM3684	137.64	RM16140	137.85	0.21	7.00	3	34,616,140	34,669,742	53,602	11	0	0
23	4.3	RM16634	52.9	RM16672	55.87	2.97	13.50	2	13,214,340	13,955,757	741,417	50	10	4
24	4.5	RM16982	83.83	RM6172	85.24	1.41	10.00	10	20,852,006	21,179,535	327,529	32	5	1
25	4.7	RM17375	115.35	RM17392	117.75	2.40	10.00	2	28,628,544	29,328,213	699,669	87	24	7
26	5.1	RM437	15.38	RM17980	17.24	1.86	13.33	3	3,876,201	4,492,917	616,716	61	18	5
27	5.2	RM18069	24.72	RM5994	27.15	2.43	10.00	3	6,149,127	6,883,785	734,658	97	23	7
28	5.3	RM18142	30.78	RM18166	32.57	1.79	15.00	2	7,805,524	8,252,621	447,097	30	5	2
29	5.4	RM18318	52.87	RM18343	54	1.13	10.00	3	13,220,510	13,649,242	428,732	43	6	2
30	5.6	RM18634	77.6	RM18638	78.28	0.68	8.50	4	19,427,973	19,662,062	234,089	31	10	3
31	5.7	RM18773	86.08	RM5642	88.37	2.29	9.20	5	21,611,552	22,184,500	572,948	75	16	4
32	5.8	RM3476	95.01	RM18919	96.87	1.86	11.00	2	23,843,930	24,310,982	467,052	86	18	6
33	6.1	RM19423	12.22	RM19439	13.86	1.64	10.00	2	3,184,364	3,595,262	410,898	72	20	8
34	6.2	RM19969	52.19	RM19986	55.07	2.88	10.28	7	13,338,527	13,908,619	570,092	67	13	5
35	6.3	RM19989	55.15	RM19991	55.53	0.38	9.5	4	13,908,619	14,253,381	344,762	16	5	0
36	7.1	RM20987	11.32	RM1134	14.29	2.97	10.00	6	2,834,189	3,592,890	758,701	95	24	5
37	7.2	RM8009	16.71	RM8263	18.54	1.83	11.10	10	4,196,961	4,655,195	458,234	62	12	5
38	7.3	RM21133	19.71	RM21185	23.14	3.43	10.22	9	4,946,994	5,873,598	926,604	137	27	11
39	7.4	RM21259	28.68	RM3718	31.75	3.07	10.83	6	7,179,993	7,956,359	776,366	86	17	5
40	7.5	RM21439	53.3	RM21465	57.71	4.41	9.66	3	13,412,412	14,532,666	1,120,254	57	14	1
41	7.6	RM21478	58.52	RM21500	60.44	1.92	20.00	6	14,734,925	15,334,093	599,168	40	5	1
42	7.7	RM21513	62.35	RM21534	65.23	2.88	9.50		15,691,452	16,495,963	804,511	67	7	0
43	7.8	RM418	72.11	RM6184	74.33	2.22	8.00	4	18,132,231	18,688,317	556,086	71	11	1
44	7.9	RM21796	87.52	RM5495	87.79	0.27	11.00	2	21,984,503	22,052,037	67,534	10	4	0
45	8.1	RM8040	7.94	RM22373	10.88	2.94	11.00	2	2,016,695	2,833,539	816,844	94	20	9
46	8.2	RM22601	25.54	RM22628	28.34	2.80	10.40	5	6,418,791	7,225,706	806,915	34	0	0
47	8.3	RM7633	30.91	RM6429	33.41	2.50	10.00	4	7,760,949	8,384,503	623,554	65	7	4
48	8.4	RM22759	40.63	RM22779	42.61	1.98	10.00	6	10,189,953	10,780,912	590,959	56	7	3
49	8.5	RM22789	44.52	RM22832	48.58	4.06	12.33	3	11,101,911	12,179,264	1,077,353	78	10	0
50	8.6	RM22956	65.43	RM22986	69.91	4.48	13.50	2	16,388,784	17,508,990	1,120,206	86	9	1
51	8.7	RM23037	74.98	RM23080	78.78	3.80	12.00	3	18,760,835	19,728,003	967,168	105	21	9
52	9.5	RM24497	69.36	RM3533	70.94	1.58	8.50	2	17,494,548	17,887,590	393,042	53	20	8
53	9.6	RM24575	74.51	RM24600	75.98	1.47	10.00	5	18,867,627	19,385,696	518,069	83	23	6
54	9.7	RM3164	76.03	RM3787	78.59	2.56	10.00	4	18,920,684	20,043,308	1,122,624	134	35	11
55	10.1	RM25139	27.95	RM25164	30.66	2.71	13.60	5	7,268,620	8,120,494	851,874	56	12	2
56	10.2	RM25179	33.36	RM25214	36.66	3.30	10.00	3	8,764,608	9,636,389	871,781	74	11	4
57	11.1	RM25985	2.64	RM26021	5.12	2.48	14.33	3	720,560	1,443,712	723,152	114	31	10
58	11.3	RM1206	38.43	RM26473	41.89	3.46	11.50	2	9,761,843	10,626,826	864,983	52	6	1
59	11.4	RM26574	52.02	RM6091	52.92	0.90	14.40	5	13,179,085	13,405,451	226,366	8	0	0
60	11.5	RM26840	74.78	RM26883	77.2	2.42	10.00	2	18,954,706	19,614,942	660,236	85	11	5
61	12.1	RM27735	23.95	RM27777	26.34	2.39	8.00	2	5,829,022	6,643,628	814,606	54	7	3
62	12.2	RM27948	44.28	RM27957	47.26	2.98	7.00	2	10,972,098	11,871,717	899,619	50	11	2
63	12.3	RM28482	92.47	RM28501	93.61	1.14	8.50	2	23,106,391	23,406,780	300,389	49	10	3

### Discovery of CGs within M-QTL regions

**Fig. 2:**
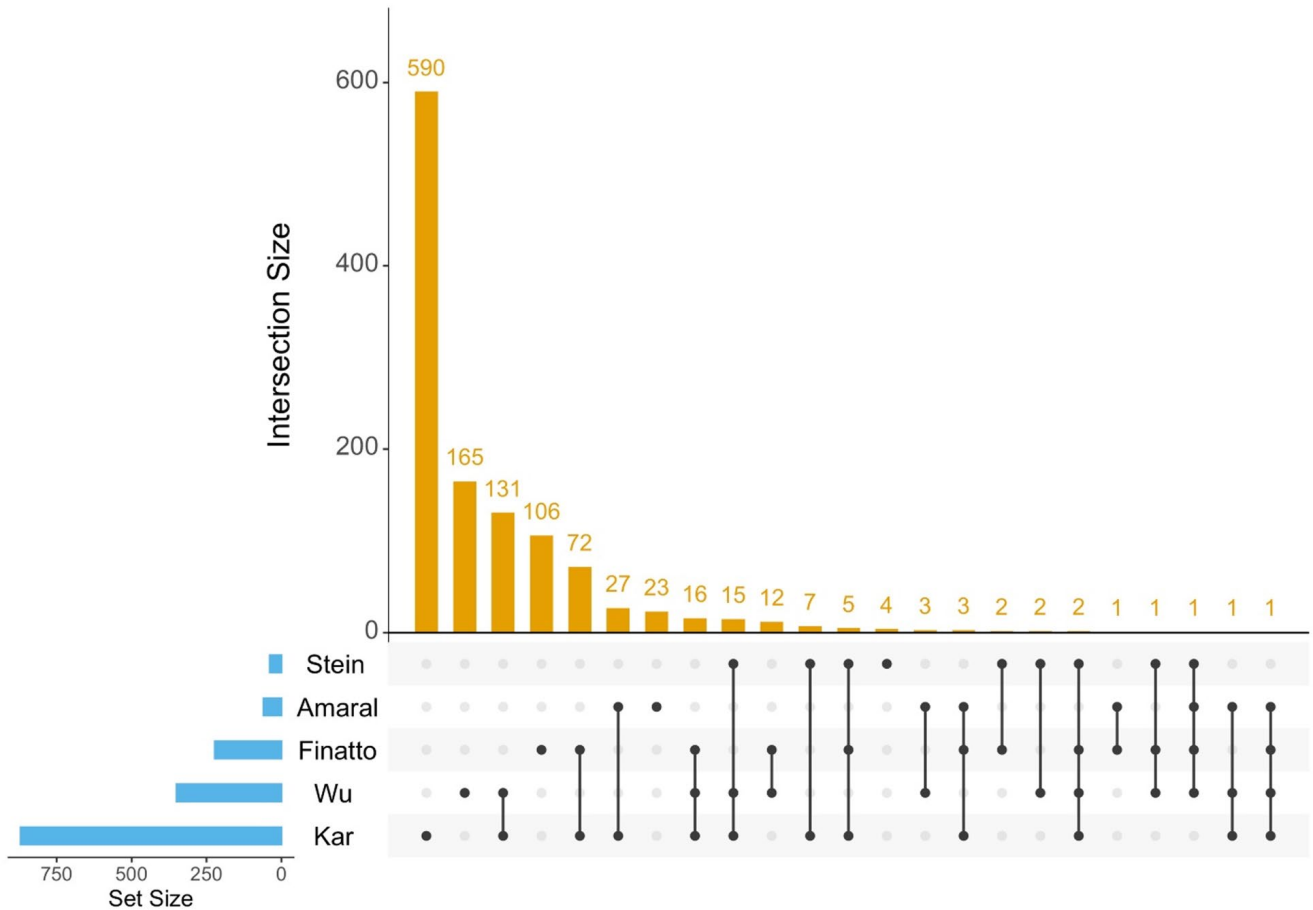
Upset plot showing the overlap of differentially expressed genes (DEGs) identified across five independent transcriptome studies^19,50–53^. Blue bars indicate the total number of DEGs reported in each study, while yellow bars represent the genes shared between the present analysis and these studies.

**Fig. 3:**
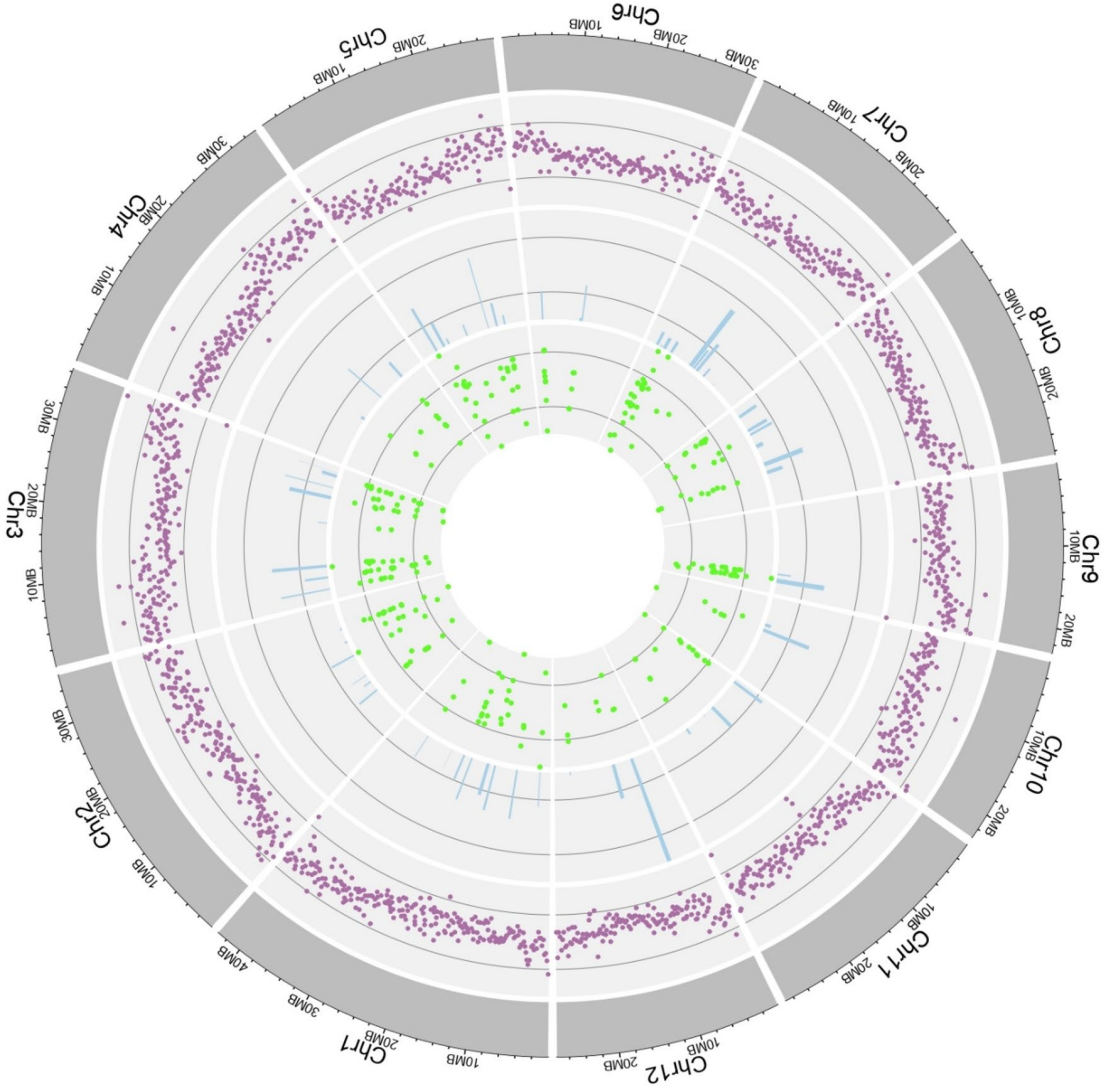
Distribution patterns of gene density, QTLs, M-QTLs, M-QTL–spanning genes, and CGs across the rice genome. The outermost circle represents the rice chromosomes with genomic positions indicated in megabases (Mb). The second circle, shown in purple, depicts gene density across the genome. The third and fourth inner circles represent the number of initial Fe-toxicity tolerance QTLs and the identified M-QTL–associated genes, respectively. The fifth (innermost) circle highlights the 84 CGs underlying the M-QTL regions.

A total of 4,070 non-redundant genes located within the 63 promising M-QTL regions were retrieved from the RAP database and considered for subsequent analyses (Table S5). Among these, M-QTL 9.7 contained the highest number of genes (207), followed by M-QTL 3.9 (144) and M-QTL 7.3 (137), whereas M-QTL 1.7 and M-QTL 11.4 harboured the fewest (7 and 8 genes, respectively) (Table 2). To refine this set, gene expression profiles were examined using five transcriptomic datasets generated for the Fe toxicity response in different rice genotypes^20,51–54^. CGs were prioritised based on differential expression in at least two independent RNA-seq datasets to balance robustness and biological inclusiveness. More permissive (≥ 1 dataset) or stringent (≥ 3 datasets) thresholds were evaluated but not adopted due to concerns of interpretability and excessive conservatism, respectively. This analysis identified 897 genes that were differentially expressed in at least one study, of which 284 genes exhibited differential expression in two or more studies and were designated as high-confidence CGs for further analysis, supported by transcriptome overlap and genome-wide distribution patterns (Figs. 2 and 3). Notably, 60 (21.12%) of these high-confidence CGs were located on chromosome 3, including 16 genes within M-QTL 3.9, whereas no CGs were detected within M-QTLs 1.7, 2.4, 3.10, 7.7, 7.9, 8.2, 8.5, and 11.4 (Fig. S2).

The 284 CGs included several well-characterised Fe toxicity-responsive genes, such as *NRAMP6*, *OsGRX9*, *OsPEZ1*, *OsFRO2*, and *OsZIP8*. Other CGs were implicated in detoxification, including *OsGSTF5*, *OsGSTF9*, and *OsGSTF10* (M-QTL 1.3); protein folding and stress response, such as *OsMSR3*, *OsHSP70*, and *OsHsp17.4* (M-QTL 3.3); and various transport processes, including *OsPDR8*, *OsPDR9*, *OsPDR10* (M-QTL 1.6); *OsPT2*, *OsHMP18*, *OsZNL* (M-QTL 3.1); *OsPEZ1* (M-QTL 3.5); *OsSPX-MFS1* (M-QTL 4.7); *OsZIP8* (M-QTL 7.4); and *OsFRDL2* (M-QTL 10.1). Several TF genes were also identified, including *OsHsfA7* (M-QTL 1.5), *OsMYB30* (M-QTL 2.7), *OsATH1* (M-QTL 7.2), *OsERF48/OsDRAP1* (M-QTL 8.7), and multiple WRKY and NAC genes such as *OsNAC10*, *OsNAC122*, *OsNAC045*, *OsWRKY46*, *OsWRKY89*, and *OsWRKY50* (M-QTL 11.1). Hormone-related genes were also represented, including *OsACS2* (M-QTL 4.7), *OsIAA20* (M-QTL 6.1), *OsIAA21* (M-QTL 6.2), *OsPILS1* (M-QTL 9.6), and *OsAAO1* (M-QTL 7.1). Additionally, genes associated with chloroplast development and photosynthesis, such as *Os3BGlu6* (M-QTL 3.2), *OsASS1* (M-QTL 3.7), *OsChlD* (M-QTL 3.9), *VIRESCENT 3* (M-QTL 6.1), *OsMORF9* (M-QTL 8.1), and *PsbP* (M-QTL 12.3), were identified. These CGs were prioritised based on their functional annotations and recurrence across multiple expression datasets; however, the potential contributions of the 114 unannotated genes within these regions to Fe toxicity tolerance cannot be excluded.

### Promoter analysis

**Fig. 4:**
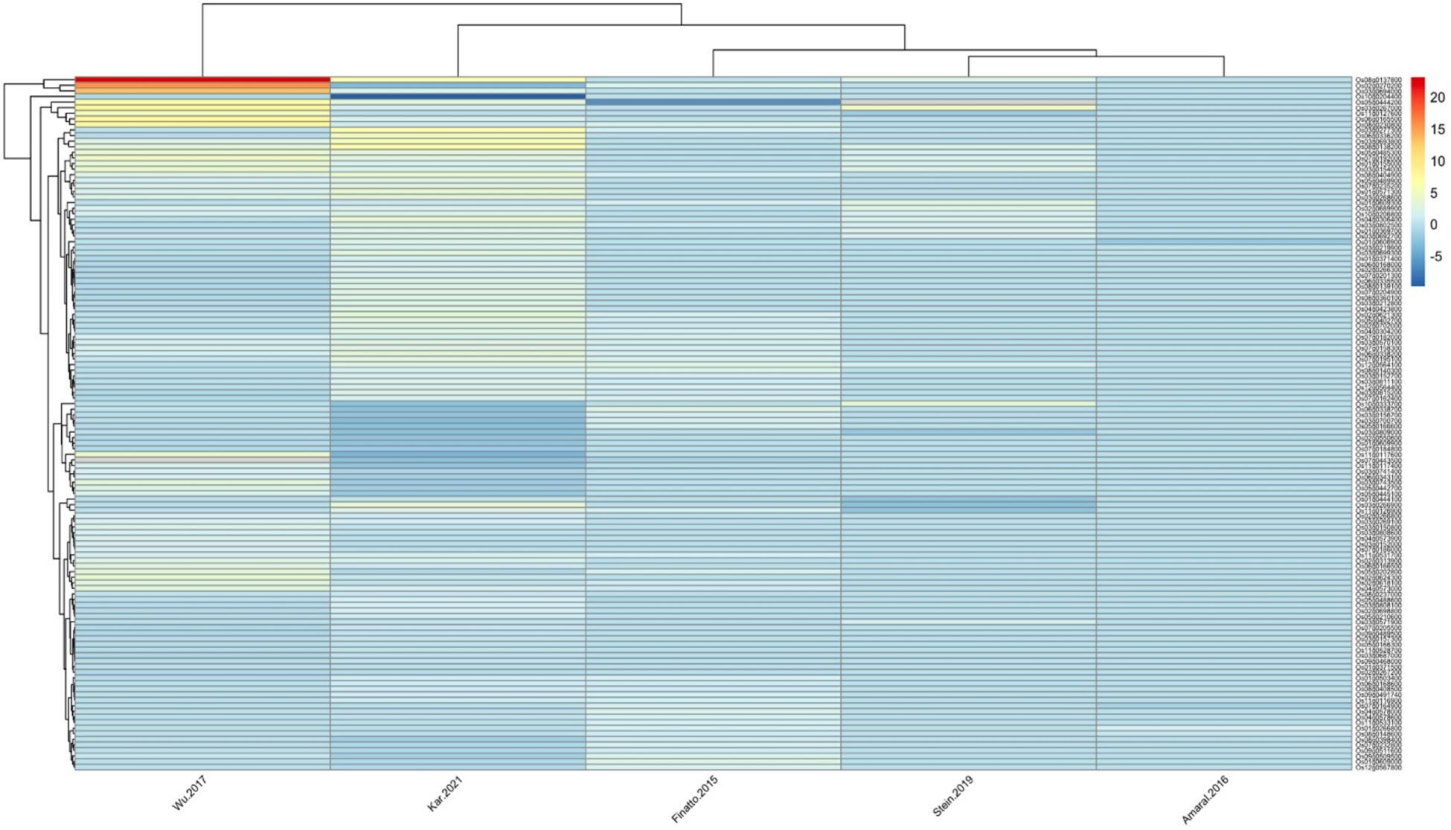
Heatmap showing the in-silico identification of cis-regulatory elements within the putative promoter regions of 284 Fe-toxicity tolerance–responsive genes. The colour scale indicates the relative abundance of individual regulatory elements across genes.

Promoter analysis of the 284 prioritised CGs revealed a high prevalence of cis-regulatory elements (CREs) associated with stress- and hormone-responsive transcriptional regulation (Fig. 4). MYB-related elements were detected in all CGs, encompassing MYB, MYB-like, MBS, MBSI, MRE, and related MYB-binding motifs. Abscisic acid–responsive elements (ABREs), including ABRE, ABRE2, ABRE3a, and ABRE4, were present in 259 CGs.

In addition, auxin-responsive elements (AuxRE/AuxRR-core) were identified in 48 CGs, while drought-responsive elements (DRE1/DRE-core) occurred in 145 CGs. General stress-associated regulatory motifs were also widely distributed, with stress response elements (STRE) detected in 257 CGs, MYC-binding elements in 279 CGs, and W-box motifs in 176 CGs (Fig. S3). Collectively, the promoter regions of CGs were enriched for CREs linked to stress signalling, hormone responsiveness, and transcription factor binding. A complete catalogue of identified CREs, motif sequences, and their distribution across CGs is provided in the Table S6.

### PPI network and gene ontology analysis

**Fig. 5:**
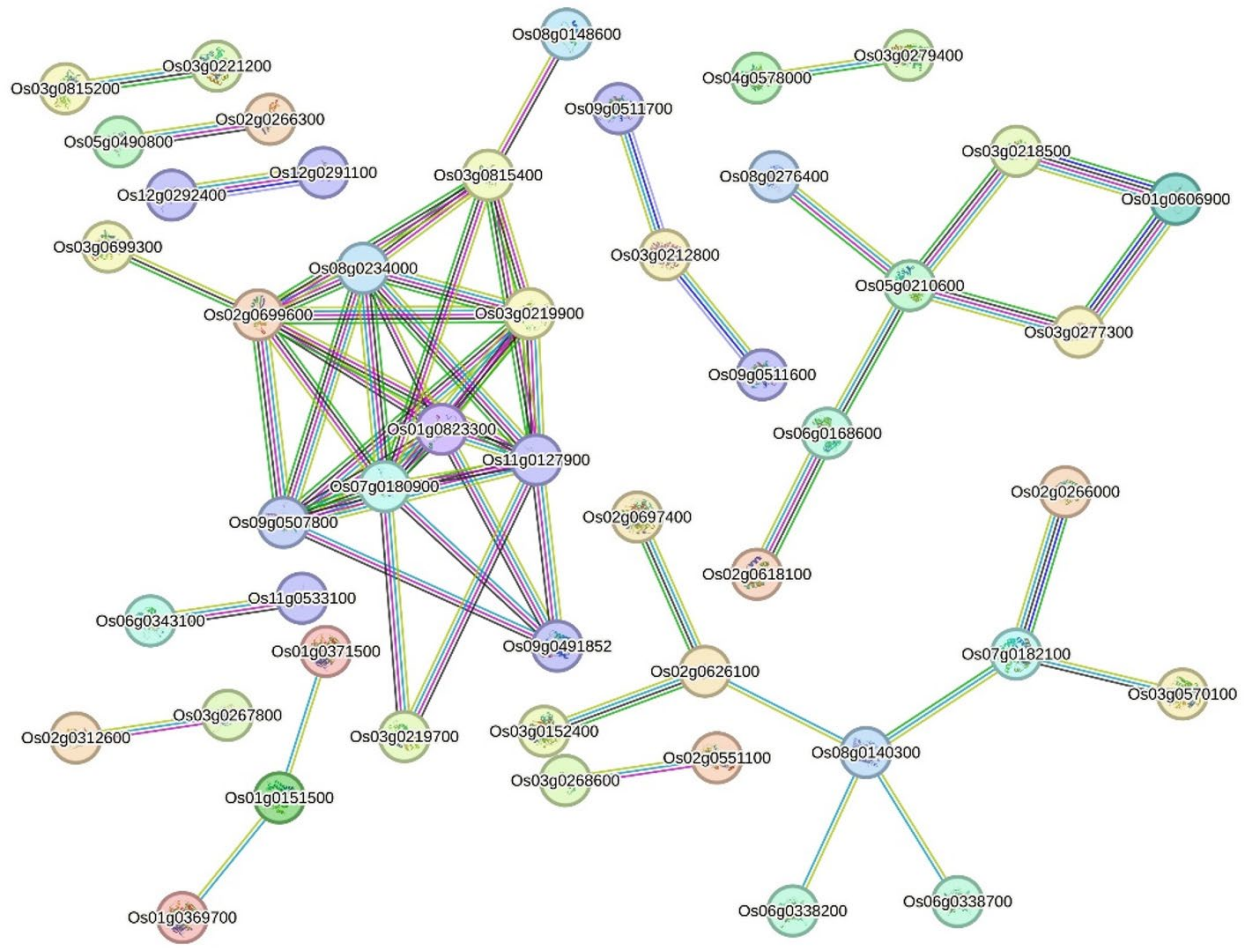
PPI network of 284 CGs derived from Fe-related M-QTLs, constructed using a minimum required interaction score of 0.7.

To investigate the functional connectivity among CGs associated with iron toxicity tolerance, a PPI network was constructed using the protein sequences of 284 CGs located within Fe-toxicity-related M-QTL regions (Fig. 5). Clustering using the Markov Cluster Algorithm (MCL) with an inflation parameter of 5 revealed multiple distinct modules. The largest, Cluster 1, comprised 14 proteins, predominantly ribosomal subunits (e.g., Os01g0823300, Os03g0219900), suggesting a central role in protein synthesis. Cluster 2, containing 13 proteins, was enriched in transcriptional regulators, notably Os02g0266800, a bZIP transcription factor (RISBZ3). Cluster 4, with eight proteins, included Os02g0626100, encoding phenylalanine ammonia-lyase (PAL), a key enzyme of the phenylpropanoid pathway, implicating this cluster in lignin biosynthesis and cell wall fortification, which are essential for structural reinforcement and oxidative stress mitigation. Cluster 5, comprising seven proteins, contained Os03g0152700, a pseudouridylate synthase, indicating potential involvement in RNA processing and post-transcriptional regulation. Several smaller clusters (3–6 members) harboured critical stress-responsive genes, including *Os10g0531400* (*OsGSTU10*), *Os05g0574600* (thioredoxin-like protein), and *Os06g0218300* (peroxidase precursor), which are implicated in detoxification, antioxidant defence, and redox homeostasis. Additional minor clusters contained genes related to hormonal signalling, metal transport, and ubiquitin-mediated protein degradation.

**Fig. 6:**
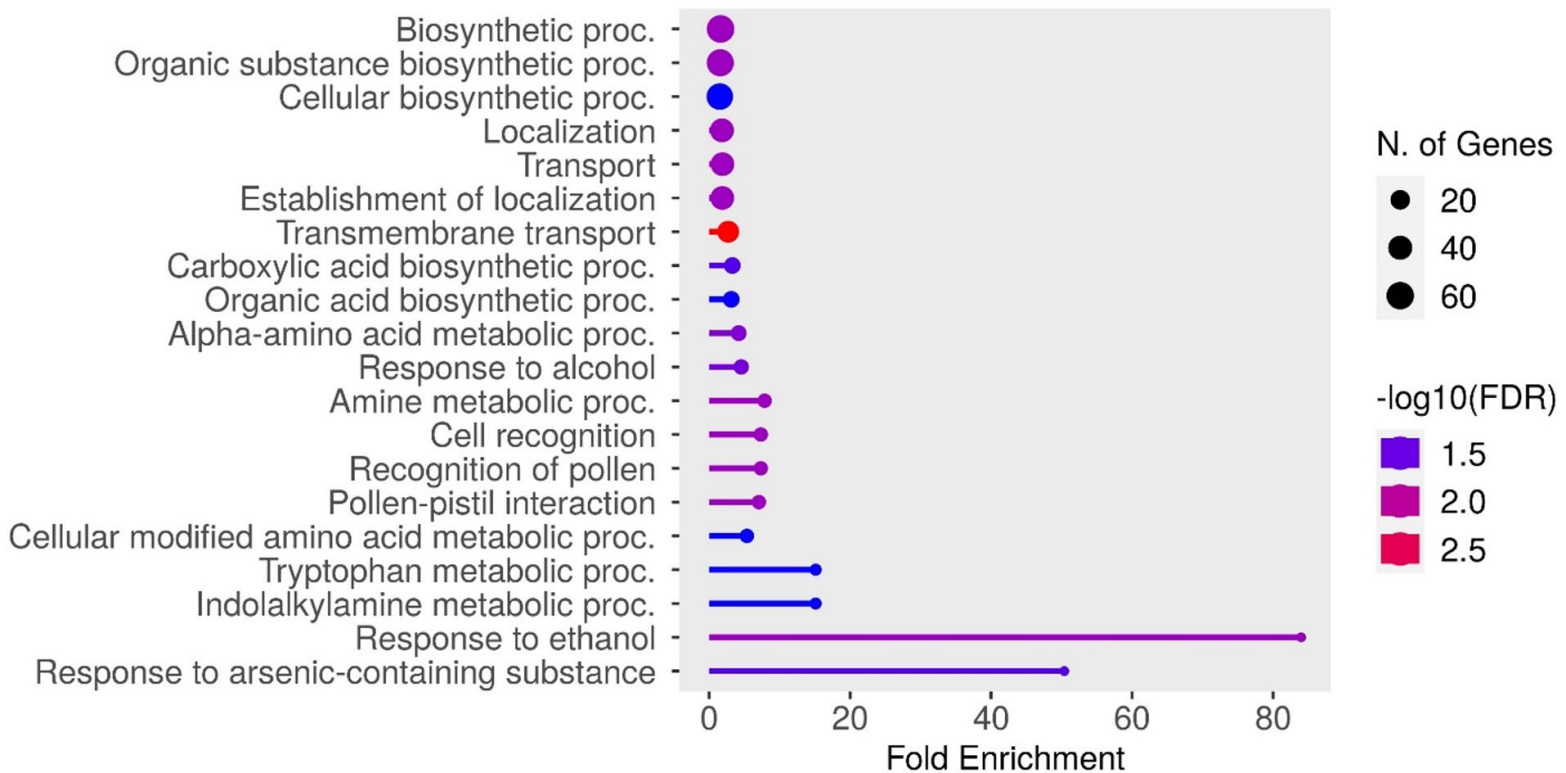
GO enrichment analysis of 284 candidate genes associated with Fe-toxicity tolerance, showing significantly enriched biological processes (FDR < 0.05).

Gene Ontology (GO) analysis revealed diverse functional roles for the 284 CGs (Fig. 6). Among these, 29 CGs were predicted to be associated with localization, transport, and transmembrane transport, including *OsPDR8*, *OsPDR9*, *OsPDR10*, *OsNRAMP6*, *OsPT2*, *OsZIP8*, *OsTIP2;2*, *OsFRDL2*, *OsPEZ1*, *OsMATE35*, *OsHMP18*, *OsHMP39*, *SWEET6A*, *OsNRT1.5 A*, *OsMST4*, *OsPUP2*, and *OsPUP3*, among others. Several CGs were linked to biosynthetic processes, such as *OsTCD3*, *OsASS1*, *OsCesA2*, *V3*, *OsChlD*, *OsACS2*, *OsIAA20*, and *OsIAA21*. Other CGs were involved in metal sequestration and detoxification, including *OsGSTF5*, *OsGSTF9*, and *OsGSTF10* (Fig. 6; Table S7). Furthermore, groups of CGs were associated with Fe toxicity responses, encompassing ROS scavenging, stress perception and signalling, cell wall modification, chloroplast development, and regulation of photosynthesis (Table S7).

### CGs localization, and identification of TFs and transporters among the CGs

**Fig. 7:**
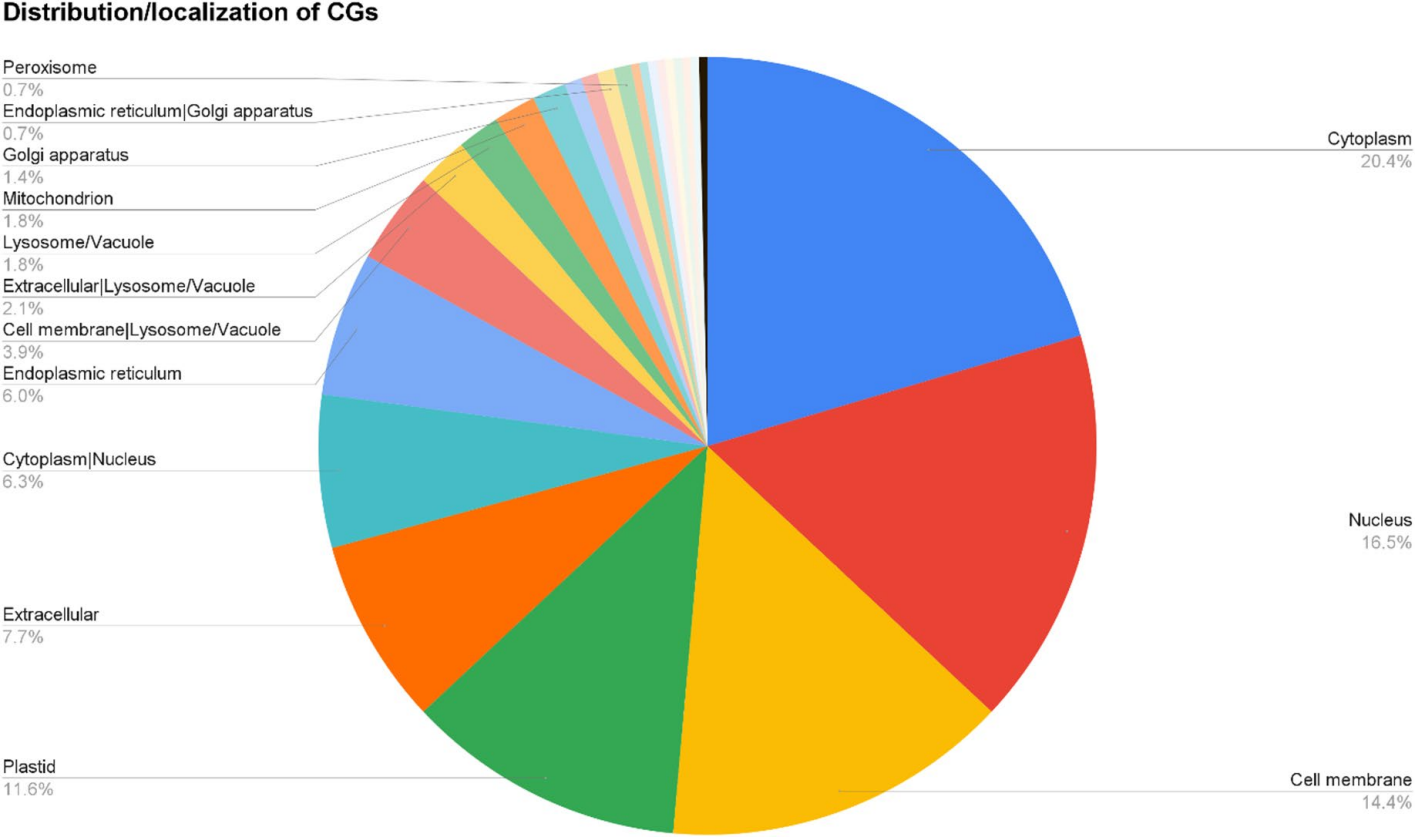
Predicted subcellular localization of proteins encoded by the 284 candidate genes derived from Fe-related M-QTLs.

The 284 CGs were further analysed for subcellular localisation using DeepLoc v2.0. The majority were predicted to localise to the cytoplasm (58 CGs), followed by the nucleus (47 CGs), cell membrane (41 CGs), and plastid (33 CGs) (Fig. 7). Among these CGs, 21 were identified as encoding TFs, including five WRKYs, three NACs, three bZIPs, three MYBs, and members of other TF families such as ERF, bHLH, and C2H2 (Table S8). Analysis using DeepTMHMM revealed 32 CGs with six or more transmembrane (TM) domains, with a maximum of 12 TM domains observed in 14 CGs (Table S9). These genes represent diverse functional categories, including heavy metal transporter genes (*OsNRAMP6*, *OsFRO2*, *OsZIP8*, *OsPEZ1*, *OsFRDL2*), drug/metabolite transporter genes (*OsPDR10*, *OsPDR9*, *OsPDR8*, *OsMATE35*), and nutrient transporter genes (*OsNRT1.5*, *PTH1-2*, *OsMST4*, *OsSTP4*, *OsNPF2.4*, *OsPTR2*, *OsSWEET6a*, *OsZNL*, *OsSPX-MFS1*). Additional transporter genes, such as *OsTIP2;2*, were also detected.

### Candidate gene-based association mapping for Fe toxicity tolerance

The 284 CGs harbored a total of 1,018 SNPs across the 551 rice accessions and were used for association analysis employing two models, FarmCPU and BLINK, implemented in GAPIT. Phenotypic data reported by Miao and his coworkers^61^ for shoot height (SH), shoot fresh weight (SFW), and root length (RL) under Fe-toxic conditions (suffix “_Fe”), their respective control conditions (“_CK”), and three tolerance indices, including relative shoot height (RSH), relative shoot fresh weight (RSFW), and relative root length (RRL), were analyzed to capture differential responses to Fe toxicity stress.

**Fig. 8:**
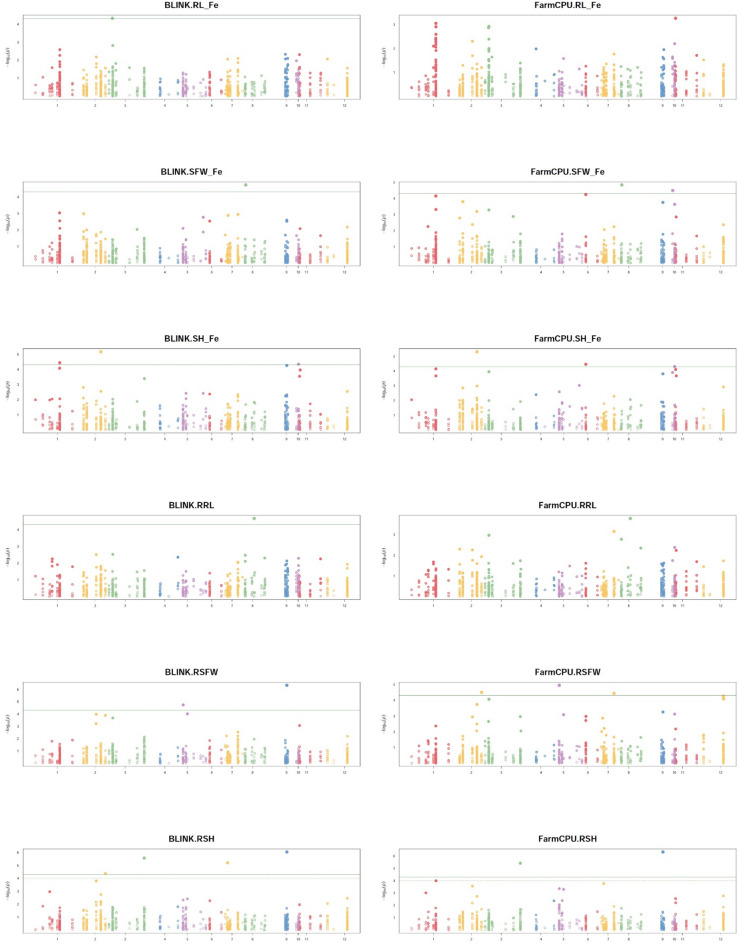
Comparative Manhattan plots of candidate CG-AS for Fe-toxicity tolerance traits in rice using BLINK and FarmCPU models. Each panel depicts CG-AS results for Fe-toxicity tolerance–related traits and their indices, with rows representing individual traits (RL_Fe, SFW_Fe, SH_Fe, RRL, RSFW, and RSH) and columns corresponding to the association models used. The x-axis represents the 12 rice chromosomes, while the y-axis shows the –log₁₀(p-value) of marker–trait associations. The green horizontal line indicates the significance threshold for detecting significant MTAs. Comparative visualization highlights model- and trait-specific differences in the number, magnitude, and chromosomal distribution of significant associations, providing insights into the genetic architecture underlying Fe-toxicity tolerance in rice.

The CG-based association study identified 27 significant MTAs across the six traits and their derived indices (Table S10). Among these, 13 MTAs were specific to Fe toxicity stress, 13 were associated with control conditions, and one MTA was common to both conditions (Fig. 8). Under control conditions, 8, 1, and 1 MTAs were identified solely for SH, SFW, and RL, respectively, while 3 MTAs were pleiotropic for SH and SFW. Since the focus of this study was on genes related to Fe toxicity response, MTAs identified under control conditions were excluded from downstream analyses. Under Fe-toxic conditions, a total of 4, 4, 2, 4, 1, and 1 MTAs were identified for SH, RSH, SFW, RSFW, RL, and RRL, respectively.

In total, four MTAs i.e. MTA1 (M-QTL1.6), MTA6 (M-QTL2.6), MTA16 (M-QTL6.1), and MTA26 (M-QTL10.2) were associated with shoot height (SH) under Fe-toxicity conditions, corresponding to *OsRPK1*, *Os02g0620400*, *OsFbox298*, and *Os10g0329400* (hypothetical protein), respectively. Notably, MTA6 was detected by both association mapping models under Fe-toxic conditions, supporting its robustness. Similarly, four MTAs, i.e. MTA7 (M-QTL2.7), MTA12 (M-QTL3.9), MTA17 (M-QTL7.3), and MTA24 (M-QTL9.6) were associated with relative shoot height (RSH), corresponding to *OsWRKY66*, *Os03g0805766* (hypothetical protein), *OsFbox348*, and *OsCCR1*, respectively. Among these, MTA12 and MTA24 were consistently detected by both models. For shoot fresh weight (SFW), two MTAs were identified: MTA19 (M-QTL8.1; detected by both models) and MTA25 (M-QTL10.1), genic to *Os08g0148600* (hypothetical protein) and *OsFRDL2*, respectively. Four MTAs were associated with relative shoot fresh weight (RSFW), including MTA14 (M-QTL5.1; detected by both models) and MTA18, corresponding to *OsGLYI6* and *Os07g0431160* (unknown protein). The remaining two MTAs, MTA7 and MTA24, were pleiotropic: MTA7 was shared with RSH, and MTA24 was shared with both RSH and SFW, reflecting the functional interdependence of these shoot-related traits and their derived indices. One MTA each was identified for root length (RL) and relative root length (RRL): MTA8 (M-QTL3.1), genic to *Os3BGlu6*, and MTA20 (M-QTL8.4), genic to *Os08g0267300* (unknown protein). Interestingly, MTA20 was also detected for SFW and SH under control conditions, in addition to RRL, indicating pleiotropy. It was consistently detected by both models for SFW_CK and SH_CK, confirming its stability across traits and conditions. Notably, *Os3BGlu6*, a chloroplast β-glucosidase within MTA8, emerged as a hub gene based on PPI network connectivity metrics (Fig. 5). Additionally, CGs with MTAs such as MTA5 and MTA19 were identified as PPI hubs and contained haplotype SNPs.

In total, 13 CG-based MTAs associated with Fe toxicity were selected for haplotype analysis: 4 for SH_Fe (MTA1, 6, 16, 26); 2 for SFW_Fe (MTA19, 25); 1 for RL_Fe (MTA8); 1 for RRL (MTA20); 4 for RSFW (MTA7, 14, 18, 24); and 4 for RSH (MTA7, 12, 17, 24).

### Haplotype analysis of selected CGs involved in Fe toxicity tolerance

**Fig. 9:**
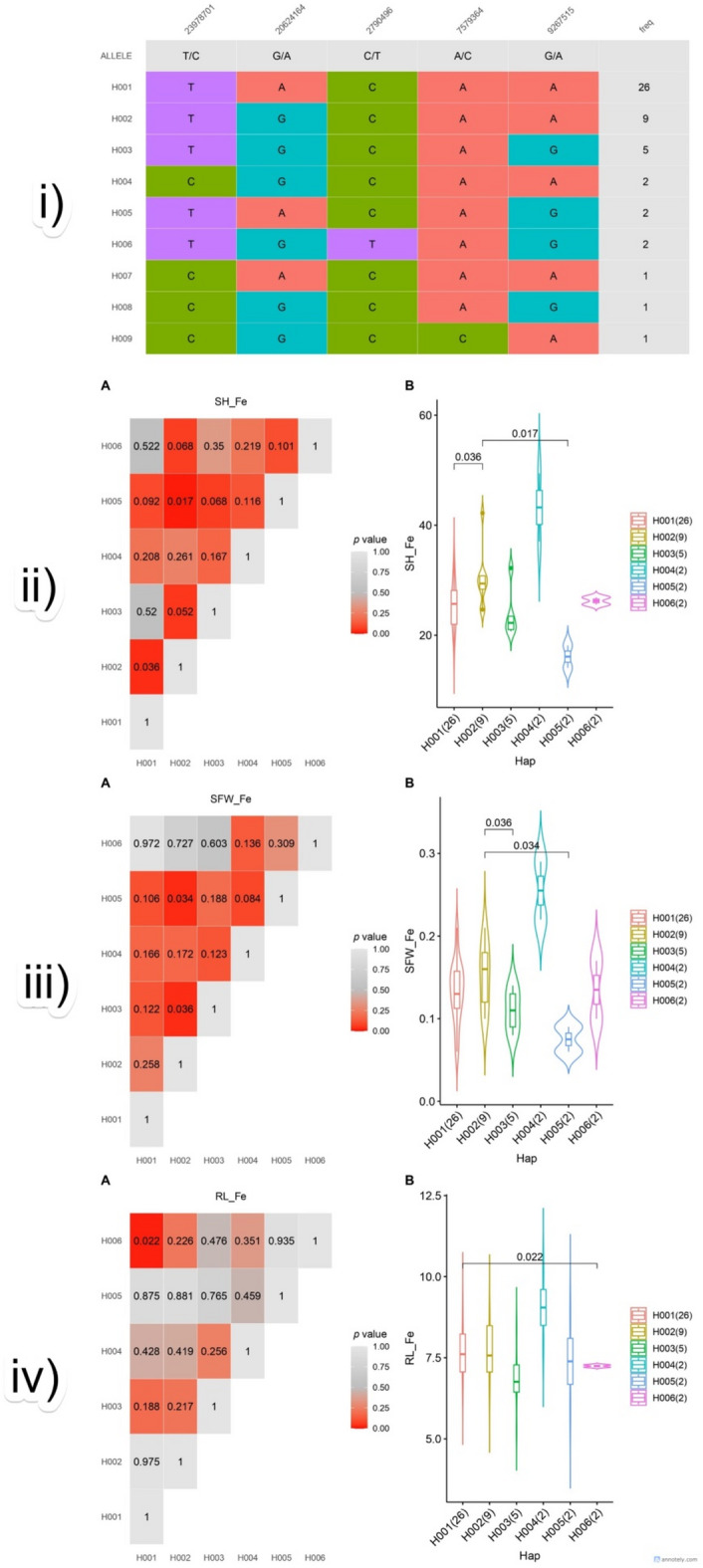
Haplotype-wise distribution of SH_Fe, RL_Fe, and SFW_Fe, and SNP-defined haplotype matrix across five genic loci associated with Fe-toxicity stress in rice. (i) The haplotype allele matrix depicts five trait-associated SNPs, with columns representing SNP positions and allelic states, and rows showing allele combinations defining each haplotype along with their genotype frequencies. These SNPs correspond to significant MTAs for Fe-toxicity tolerance traits and are located within or proximal to key candidate genes, including *OsRPK1*, *OsRLCK73*, and *OsFRDL2*. (ii–iv, **A**) Pairwise significance matrices (p-value heatmaps) illustrate statistical differences in trait performance among six major haplotypes (H001–H006) for SH_Fe, SFW_Fe, and RL_Fe under Fe-toxicity stress, where increasing red intensity denotes higher statistical significance (*p* < 0.05). (ii–iv, **B**) Corresponding violin–box plots show the distribution of each trait across haplotypes, with genotype counts indicated in parentheses. Horizontal bars and annotations denote significantly different pairwise comparisons based on ANOVA followed by post hoc tests.

Haplotype analysis was conducted on a subset of 49 rice genotypes from the 3k Rice Genome Project using 13 genic SNPs associated with iron toxicity tolerance. A total of nine distinct haplotypes (H001–H009) were identified across five SNPs (Fig. 9). Of these, six haplotypes (H001–H006), each represented by at least two genotypes, were considered for haplo-phenotypic association with traits evaluated under Fe toxicity stress, namely SH_Fe, RL_Fe, and SFW_Fe. Analysis of variance (ANOVA) detected significant differences among haplotypes for all three traits (SH_Fe: *p* = 0.036; RL_Fe: *p* = 0.022; SFW_Fe: *p* = 0.036), based on nominal p-values consistent with the targeted, CG–based analysis, confirming haplotype-specific effects on phenotypic responses to Fe stress. The exclusion of incomplete and heterozygous genotypes ensured a focus on homozygous haplotypes, thereby enhancing the reliability of the associations at the cost of reduced sample size (49). Haplotype groups varied in size (2–26 genotypes), reflecting the natural allele frequency distribution within the 3k Rice Genome panel.

Among the evaluated haplotypes, H004 consistently exhibited the highest mean values across all three traits and was represented by a limited number of accessions, consistent with rare but potentially favourable haplotypes observed in diverse rice panels. H002, present in nine genotypes, showed the second-highest performance across traits. In contrast, H001, the most common haplotype (*n* = 26), consistently ranked third. The remaining haplotypes (H003–H006) were associated with lower trait values, indicating limited relevance for improving Fe toxicity tolerance (Table 3).

**Table 3:** Haplotype distribution and associated tolerance responses under Fe toxicity stress.

Haplotype	Genotype count	SH_Fe rank	RL_Fe rank	SFW_Fe rank	Performance summary
H004	2	1st	1st	1st	Tolerance response, rare
H002	9	2nd	2nd	2nd	Tolerance response, moderate frequency
H001	26	3rd	3rd	3rd	Most frequent, moderately tolerant
H003	5	Lower	Lower	Lower	Sensitive response
H005	2	Lower	Lower	Lower	Sensitive response
H006	2	Lower	Lower	Lower	Sensitive response

These haplotypes were derived from five SNPs significantly associated with iron toxicity-related traits, identified through CG-based association studies (CG-AS). Each SNP was located within annotated gene regions and co-localised with previously reported M-QTLs for Fe toxicity tolerance (Table S7). Notably, MTA1 (Chr1:23978701; *p* = 3.57 × 10⁻⁵; MAF = 0.033), associated with SH_Fe, mapped to *Os01g0607900*, encoding a leucine-rich repeat receptor-like kinase (LRR-RLK), OsRPK1. MTA19 (Chr8:2790496; *p* = 1.45 × 10⁻⁵; MAF = 0.023), linked to SFW_Fe, overlapped with *Os08g0148600*, encoding an endoribonuclease Dicer homolog. MTA25 (Chr10:7579364; *p* = 3.23 × 10⁻⁵; MAF = 0.066), also associated with SFW_Fe, was located within *Os10g0206800*, a MATE transporter gene involved in aluminum-induced citrate efflux (*OsFRDL2*). Finally, MTA26 (Chr10:9267515; *p* = 4.46 × 10⁻⁵; MAF = 0.466), significantly associated with SH_Fe, mapped to *Os10g0329400*, a conserved hypothetical protein.

Importantly, several of the ten high-confidence M-QTLs, identified based on cluster size ≥ 5, R^2^ ≥10%, and ≥ 5 nested CGs, exhibited strong convergence across multiple analytical approaches, including significant MTAs, haplotype-tagging SNPs, and PPI hub genes. For instance, M-QTL 1.6 harboured MTA1, MTA2, and Hap-SNP1; M-QTL 2.6 included MTA6; M-QTL 3.1 contained MTA8 and a PPI hub; M-QTL 7.3 housed MTA17; and M-QTL 9.6 encompassed MTA24 along with another PPI hub.

## Discussion

### Challenges and advances in Fe toxicity tolerance in rice

Fe toxicity is a major constraint to rice production in lowland acidic soils, particularly in South Asia and sub-Saharan Africa. Although rice exhibits some tolerance to soil acidity, excessive Fe^2^⁺ under flooded conditions is readily absorbed, leading to oxidative stress through Fenton reactions and ultimately causing cellular damage^72^. Internal Fe accumulation disrupts nutrient uptake and inhibits cell division, particularly in roots^31^. As rice is a staple for over half the global population, Fe toxicity poses a serious threat to food security. Management practices such as intermittent drainage, liming^2^, silicon application^73^, and magnesium supplementation^74^ can mitigate Fe toxicity; however, breeding Fe-tolerant varieties remains the most sustainable long-term strategy.

The genetic basis of Fe toxicity tolerance in rice is complex. Numerous QTLs and CGs have been reported using intra- and inter-specific mapping populations^9,22,65,68,69,75–78^. However, no major QTL has been fine-mapped or cloned. Genes such as *OsFER1*, *OsFER2*, *OsVIT1*, *OsVIT2*, and *OsFRO1* have been implicated in Fe homeostasis and oxidative stress responses^78–80^. Variation in genotype performance across environments, influenced by stress severity and phenotyping methods, remains a major challenge^22^. Nonetheless, several consistent QTLs have been mapped on chromosomes 1 (36.8–41 Mb) and 3 (0–5 Mb)^22,30^. Early studies relied on bi-parental populations, which offered limited genetic diversity and low mapping resolution due to few markers and recombination events^80^. GWAS has helped overcome these limitations, enabling high-resolution mapping of Fe-related traits. Matthus et al. (2015)^32^ highlighted the importance of foliar redox balance, Li et al. (2019)^79^ linked GSNOR to root tolerance, and Zhang et al. (2017)^10^ identified 14 QTLs using 222 accessions. To date, 220 non-overlapping QTLs and 254 MTAs have been reported, primarily linked to traits such as LBI, LBS, RRE, RTI, and biomass, which are directly affected by Fe toxicity^31^.

Recently, Miao *et al*. (2024)^61^ conducted a GWAS on 551 accessions from the 3k Rice Genome Project^82^, identifying CGs and haplotypes and comparing physiological responses between tolerant and sensitive genotypes. Despite these advances, practical use of QTLs in breeding remains limited due to environmental instability, background effects, and linkage drag^31,83^. To our knowledge, this study represents the first comprehensive effort to define M-QTLs for Fe toxicity tolerance in rice, providing an invaluable resource for breeding Fe-tolerant varieties adapted to acidic soils.

### M-QTL analysis led to the identification of high-confidence candidate genomic regions associated with Fe toxicity response

Of the 474 reported QTLs for Fe toxicity–related traits in rice, 354 were successfully projected onto the consensus map, while ~ 25% remained unmapped, likely due to missing common markers or low marker density^34^. In this M-QTL analysis, heterogeneous Fe-related traits encompassing visual symptoms (leaf bronzing), biomass and growth parameters, physiological and biochemical responses, iron accumulation metrics, and composite tolerance indices were pooled into a single Fe toxicity tolerance category (FeTol). This strategy was adopted to enhance statistical power and enable consensus mapping across heterogeneous legacy datasets and does not presume a single or uniform genetic architecture underlying all components of Fe toxicity tolerance. Similar pooled-trait M-QTL approaches have been applied in other crops for complex stress or performance phenotypes, with explicit recognition that constituent traits may be governed by pleiotropic, linked, or stage-specific loci^84–86^. Consistent with this framework, post hoc dissection revealed substantial heterogeneity in M-QTL trait composition and biological integration. Several M-QTLs with large cluster sizes (e.g. 1.6, 2.2, 2.6, 3.1, 7.1–7.4, and 9.6) integrated multiple, mechanistically interrelated trait groups—including iron accumulation, redox and oxidative stress regulation, photosynthetic efficiency, and biomass or growth traits—representing integrative tolerance hubs, whereas others (e.g. 4.3, 5.1, 6.1, 8.1, and 12.1) were dominated by narrower trait categories, indicating trait-specific or context-dependent genetic control. Accordingly, the identified M-QTLs are best interpreted as integrative, trait-weighted genomic regions rather than uniform tolerance loci, and trait-resolved M-QTL analyses remain an important avenue for future refinement.

A total of 85 stable M-QTLs were identified across all 12 chromosomes. The recurrence of QTLs across independent studies highlight their genetic stability and suitability for marker-assisted breeding^86,87^. Among the 85 M-QTLs, 63 were further shortlisted based on a minimum cluster size of two. These M-QTLs exhibited varied distribution across the rice chromosomes, with chromosome 7 harbouring the highest number (9), followed by chromosomes 1 and 3. Such variability may reflect differences in gene density, recombination rates, or chromatin structure, and may also indicate the presence of QTL hotspots enriched with stress-responsive or pleiotropic loci. Similar patterns have been reported previously^41,87^. Notably, some M-QTLs, such as M-QTL 3.9, contained up to 16 CGs, whereas others, like M-QTL 3.7, explained as much as ~ 31% of the phenotypic variance. These R^2^ values reflect the quantitative and polygenic nature of Fe toxicity tolerance in rice, with moderate variance explained, likely influenced by both genetic complexity and environmental effects. The present M-QTL analysis necessarily relies on harmonisation strategies commonly adopted in meta-analyses of heterogeneous QTL and GWAS datasets. Such approaches—including the use of default LOD and R^2^ values and average physical-to-genetic distance conversions—are essential for consensus mapping but inevitably introduce uncertainty in M-QTL weighting, confidence interval length, and estimates of explained variance. This limitation is particularly relevant in rice, where recombination rates vary markedly along chromosomes, such that any single kb cM^−1^ conversion represents an approximation rather than a precise local measure [^88^; IRGSP, 2005]. Orjuela et al. (2010)^44^ addressed this issue by developing a Universal Core Genetic Map for rice based on physically anchored markers and demonstrated that a genome-wide mean conversion of ~ 200 kb cM^−1^ yields strong collinearity between projected genetic and physical maps across multiple interspecific populations, supporting the robustness of such approximations for genome-scale integration. Importantly, sensitivity analysis in the present study indicates that variation in assumed LOD thresholds, R^2^ values, and physical-to-genetic distance conversion does not alter the core biological interpretation of the results. While these assumptions can influence M-QTL weighting and confidence interval length and occasionally lead to minor positional shifts or changes in M-QTL detection, the principal M-QTL peak positions and hotspot regions remained stable across biologically plausible scenarios. Accordingly, M-QTL R^2^ values should be interpreted as approximate indicators of relative effect size, whereas M-QTL peak positions and recurrent hotspot regions represent credible genomic features supported across a wide range of reasonable analytical assumptions.

In this analysis, the mean CI of the M-QTLs was reduced from 4.64 cM to 2.12 cM, highlighting the precision achieved through M-QTL analysis, consistent with earlier reports in cereals^41,87,89–91^. A positive correlation between CI length and the number of underlying CGs was observed, aligning with previous studies^89,91^. Several M-QTLs, including 1.6, 1.8, 2.2, 2.7, 3.1, 3.2, 3.8, 3.9, 6.1, 9.5, 9.6, 9.7, and 11.1, exhibited higher CG density despite shorter CIs, likely due to larger cluster sizes or higher R^2^ values.

To evaluate robustness of identified M-QTLs, stringent thresholds were applied: cluster size ≥ 5, R^2^ ≥10%, and ≥ 5 nested CGs out of 284. Ten M-QTLs met all criteria: 1.6, 2.2, 2.6, 3.1, 6.2, 7.1, 7.2, 7.3, 7.4, and 9.6. Notably, several of these overlapped with significant MTAs, haplotype-tagging SNPs, and PPI hubs, reinforcing their biological relevance. For instance, M-QTL1.6 integrates MTA1, MTA2, and Hap-SNP1; M-QTL2.6 includes MTA6; M-QTL3.1 features MTA8 and a PPI hub; while M-QTL7.3 and M-QTL9.6 encompass MTA17 and MTA24 along with PPI hubs, respectively. Such multi-layered convergence aligns with previous GWAS in rice, emphasising the importance of integrating QTL, haplotype, and expression data to identify robust loci for Fe toxicity tolerance^10,32,61,92^.

Despite smaller cluster sizes, a few M-QTLs exhibited relatively high R^2^ values, likely due to nested CGs contributing strongly to Fe toxicity tolerance. For example, M-QTL7.6, with a cluster size of 6 and harboring a single CG (*Os07g0443500*, encoding a MYB transcription factor), recorded an R^2^ of 20%. Similar trends were observed for M-QTL1.8 and M-QTL5.3. Furthermore, chromosome 7 harbored four prioritised M-QTLs (7.1, 7.2, 7.3, 7.4), consistent with previous reports suggesting enrichment of stress-responsive loci in this region. Likewise, Devanna et al. (2024)^39^ reported that chromosomes 1 and 11 contain the highest densities of QTLs and M-QTLs associated with rice blast resistance, suggesting that certain chromosomal regions may serve as key reservoirs of stress-responsive loci across diverse biotic and abiotic stresses.

Notably, the designation of ‘high-confidence’ M-QTLs and CGs in this study reflects a prioritisation strategy rather than an absolute measure of biological importance, consistent with the polygenic nature of Fe toxicity tolerance. Thresholds based on cluster size, estimated R^2^, and the number of nested CGs were applied to highlight regions most consistently supported across independent studies, following general M-QTL practices^86,87^. M-QTLs not meeting these criteria may still contribute to Fe tolerance in a trait- or context-specific manner. Likewise, requiring differential expression in at least two transcriptome datasets favours robustness but may exclude transient or condition-specific regulators. Accordingly, both prioritised and non-prioritised M-QTLs and CGs constitute complementary resources for downstream functional validation .

Recent transcriptomic meta-analyses of Fe excess stress in rice have identified conserved molecular modules involved in metal transport, vacuolar sequestration, and oxidative stress regulation^93,94^. Several functional categories emphasised in these studies including NRAMP-type transporters, vacuolar iron transporter-like (VTL) proteins, ferritins, and stress-responsive transcription factors—are also represented among the CGs underlying key M-QTL regions identified here. Notably, several of the RNA-seq datasets used in these transcriptomic meta-analyses were also integrated in the present study; however, differences in gene prioritisation arise from distinct analytical objectives and filtering criteria. While transcriptome-based approaches prioritised globally consistent expression signatures, our framework leveraged expression recurrence across at least two studies specifically to refine CGs within genetically stable M-QTL intervals, thereby emphasising genetic localisation, heritable variation, and breeding relevance under Fe-toxic conditions.

However, it is important to mention that applying a ≥ 2 RNA-seq dataset criterion represents a pragmatic balance between inclusivity and robustness. While some context-specific regulators detected in single studies may be excluded, this filter limits noise and prioritises genes with reproducible transcriptional responses to Fe toxicity across independent experiments.

### Regulatory mechanisms governing growth and detoxification responses to Fe toxicity in rice

Fe toxicity tolerance in plants generally involves three key strategies: (i) Fe^2^⁺ exclusion at the root level (Type I), (ii) internal detoxification *via* redistribution and vacuolar storage (Type II), and (iii) ROS detoxification through antioxidant systems involving ascorbate and glutathione (Type III)^26,95^. Root-associated defence mechanisms include Fe^3+^ plaque formation, enhanced aerenchyma development, and apoplastic Fe sequestration^20^. In contrast, shoot-based mechanisms primarily involve intracellular Fe compartmentalisation and enzymatic detoxification through superoxide dismutase (SOD) and ascorbate peroxidase (APX)^24,25,96^. Collectively, these strategies mitigate Fe-induced oxidative damage and help maintain root and shoot growth under toxic Fe^2^⁺ levels.

A range of CGs identified in this study, such as *OsNRAMP6*, *OsPDR8–10*, *OsPT2*, *OsMST4*, *OsNPF2.4*, *OsSPX-MFS1*, *OsMATE35*, *OsFRDL2*, *OsPUP2/3*, *OsPILS1*, *OsZIP8*, *OsFRO2*, and *OsTIP2;2*, are predicted to encode membrane-localised or vacuolar proteins with 6–12 transmembrane domains. Their differential expression across multiple studies under Fe toxicity highlights their potential roles in Fe uptake, transport, and detoxification. For instance, *OsFRDL1* and its homolog *OsFRDL2* are implicated in reducing Fe translocation to sensitive tissues and thereby protecting shoot growth and photosynthetic function^97,98^. *OsNRAMP6*, regulated by *OsVIT1*, mediates Fe sequestration within vacuoles and chloroplasts^99,100^. Plastid-localised ferric reductases OsFRO1 and OsFRO2 are upregulated in response to Fe stress, with recent studies confirming OsFRO2 as functionally active^101^.

Root-based adaptive responses include cell wall modifications that restrict Fe^2^⁺ entry via polysaccharide-mediated chelation and associated inhibition of root elongation^102^. CGs such as *Os07g0201300* and *Os07g0241500* (UDP-glucosyltransferases) and *Os03g0808100* (cellulose biosynthesis) may act with the STAR1–STAR2 complex to export UDP-glucose, altering wall composition. Lignin deposition, involving *Os03g0809000*, *Os10g0333700*, and *Os10g0335000* (dirigent-like proteins), reinforces cortical and xylem tissues under Fe stress^53,103^. Promoter analysis of CGs revealed a high frequency of ABRE elements, highlighting ABA signalling in Fe toxicity tolerance^104^. Other stress-responsive cis-elements, including ERE, MYB, MYC, W-box, and DRE, indicating that ABA, ethylene, and stress-responsive TFs regulate Fe-induced changes in root and shoot growth and detoxification^81,105^.

Targeted association studies of 284 pre-identified CGs provide an efficient approach to dissect Fe toxicity tolerance in rice. By focusing on biologically relevant SNPs, this method narrows the genomic space, reduces multiple-testing burden, and enhances detection of meaningful associations. Similar CG-based approaches in wheat^106^ and rice traits such as grain size, salinity, and submergence^107,108^ have successfully revealed genes related to ROS regulation and ion homeostasis^60^. While this strategy improves resolution, it may miss novel loci outside predefined regions or those affected by genotype–environment interactions, thus complementing genome-wide scans and M-QTL analyses. Thus, CG-based association effectively connects regulatory polymorphisms within these genes to phenotypic variation in growth-related Fe tolerance traits.

In this study, each of the nine haplotypes (H001–H009) captured distinct allele combinations across five SNPs in 49 genotypes. Haplotype-based analysis provides higher resolution than single-marker approaches by integrating cumulative effects of multiple alleles and has been effective in identifying superior stress-tolerance alleles^84,109,110^. H004 conferred the highest Fe toxicity tolerance but was rare (< 1%, two accessions), making it a valuable donor for targeted introgression. H002, present in nine genotypes, exhibited consistent performance and is a practical breeding target. In contrast, H001, the most common haplotype (26 accessions), showed moderate tolerance, likely reflecting an ancestral variant maintained through selection. Minor haplotypes (H003, H005–H009) were infrequent and exhibited intermediate or variable responses.

Haplotype analysis of five SNPs revealed allelic variants linked to Fe tolerance. Notably, MTAs near stress-responsive kinases like *OsRPK1* highlight the role of signalling in adaptation. *OsRPK1* encodes an LRR-RLK induced by auxin, ABA, cold, and drought and acts as a negative regulator of root growth^111^. Its association with improved SH and RL under Fe stress suggests that reduced *OsRPK1* activity may enhance tolerance. The SNP identified in *OsFRDL2*, a MATE transporter, highlights its role in Fe detoxification via citrate-mediated Fe^2^⁺/Fe^3+^ chelation and sequestration. Previously characterised for Al tolerance via ART1-mediated citrate secretion^97^, its induction under Fe toxicity^20,53,54^ suggests a similar protective mechanism. Other notable loci include *Os08g0148600* (Dicer-like endoribonuclease) and *Os10g0329400* (hypothetical protein), potentially involved in regulatory or protective functions.

Although haplotype detection was limited by heterozygosity and missing data, CG-based association studies provided valuable insights into Fe tolerance genetics. For example, *OsWRKY66* mediates ROS defence^112^; *OsGLYI6* maintains redox homeostasis and enhances yield under stress^113^; and Os3BGlu6 contributes to ABA-mediated ROS reduction and drought tolerance^114^, linking hormonal response to Fe tolerance. PPI analysis further identified interaction clusters indicative of coordinated stress-response pathways, including MTA4 (*Os02g0313900*), MTA8 (*Os3BGlu6*), MTA16 (*OsFbox298*), MTA19 (*Os08g0148600*), and MTA24 (*OsCCR1*). Functionally uncharacterized proteins, such as F-box proteins and Dicer-like endoribonucleases, may have regulatory roles warranting further study.

The present study demonstrates that integrative M-QTL analysis enables refined identification of consensus genomic regions, facilitating CG discovery for complex traits like Fe toxicity tolerance. Integration with expression data enhances candidate validation, while targeted association study prioritises functionally relevant loci, detects moderate-effect genes often missed in genome-wide scans, and refines broad QTL intervals. Collectively, M-QTL mapping, CG-based AS, and haplotype analysis form a potential framework to guide breeding and validation efforts. AS identifies significant MTAs, while haplotyping refines them into multi-allelic combinations, capturing functional variation, reducing false positives, and improving the likelihood of tagging causal alleles^115^. Haplotypes H002 and H004 are especially promising: H002 balances performance and frequency, while the rare H004 exhibits maximal trait expression, making both suitable for MAS or genomic selection following confirmation in independent genetic backgrounds. These can be introgressed into elite cultivars via QTL pyramiding with minimal linkage drag^116^. Inter-subpopulation crosses (e.g., *japonica* × *indica*) may help bridge haplotype gaps. Genes such as *OsFRDL2* and other uncharacterized CGs may aid Fe detoxification. In the future, CRISPR-based validation is essential for confirming gene function and enabling precision breeding. Thus, by pinpointing regulatory alleles and haplotypes that modulate Fe detoxification and associated growth responses, this integrative framework bridges genomic variation with physiologically meaningful tolerance mechanisms.

## Conclusion

In this study, we identified 63 M-QTLs associated with Fe toxicity–related traits integrated across multiple phenotypic categories in rice, each supported by multiple independent QTLs and explaining at least 7% of the phenotypic variance. From these, 10 high-confidence M-QTLs (1.6, 2.2, 2.6, 3.1, 6.2, 7.1, 7.2, 7.3, 7.4, and 9.6) were prioritized based on their clustering patterns, with an average of 6.7 overlapping QTLs, a mean R^2^ of 11.97%, and an average of 6.6 nested CGs per region, reflecting high-confidence candidate genomic regions contributing to Fe toxicity tolerance across diverse genetic backgrounds. Additional regions, including M-QTLs 3.7, 1.7, 7.6, and 1.5, also displayed favorable genetic architecture. The enriched M-QTL intervals encompass genes involved in essential functions such as organic acid transport, metal exclusion and sequestration, antioxidant defense, hormone-mediated signaling, cell wall biosynthesis, and root system modification, core mechanisms underlying Fe toxicity tolerance. Functional relevance was further supported by in-silico validations, including promoter motif analysis, transcription factor binding predictions, and subcellular localization studies. Many M-QTLs co-localized with well-characterized CGs identified through targeted association studies, supporting their prioritisation for downstream validation and breeding-oriented research, while integration with expression datasets validated CG function and CG-based association studies effectively captured moderate-effect loci, such as *OsGLYI6* and *Os3BGlu6*, often missed in genome-wide scans. Haplotype analysis identified H002, with moderate trait expression and broad distribution, and H004, a rare but highly favorable haplotype, as promising candidates for marker-assisted and genomic selection, enabling introgression into elite cultivars via QTL pyramiding with minimal linkage drag, while inter-subpopulation crosses could bridge allelic gaps and enhance adaptability. Genes such as *OsFRDL2*, previously implicated in Al-induced citrate efflux, and other uncharacterized CGs within the M-QTLs may play analogous roles in Fe detoxification, deserving further functional validation. Collectively, the present study demonstrates that M-QTL analysis, when integrated with transcriptomic evidence and CG–based association mapping, enables the prioritisation of high-confidence genomic regions, candidate genes, and haplotypes associated with Fe toxicity tolerance in rice. These findings provide a structured, hypothesis-generating resource for downstream functional validation and breeding-oriented studies rather than definitive causal inference.

## Supplementary Information

Below is the link to the electronic supplementary material.

Supplementary Material 1

Supplementary Material 2

Supplementary Material 3

Supplementary Material 4

Supplementary Material 5

Supplementary Material 6

Supplementary Material 7

Supplementary Material 8

Supplementary Material 9

Supplementary Material 10

Supplementary Material 11

Supplementary Material 12

Supplementary Material 13

Supplementary Material 14

## Data Availability

The authors confirm that all data supporting the findings of this study are included within the article and/or its supplementary materials. The datasets generated during and/or analysed during the current study are available from the corresponding author on reasonable request.
