# Supplementary material for: Genomic dissection of iron toxicity tolerance in rice identifies key loci, candidate genes, and associated haplotypes
Source: Sci Rep. 2026 Mar 9;16:12767. doi: 10.1038/s41598-026-38841-9 (PMC13096636; doi:10.1038/s41598-026-38841-9)
Supplement: Supplementary file 1 — Supplementary Material 1 [file 41598_2026_38841_MOESM1_ESM.docx]

**Table: CGs with one or more transmembrane (TM) domains**

| **Gene ID** | **Localization** | **Encoded Gene / Description** | **TM Domains** |
| --- | --- | --- | --- |
| Os01g0503400 | Cell membrane; Lysosome/Vacuole | NRAMP6 – Iron and manganese transporter | 12 |
| Os01g0609000 | Cell membrane | PDR10 – Pleiotropic drug resistance ABC transporter | 12 |
| Os01g0609300 | Cell membrane | PDR9 – Pleiotropic drug resistance ABC transporter | 12 |
| Os01g0609900 | Cell membrane | PDR8 – Pleiotropic drug resistance ABC transporter | 12 |
| Os02g0689900 | Cell membrane | NRT1.5A – Low-affinity nitrate transporter | 12 |
| Os03g0150800 | Cell membrane | PT2 – Phosphate transporter | 12 |
| Os03g0218400 | Cell membrane | MST4 – Monosaccharide transporter | 12 |
| Os03g0571900 | Cell membrane; Lysosome/Vacuole | PEZ1 – Phenolics efflux transporter (MATE14) | 12 |
| Os03g0687000 | Cell membrane | NPF2.4 – Nitrate/peptide transporter | 12 |
| Os04g0573000 | Cell membrane; Lysosome/Vacuole | SPX-MFS1 – Phosphate transporter | 12 |
| Os08g0410500 | Cell membrane; Lysosome/Vacuole | PTR – Peptide transporter | 12 |
| Os09g0468000 | Cell membrane; Lysosome/Vacuole | MATE35 – Multidrug and toxic compound extrusion | 12 |
| Os10g0206800 | Cell membrane | FRDL2 – Citrate efflux transporter | 12 |
| Os11g0283500 | Cell membrane; Lysosome/Vacuole | PTR – TGF-beta receptor-like transporter | 12 |
| Os03g0215000 | Golgi apparatus | Integral membrane protein | 10 |
| Os05g0299500 | Golgi apparatus | DUF914 family protein | 10 |
| Os05g0444300 | Golgi apparatus | DUF914 family protein | 10 |
| Os09g0467300 | Lysosome/Vacuole | PUP2 – Purine permease | 10 |
| Os09g0467400 | Cell membrane | PUP3 – Purine permease | 10 |
| Os09g0513200 | Golgi apparatus | Solute carrier family 35 member F1 | 10 |
| Os03g0805400 | Endoplasmic reticulum | Phosphatidic acid phosphatase type 2 protein | 9 |
| Os03g0808100 | Cell membrane; ER; Golgi | CESA2 – Cellulose synthase A2 | 9 |
| Os09g0491740 | Cell membrane; ER | PILS1 – Auxin efflux carrier | 9 |
| Os07g0232800 | Cell membrane; Lysosome/Vacuole | ZIP8 – Zinc transporter | 8 |
| Os01g0266800 | Lysosome/Vacuole | Cystinosin – ERS1p repeat protein | 7 |
| Os01g0606000 | Cell membrane; Lysosome/Vacuole | SWEET6A – Sugar transporter | 7 |
| Os03g0156700 | Plastid | ZNL – Zebra-necrosis-like nickel/cobalt transporter | 7 |
| Os05g0485300 | Endoplasmic reticulum | TRAM/LAG1/CLN8 domain protein | 7 |
| Os01g0501700 | Plastid | TATC – Twin-arginine translocase subunit C | 6 |
| Os02g0621300 | Endoplasmic reticulum | CER1 – VLC alkane biosynthesis enzyme | 6 |
| Os04g0578600 | Cell membrane | FRO2 – Ferric reductase oxidase | 6 |
| Os06g0336200 | Lysosome/Vacuole | TIP2;2 – Tonoplast intrinsic protein | 6 |
| Os02g0265900 | Endoplasmic reticulum | Reticulon family protein | 4 |
| Os03g0741600 | Endoplasmic reticulum | PRA1 – Prenylated Rab acceptor protein | 4 |
| Os07g0490500 | Cell membrane | NAT – Nucleobase-ascorbate transporter | 4 |
| Os01g0393400 | Cell membrane | MDR-like ABC transporter | 2 |
| Os02g0697600 | Mitochondrion | AAA-ATPase4 | 2 |
| Os05g0169700 | Cell membrane | Sugar transporter family protein | 2 |
| Os05g0401200 | Plastid | Predicted membrane protein | 2 |
| Os05g0490900 | Plastid | Conserved hypothetical membrane protein | 2 |
| Os07g0207100 | Cell membrane | Protein kinase | 2 |
| Os11g0526200 | Plastid | Predicted membrane protein | 2 |
| Os01g0151500 | ER; Lysosome/Vacuole; Golgi | GGT4 – Gamma-glutamyl transferase | 1 |
| Os01g0152000 | Cell membrane | LRR-receptor-like kinase | 1 |
| Os01g0498300 | ER; Golgi | XXAT1 – Xylan transferase | 1 |
| Os01g0607900 | Cell membrane | RPK1 – Receptor-like protein kinase | 1 |
| Os02g0313900 | Nucleus | Plasma membrane H+-ATPase-like | 1 |
| Os02g0549200 | Cell membrane | RLCK73 – Receptor-like cytoplasmic kinase | 1 |
| Os02g0552400 | Endoplasmic reticulum | Ubiquinol-cytochrome-c reductase-like protein | 1 |
| Os02g0695600 | Endoplasmic reticulum | Conserved hypothetical protein | 1 |
| Os03g0221700 | Cell membrane; Lysosome/Vacuole | SDRLK9 – S-domain receptor-like kinase 9 | 1 |
| Os03g0802500 | Endoplasmic reticulum | AAA-ATPase1 | 1 |
| Os04g0573900 | Endoplasmic reticulum | CYP704A3 – Cytochrome P450 704A3 | 1 |
| Os05g0166300 | Cell membrane | SDRLK35 – S-domain receptor-like kinase 35 | 1 |
| Os05g0166600 | Cell membrane | SDRLK36 – S-domain receptor-like kinase 36 | 1 |
| Os05g0199100 | Cell membrane | NDR1/HIN1-like protein 2 | 1 |
| Os05g0445100 | Endoplasmic reticulum | CYP94C4 – Cytochrome P450 94C4 | 1 |
| Os06g0163300 | Cell membrane; Lysosome/Vacuole; Golgi | Harpin-induced protein | 1 |
| Os06g0165500 | Cell membrane | SDRLK37 – S-domain receptor-like kinase 37 | 1 |
| Os06g0168500 | Cell membrane; Lysosome/Vacuole | SYP131b – Qa-SNARE | 1 |
| Os07g0192000 | Endoplasmic reticulum | AAA-type ATPase | 1 |
| Os08g0236400 | Cell membrane | SDRLP5 – S-domain receptor-like protein 5 | 1 |
| Os08g0276400 | Cell membrane; Lysosome/Vacuole | Serine/threonine protein kinase | 1 |
| Os08g0395800 | Cell membrane | DUF247 family protein | 1 |
| Os09g0468300 | Cell membrane | ATL101 – RING-type E3 ubiquitin ligase | 1 |
| Os11g0528500 | Plastid | Rubredoxin 1-like protein | 1 |
