# Supplementary material for: Genomic dissection of iron toxicity tolerance in rice identifies key loci, candidate genes, and associated haplotypes
Source: Sci Rep. 2026 Mar 9;16:12767. doi: 10.1038/s41598-026-38841-9 (PMC13096636; doi:10.1038/s41598-026-38841-9)
Supplement: Supplementary file 2 — Supplementary Material 2 [file 41598_2026_38841_MOESM2_ESM.docx]

**Table: Description of 27 MTA identified**

| **MTAs** | **SNP** | **Chr. No.** | **Position (bp)** | **Traits** | **M-QTL** | **RAP gene ID** | **Gene name** |
| --- | --- | --- | --- | --- | --- | --- | --- |
| MTA 1 | 23978701 | 1 | 23978701 | BLINK.SH_Fe | 1.6 | Os01g0607900 | OsRPK1 |
| MTA 2 | 24025790 | 1 | 24025790 | FarmCPU.SH_CK | 1.6 | Os01g0608400 |  |
| MTA 3 | 35099513 | 1 | 35099513 | BLINK.SFW_CK | 1.8 | Os01g0822200 | OsRLCK47 |
| MTA 4 | 55691712 | 2 | 12420789 | FarmCPU.SH_CK | 2.3 | Os02g0313900 |  |
| MTA 5 | 63895087 | 2 | 20624164 | FarmCPU.SFW_CK, FarmCPU.SH_CK | 2.5 | Os02g0549200 | OsRLCK73 |
| MTA 6 | 67947483 | 2 | 24676560 | FarmCPU.SH_Fe, BLINK.SH_Fe | 2.6 | Os02g0620400 |  |
| MTA 7 | 71998456 | 2 | 28727533 | FarmCPU.RSFW, BLINK.RSH | 2.7 | Os02g0698800 | OsWRKY66 |
| MTA 8 | 85090287 | 3 | 5882114 | BLINK.RL_Fe | 3.1 | Os03g0212800 | Os3BGlu6 |
| MTA 9 | 85442353 | 3 | 6234180 | FarmCPU.SFW_CK |  |  |  |
| MTA 10 | 85443226 | 3 | 6235053 | FarmCPU.SH_CK |  |  |  |
| MTA 11 | 85443240 | 3 | 6235067 | FarmCPU.RL_CK, BLINK.RL_CK |  |  |  |
| MTA 12 | 112859430 | 3 | 33651257 | FarmCPU.RSH, BLINK.RSH | 3.9 | Os03g0805766 |  |
| MTA 13 | 113014855 | 3 | 33806682 | FarmCPU.SH_CK |  |  |  |
| MTA 14 | 155427563 | 5 | 4302877 | FarmCPU.RSFW, BLINK.RSFW | 5.1 | Os05g0171900 | OsGLYI6 |
| MTA 15 | 172916433 | 5 | 21791747 | FarmCPU.SH_CK |  |  |  |
| MTA 16 | 184404130 | 6 | 3321010 | FarmCPU.SH_Fe | 6.1 | Os06g0166000 | OsFbox298 |
| MTA 17 | 217553542 | 7 | 5221635 | BLINK.RSH | 7.3 | Os07g0196300 | OsFbox348 |
| MTA 18 | 226532556 | 7 | 14200649 | FarmCPU.RSFW | 7.5 | Os07g0431160 |  |
| MTA 19 | 244820024 | 8 | 2790496 | FarmCPU.SFW_Fe, BLINK.SFW_Fe | 8.1 | Os08g0148600 |  |
| MTA 20 | 252221395 | 8 | 10191867 | BLINK.RRL, BLINK.SFW_CK, FarmCPU.SH_CK, BLINK.SH_CK | 8.4 | Os08g0267300 |  |
| MTA 21 | 261677426 | 8 | 19647898 | BLINK.SFW_CK, BLINK.SH_CK |  |  |  |
| MTA 22 | 288042831 | 9 | 17570281 | BLINK.SH_CK |  |  |  |
| MTA 23 | 289364421 | 9 | 18891871 | BLINK.SH_CK |  |  |  |
| MTA 24 | 289496048 | 9 | 19023498 | BLINK.RSFW, FarmCPU.RSH, BLINK.RSH | 9.6 | Os09g0491852 | OsCCR1 |
| MTA 25 | 301064634 | 10 | 7579364 | FarmCPU.SFW_Fe | 10.1 | Os10g0206800 | OsFRDL2 |
| MTA 26 | 302752785 | 10 | 9267515 | BLINK.SH_Fe | 10.2 | Os10g0329400 |  |
| MTA 27 | 302754564 | 10 | 9269294 | FarmCPU.SH_CK |  |  |  |
