# Supplementary figures and images for: Genomic dissection of iron toxicity tolerance in rice identifies key loci, candidate genes, and associated haplotypes
Source: Sci Rep. 2026 Mar 9;16:12767. doi: 10.1038/s41598-026-38841-9 (PMC13096636; doi:10.1038/s41598-026-38841-9)

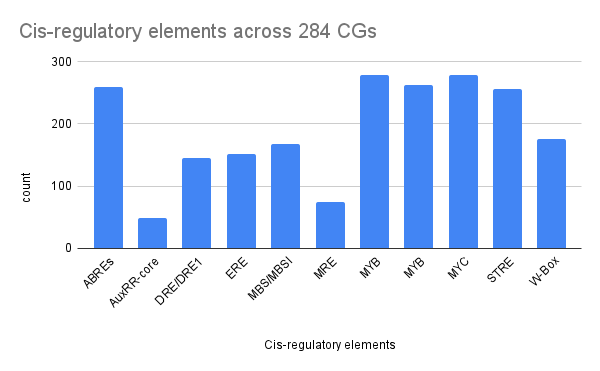

Supplement: Supplementary file 3 — Supplementary Material 3 [file 41598_2026_38841_MOESM3_ESM.png]

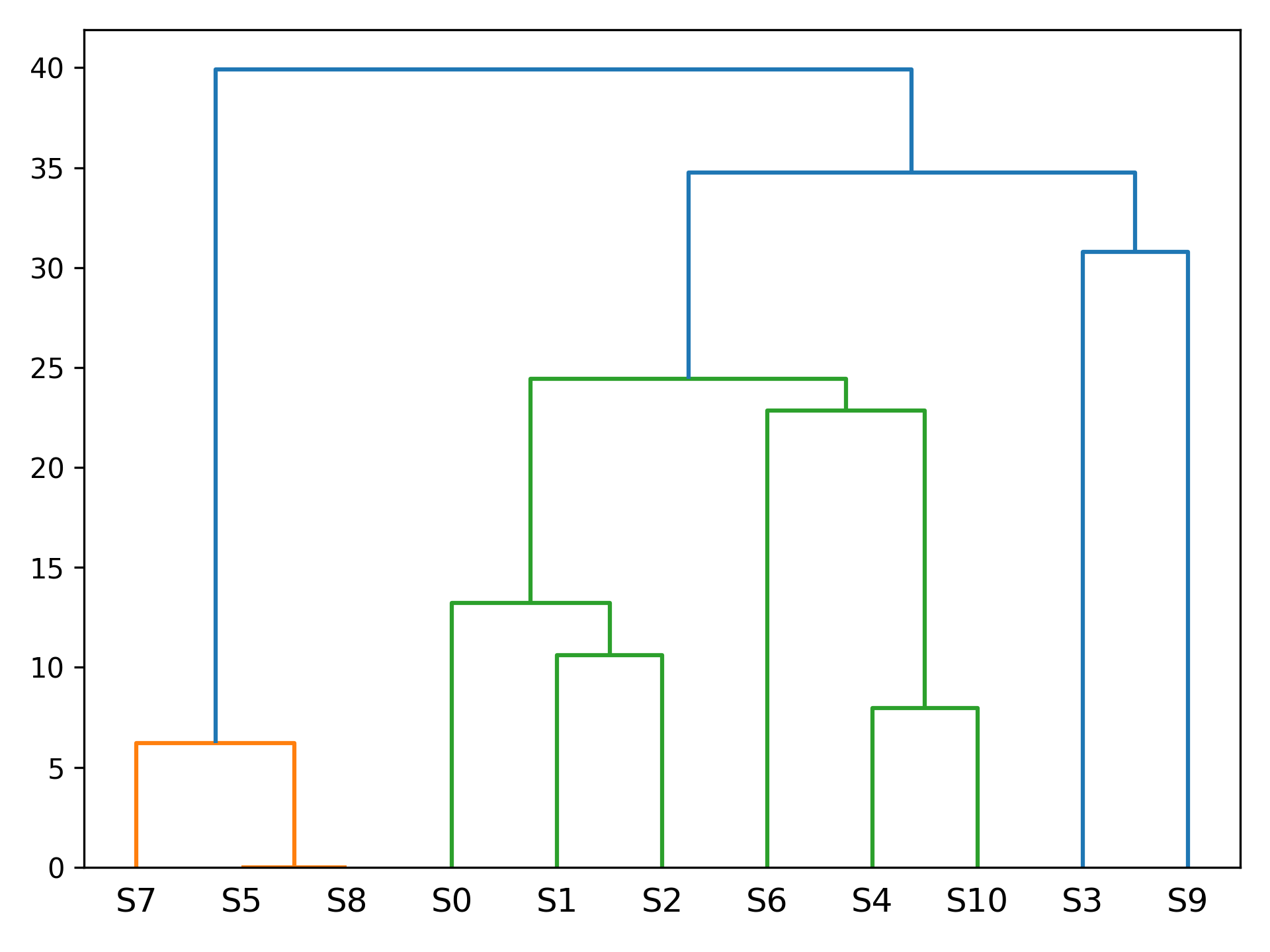

Supplement: Supplementary file 8 — Supplementary Material 8 [file 41598_2026_38841_MOESM8_ESM.png]

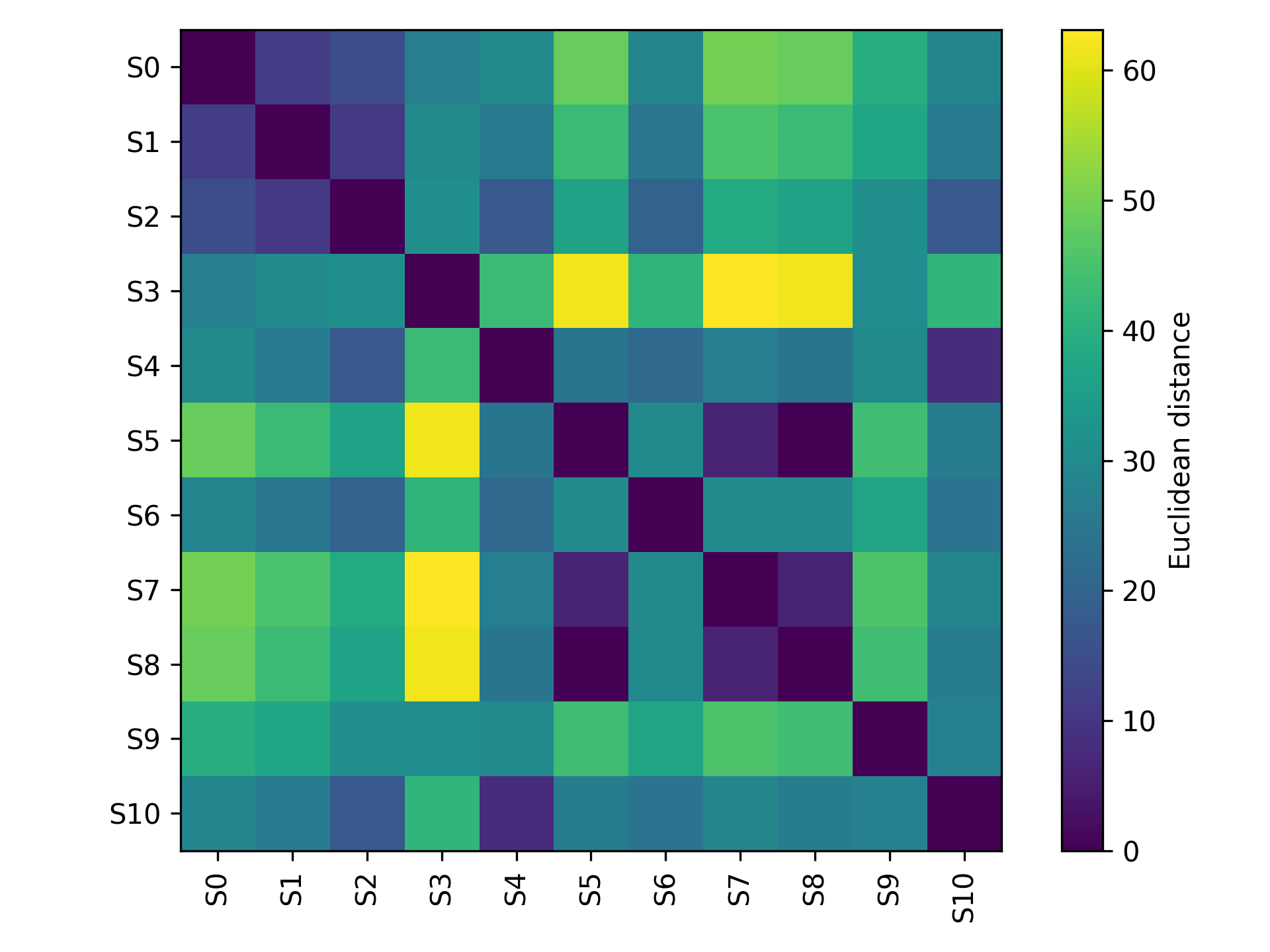

Supplement: Supplementary file 10 — Supplementary Material 10 [file 41598_2026_38841_MOESM10_ESM.png]

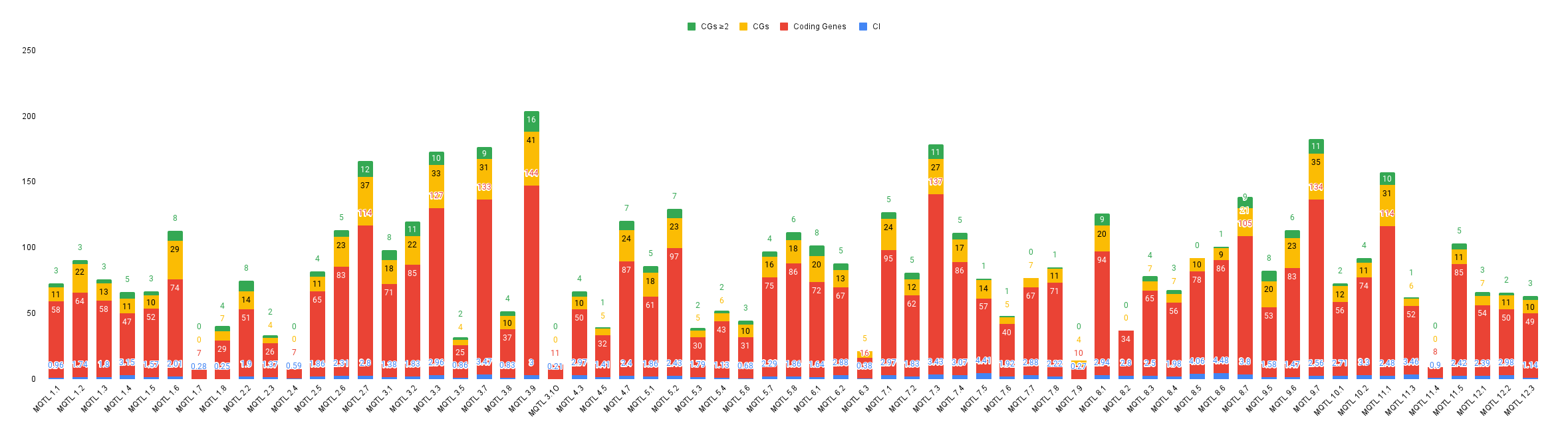

Supplement: Supplementary file 12 — Supplementary Material 12 [file 41598_2026_38841_MOESM12_ESM.png]
