# Supplementary material for: Genomic dissection of iron toxicity tolerance in rice identifies key loci, candidate genes, and associated haplotypes
Source: Sci Rep. 2026 Mar 9;16:12767. doi: 10.1038/s41598-026-38841-9 (PMC13096636; doi:10.1038/s41598-026-38841-9)
Supplement: Supplementary file 6 — Supplementary Material 6 [file 41598_2026_38841_MOESM6_ESM.docx]

**Table S2: Detail of 85 preliminary M-QTLs identified for Fe toxicity tolerance related traits**

| **MetaQTL** | **Start Marker** | **Start position (cM)** | **Stop Marker** | **Stop position (cM)** |
| --- | --- | --- | --- | --- |
| 1.1 | RM10137 | 10.87 | RM10146 | 11.83 |
| 1.2 | RM8083 | 35.57 | RM10586 | 37.31 |
| 1.3 | RM10898 | 59.63 | RM10926 | 61.43 |
| 1.4 | RM10975 | 66.81 | RM8079 | 69.96 |
| 1.5 | RM11188 | 86.29 | RM11208 | 87.86 |
| 1.6 | RM11297 | 94.94 | RM3366 | 96.95 |
| 1.7 | RM11488 | 110.14 | RM1232 | 110.42 |
| 1.8 | RM265 | 139.62 | RM3403 | 139.87 |
| 2.1 | RM12361 | 4.56 | RM12391 | 6.33 |
| 2.2 | RM7501 | 36.95 | RM12930 | 38.85 |
| 2.3 | RM5439 | 48.38 | RM13065 | 49.75 |
| 2.4 | RM13123 | 57.79 | RM13130 | 58.38 |
| 2.5 | RM2634 | 82.06 | RM13414 | 83.92 |
| 2.6 | RM13603 | 98.08 | RM13628 | 100.39 |
| 2.7 | RM525 | 113.15 | RM13843 | 115.95 |
| 2.8 | RM13970 | 126 | RM13971 | 126.31 |
| 3.1 | RM14392 | 10.94 | RM6883 | 12.32 |
| 3.2 | RM14575 | 23.33 | RM14607 | 25.16 |
| 3.3 | RM14708 | 35.19 | RM14751 | 38.15 |
| 3.4 | RM14908 | 50.4 | RM14968 | 54.4 |
| 3.5 | RM15324 | 81.73 | RM15330 | 82.59 |
| 3.6 | RM15539 | 97.57 | RM15609 | 101.85 |
| 3.7 | RM6053 | 108.75 | RM15756 | 112.22 |
| 3.8 | RM5172 | 120.94 | RM15914 | 121.77 |
| 3.9 | RM130 | 132.72 | RM16124 | 135.72 |
| 3.10 | RM3684 | 137.64 | RM16140 | 137.85 |
| 4.1 | RM16386 | 12.14 | RM16407 | 14.95 |
| 4.2 | RM16582 | 43.55 | RM16600 | 45.58 |
| 4.3 | RM16634 | 52.9 | RM16672 | 55.87 |
| 4.4 | RM16703 | 60.51 | RM16730 | 64.83 |
| 4.5 | RM16982 | 83.83 | RM6172 | 85.24 |
| 4.6 | RM17199 | 100.4 | RM17257 | 104.53 |
| 4.7 | RM17375 | 115.35 | RM17392 | 117.75 |
| 4.8 | RM17553 | 131.24 | RM17558 | 131.72 |
| 5.1 | RM437 | 15.38 | RM17980 | 17.24 |
| 5.2 | RM18069 | 24.72 | RM5994 | 27.15 |
| 5.3 | RM18142 | 30.78 | RM18166 | 32.57 |
| 5.4 | RM18318 | 52.87 | RM18343 | 54 |
| 5.5 | RM18413 | 62.44 | RM18414 | 62.57 |
| 5.6 | RM18634 | 77.6 | RM18638 | 78.28 |
| 5.7 | RM18773 | 86.08 | RM5642 | 88.37 |
| 5.8 | RM3476 | 95.01 | RM18919 | 96.87 |
| 5.9 | RM19037 | 106.36 | RM2457 | 106.64 |
| 6.1 | RM19423 | 12.22 | RM19439 | 13.86 |
| 6.2 | RM19969 | 52.19 | RM19986 | 55.07 |
| 6.3 | RM19989 | 55.15 | RM19991 | 55.53 |
| 7.1 | RM20987 | 11.32 | RM1134 | 14.29 |
| 7.2 | RM8009 | 16.71 | RM8263 | 18.54 |
| 7.3 | RM21133 | 19.71 | RM21185 | 23.14 |
| 7.4 | RM21259 | 28.68 | RM3718 | 31.75 |
| 7.5 | RM21439 | 53.3 | RM21465 | 57.71 |
| 7.6 | RM21478 | 58.52 | RM21500 | 60.44 |
| 7.7 | RM21513 | 62.35 | RM21534 | 65.23 |
| 7.8 | RM418 | 72.11 | RM6184 | 74.33 |
| 7.9 | RM21796 | 87.52 | RM5495 | 87.79 |
| Model 8.1 | RM8040 | 7.94 | RM22373 | 10.88 |
| 8.2 | RM22601 | 25.54 | RM22628 | 28.34 |
| 8.3 | RM7633 | 30.91 | RM6429 | 33.41 |
| 8.4 | RM22759 | 40.63 | RM22779 | 42.61 |
| 8.5 | RM22789 | 44.52 | RM22832 | 48.58 |
| 8.6 | RM22956 | 65.43 | RM22986 | 69.91 |
| 8.7 | RM23037 | 74.98 | RM23080 | 78.78 |
| 8.8 | RM23278 | 92.10 | RM23289 | 92.71 |
| Model9 9.1 | RM24001 | 35.81 | RM24012 | 37.05 |
| 9.2 | RM24105 | 44.49 | RM24131 | 46.22 |
| 9.3 | RM24144 | 47.21 | RM24158 | 48.41 |
| 9.4 | RM24306 | 58.45 | RM24372 | 62.52 |
| 9.5 | RM24497 | 69.36 | RM3533 | 70.94 |
| 9.6 | RM24575 | 74.51 | RM24600 | 75.98 |
| 9.7 | RM3164 | 76.03 | RM3787 | 78.59 |
| 9.8 | RM24706 | 82.58 | RM5384 | 86.67 |
| 9.9 | RM24816 | 88.32 | RM2885 | 89.22 |
| 10.1 | RM25139 | 27.95 | RM25164 | 30.66 |
| 10.2 | RM25179 | 33.36 | RM25214 | 36.66 |
| 10.3 | RM25747 | 78.62 | RM25758 | 79.18 |
| 11.1 | RM25985 | 2.64 | RM26021 | 5.12 |
| 11.2 | RM26228 | 20.86 | RM26252 | 23.01 |
| 11.3 | RM1206 | 38.43 | RM26473 | 41.89 |
| 11.4 | RM26574 | 52.02 | RM6091 | 52.92 |
| 11.5 | RM26840 | 74.78 | RM26883 | 77.2 |
| 11.6 | RM27284 | 106.57 | RM7654-2 | 106.91 |
| 12.1 | RM27735 | 23.95 | RM27777 | 26.34 |
| 12.2 | RM27948 | 44.28 | RM27957 | 47.26 |
| 12.3 | RM28482 | 92.47 | RM28501 | 93.61 |
| 12.4 | RM28667 | 101.91 | RM28668 | 102.1 |
