# Supplementary material for: Genomic dissection of iron toxicity tolerance in rice identifies key loci, candidate genes, and associated haplotypes
Source: Sci Rep. 2026 Mar 9;16:12767. doi: 10.1038/s41598-026-38841-9 (PMC13096636; doi:10.1038/s41598-026-38841-9)
Supplement: Supplementary file 7 — Supplementary Material 7 [file 41598_2026_38841_MOESM7_ESM.docx]

Table S2: Distribution of 4,070 non-redundant genes nested within 63 M-QTLs retrieved from RAP database.

| M-QTL | Coding genes (RAP Database Gene IDs) | Total count |
| --- | --- | --- |
| 1.1 | Os01g0150750, Os01g0150800, Os01g0150900, Os01g0150950, Os01g0151001, Os01g0151100, Os01g0151200, Os01g0151400, Os01g0151500, Os01g0151600, Os01g0151700, Os01g0151750, Os01g0151800, Os01g0151900, Os01g0152000, Os01g0152100, Os01g0152200, Os01g0152300, Os01g0152500, Os01g0152600, Os01g0152675, Os01g0152687, Os01g0152700, Os01g0152800, Os01g0152900, Os01g0152950, Os01g0152951, Os01g0153000, Os01g0153100, Os01g0153150, Os01g0153200, Os01g0153250, Os01g0153275, Os01g0153300, Os01g0153400, Os01g0153500, Os01g0153550, Os01g0153600, Os01g0153625, Os01g0153651, Os01g0153700, Os01g0153800, Os01g0153900, Os01g0153950, Os01g0154000, Os01g0154100, Os01g0154200, Os01g0154250, Os01g0154300, Os01g0154500, Os01g0154550, Os01g0154600, Os01g0154950, Os01g0155000, Os01g0155100, Os01g0155200, Os01g0155300, Os01g0155400 | 58 |
| 1.2 | Os01g0263300, Os01g0263400, Os01g0263500, Os01g0263600, Os01g0263900, Os01g0264000, Os01g0264001, Os01g0264100, Os01g0264125, Os01g0264150, Os01g0264200, Os01g0264400, Os01g0264500, Os01g0264600, Os01g0264700, Os01g0264900, Os01g0265000, Os01g0265100, Os01g0265200, Os01g0265400, Os01g0265600, Os01g0265700, Os01g0265800, Os01g0265900, Os01g0266000, Os01g0266100, Os01g0266200, Os01g0266400, Os01g0266500, Os01g0266600, Os01g0266800, Os01g0267000, Os01g0267050, Os01g0267100, Os01g0267200, Os01g0267300, Os01g0267400, Os01g0267600, Os01g0267800, Os01g0267900, Os01g0268000, Os01g0268100, Os01g0268201, Os01g0268300, Os01g0268600, Os01g0268700, Os01g0268733, Os01g0268766, Os01g0268800, Os01g0268900, Os01g0269000, Os01g0269100, Os01g0269301, Os01g0269500, Os01g0269550, Os01g0269600, Os01g0269700, Os01g0269800, Os01g0269900, Os01g0269950, Os01g0270000, Os01g0270100, Os01g0270300, Os01g0270501 | 64 |
| 1.3 | Os01g0365201, Os01g0365250, Os01g0365300, Os01g0366100, Os01g0366201, Os01g0366300, Os01g0366400, Os01g0366501, Os01g0367100, Os01g0367201, Os01g0367300, Os01g0367350, Os01g0367400, Os01g0367450, Os01g0367500, Os01g0367601, Os01g0367700, Os01g0367750, Os01g0367800, Os01g0367900, Os01g0368000, Os01g0368500, Os01g0368600, Os01g0368700, Os01g0368900, Os01g0369000, Os01g0369200, Os01g0369300, Os01g0369366, Os01g0369432, Os01g0369500, Os01g0369600, Os01g0369700, Os01g0369800, Os01g0369900, Os01g0369950, Os01g0370000, Os01g0370200, Os01g0370400, Os01g0370600, Os01g0370750, Os01g0370900, Os01g0371200, Os01g0371300, Os01g0371400, Os01g0371500, Os01g0372100, Os01g0372300, Os01g0372350, Os01g0372400, Os01g0372450, Os01g0372500, Os01g0372700, Os01g0372866, Os01g0373033, Os01g0373200, Os01g0373400, Os01g0373500 | 58 |
| 1.5 | Os01g0393100, Os01g0393200, Os01g0393300, Os01g0393400, Os01g0444100, Os01g0495200, Os01g0495701, Os01g0495900, Os01g0496800, Os01g0496900, Os01g0497400, Os01g0497800, Os01g0498200, Os01g0498300, Os01g0498802, Os01g0499300, Os01g0499350, Os01g0499400, Os01g0499800, Os01g0500100, Os01g0500400, Os01g0500450, Os01g0500500, Os01g0500600, Os01g0500750, Os01g0500825, Os01g0500900, Os01g0501000, Os01g0501400, Os01g0501700, Os01g0501800, Os01g0501850, Os01g0501900, Os01g0502001, Os01g0502101, Os01g0502300, Os01g0502400, Os01g0502700, Os01g0502800, Os01g0502900, Os01g0503100, Os01g0503300, Os01g0503351, Os01g0503400, Os01g0504100, Os01g0504300, Os01g0504500 | 52 |
| 1.6 | Os01g0603500, Os01g0603700, Os01g0603800, Os01g0604000, Os01g0604100, Os01g0604150, Os01g0604350, Os01g0604500, Os01g0604550, Os01g0604600, Os01g0604700, Os01g0604900, Os01g0605000, Os01g0605100, Os01g0605300, Os01g0605400, Os01g0605500, Os01g0605600, Os01g0605650, Os01g0605700, Os01g0605850, Os01g0606000, Os01g0606050, Os01g0606100, Os01g0606133, Os01g0606166, Os01g0606200, Os01g0606400, Os01g0606500, Os01g0606900, Os01g0607000, Os01g0607050, Os01g0607100, Os01g0607200, Os01g0607250, Os01g0607300, Os01g0607400, Os01g0607600, Os01g0607800, Os01g0607900, Os01g0608000, Os01g0608101, Os01g0608200, Os01g0608300, Os01g0608400, Os01g0608700, Os01g0608800, Os01g0608900, Os01g0609000, Os01g0609200, Os01g0609300, Os01g0609501, Os01g0609700, Os01g0609900, Os01g0610000, Os01g0610050, Os01g0610100, Os01g0610300, Os01g0610400, Os01g0610500, Os01g0610600, Os01g0610700, Os01g0610800, Os01g0611000, Os01g0611050, Os01g0611100, Os01g0611200, Os01g0611300, Os01g0611700, Os01g0611800, Os01g0611900, Os01g0611950, Os01g0612000, Os01g0612200 | 74 |
| 1.7 | Os01g0672166, Os01g0672201, Os01g0672300, Os01g0672400, Os01g0672500, Os01g0672600, Os01g0672700 | 7 |
| 1.8 | Os01g0820700, Os01g0820750, Os01g0820800, Os01g0820900, Os01g0821200, Os01g0821300, Os01g0821600, Os01g0821700, Os01g0821800, Os01g0821900, Os01g0822001, Os01g0822100, Os01g0822200, Os01g0822501, Os01g0822800, Os01g0822900, Os01g0823100, Os01g0823150, Os01g0823200, Os01g0823300, Os01g0823400, Os01g0823500, Os01g0823550, Os01g0823600, Os01g0823700, Os01g0823800, Os01g0823900, Os01g0823951, Os01g0824000 | 29 |
| 2.2 | Os02g0262800, Os02g0263432, Os02g0263601, Os02g0263900, Os02g0264300, Os02g0264501, Os02g0264600, Os02g0264700, Os02g0264800, Os02g0265100, Os02g0265200, Os02g0265300, Os02g0265400, Os02g0265700, Os02g0265800, Os02g0265900, Os02g0266000, Os02g0266100, Os02g0266200, Os02g0266300, Os02g0266500, Os02g0266650, Os02g0266800, Os02g0266900, Os02g0267000, Os02g0267200, Os02g0267350, Os02g0267600, Os02g0267700, Os02g0267766, Os02g0267832, Os02g0267900, Os02g0267975, Os02g0268000, Os02g0268050, Os02g0268100, Os02g0268300, Os02g0268400, Os02g0268500, Os02g0268600, Os02g0268675, Os02g0268750, Os02g0269000, Os02g0269200, Os02g0269400, Os02g0269600, Os02g0269625, Os02g0269650, Os02g0270100, Os02g0270200, Os02g0270350 | 51 |
| 2.3 | Os02g0308300, Os02g0308400, Os02g0308800, Os02g0309200, Os02g0309500, Os02g0310200, Os02g0310400, Os02g0310500, Os02g0310825, Os02g0311150, Os02g0311600, Os02g0311800, Os02g0312300, Os02g0312500, Os02g0312550, Os02g0312600, Os02g0312700, Os02g0312750, Os02g0312800, Os02g0313051, Os02g0313300, Os02g0313400, Os02g0313450, Os02g0313500, Os02g0313700, Os02g0313900 | 26 |
| 2.4 | Os02g0445100, Os02g0445200, Os02g0445300, Os02g0445600, Os02g0445700, Os02g0446100, Os02g0447050 | 7 |
| 2.5 | Os02g0547300, Os02g0547600, Os02g0547900, Os02g0548100, Os02g0548500, Os02g0548700, Os02g0548801, Os02g0548900, Os02g0549200, Os02g0549401, Os02g0549467, Os02g0549533, Os02g0549600, Os02g0549650, Os02g0549700, Os02g0549800, Os02g0549850, Os02g0549900, Os02g0550000, Os02g0550050, Os02g0550100, Os02g0550300, Os02g0550400, Os02g0550500, Os02g0550566, Os02g0550600, Os02g0550650, Os02g0550700, Os02g0550800, Os02g0550850, Os02g0550900, Os02g0551000, Os02g0551100, Os02g0551200, Os02g0551300, Os02g0551400, Os02g0551600, Os02g0551700, Os02g0551801, Os02g0551900, Os02g0552000, Os02g0552100, Os02g0552150, Os02g0552200, Os02g0552400, Os02g0552450, Os02g0552500, Os02g0552550, Os02g0552600, Os02g0552700, Os02g0553000, Os02g0553200, Os02g0553400, Os02g0553500, Os02g0553600, Os02g0553801, Os02g0554000, Os02g0554100, Os02g0554200, Os02g0554300, Os02g0554400, Os02g0554500, Os02g0554650, Os02g0554725, Os02g0554800 | 65 |
| 2.6 | Os02g0617400, Os02g0617500, Os02g0617550, Os02g0617600, Os02g0617700, Os02g0617800, Os02g0617900, Os02g0618000, Os02g0618100, Os02g0618200, Os02g0618250, Os02g0618301, Os02g0618400, Os02g0618525, Os02g0618650, Os02g0618700, Os02g0618801, Os02g0618900, Os02g0619000, Os02g0619100, Os02g0619166, Os02g0619200, Os02g0619300, Os02g0619500, Os02g0619600, Os02g0619801, Os02g0620000, Os02g0620100, Os02g0620200, Os02g0620400, Os02g0620500, Os02g0620533, Os02g0620566, Os02g0620600, Os02g0620800, Os02g0620900, Os02g0621100, Os02g0621300, Os02g0621350, Os02g0621400, Os02g0621500, Os02g0621600, Os02g0621700, Os02g0621800, Os02g0621850, Os02g0622100, Os02g0622150, Os02g0622200, Os02g0622300, Os02g0622350, Os02g0622400, Os02g0622500, Os02g0622900, Os02g0623000, Os02g0623300, Os02g0623400, Os02g0623500, Os02g0623550, Os02g0623600, Os02g0623766, Os02g0623932, Os02g0624100, Os02g0624300, Os02g0624350, Os02g0624400, Os02g0624700, Os02g0625000, Os02g0625050, Os02g0625100, Os02g0625300, Os02g0625500, Os02g0625900, Os02g0626000, Os02g0626100, Os02g0626200, Os02g0626400, Os02g0626532, Os02g0626600, Os02g0627100, Os02g0627700, Os02g0627750, Os02g0627800, Os02g0627900 | 83 |
| 2.7 | Os02g0689500, Os02g0689550, Os02g0689600, Os02g0689700, Os02g0689800, Os02g0689900, Os02g0690000, Os02g0690200, Os02g0690300, Os02g0690500, Os02g0690550, Os02g0690600, Os02g0690700, Os02g0690800, Os02g0691500, Os02g0691650, Os02g0691800, Os02g0691900, Os02g0692000, Os02g0692700, Os02g0692800, Os02g0692901, Os02g0693000, Os02g0693200, Os02g0693400, Os02g0693450, Os02g0693500, Os02g0693601, Os02g0693650, Os02g0693700, Os02g0693901, Os02g0694100, Os02g0694201, Os02g0694300, Os02g0694400, Os02g0694600, Os02g0694700, Os02g0694800, Os02g0694900, Os02g0695050, Os02g0695200, Os02g0695500, Os02g0695550, Os02g0695600, Os02g0695700, Os02g0695800, Os02g0695900, Os02g0696200, Os02g0696500, Os02g0696600, Os02g0696650, Os02g0696700, Os02g0696900, Os02g0697100, Os02g0697200, Os02g0697300, Os02g0697400, Os02g0697450, Os02g0697500, Os02g0697550, Os02g0697600, Os02g0697650, Os02g0697700, Os02g0697800, Os02g0697900, Os02g0698000, Os02g0698100, Os02g0698200, Os02g0698800, Os02g0698901, Os02g0699000, Os02g0699150, Os02g0699300, Os02g0699350, Os02g0699400, Os02g0699433, Os02g0699466, Os02g0699500, Os02g0699600, Os02g0699700, Os02g0699800, Os02g0699812, Os02g0699824, Os02g0699836, Os02g0699850, Os02g0699900, Os02g0700000, Os02g0700100, Os02g0700300, Os02g0700350, Os02g0700400, Os02g0700500, Os02g0700600, Os02g0700650, Os02g0700700, Os02g0700901, Os02g0701100, Os02g0701300, Os02g0701600, Os02g0701700, Os02g0701750, Os02g0701800, Os02g0701900, Os02g0702000, Os02g0702100, Os02g0702300, Os02g0702351, Os02g0702400, Os02g0702500, Os02g0702550, Os02g0702600, Os02g0702633, Os02g0702666, Os02g0702700 | 114 |
| 3.1 | Os03g0149400, Os03g0149700, Os03g0149750, Os03g0149800, Os03g0150000, Os03g0150101, Os03g0150200, Os03g0150500, Os03g0150550, Os03g0150600, Os03g0150666, Os03g0150700, Os03g0150800, Os03g0150901, Os03g0151000, Os03g0151100, Os03g0151201, Os03g0151300, Os03g0151500, Os03g0151600, Os03g0151700, Os03g0151800, Os03g0151850, Os03g0151900, Os03g0152000, Os03g0152100, Os03g0152200, Os03g0152300, Os03g0152400, Os03g0152500, Os03g0152600, Os03g0152700, Os03g0152800, Os03g0152900, Os03g0152950, Os03g0153000, Os03g0153100, Os03g0153400, Os03g0153500, Os03g0153900, Os03g0154000, Os03g0154100, Os03g0154300, Os03g0154700, Os03g0154750, Os03g0154800, Os03g0155000, Os03g0155300, Os03g0155400, Os03g0155500, Os03g0155566, Os03g0155632, Os03g0155700, Os03g0155900, Os03g0155933, Os03g0155966, Os03g0156000, Os03g0156100, Os03g0156200, Os03g0156300, Os03g0156366, Os03g0156432, Os03g0156500, Os03g0156600, Os03g0156700, Os03g0157000, Os03g0157300, Os03g0157400, Os03g0157450, Os03g0157500, Os03g0157600 | 71 |
| 3.2 | Os03g0212500, Os03g0212600, Os03g0212700, Os03g0212800, Os03g0212950, Os03g0213100, Os03g0213150, Os03g0213200, Os03g0213300, Os03g0213400, Os03g0213500, Os03g0213600, Os03g0213700, Os03g0213750, Os03g0213800, Os03g0213900, Os03g0214000, Os03g0214050, Os03g0214100, Os03g0214200, Os03g0214400, Os03g0214600, Os03g0214750, Os03g0214900, Os03g0215000, Os03g0215200, Os03g0215400, Os03g0215600, Os03g0215700, Os03g0215800, Os03g0215900, Os03g0216000, Os03g0216300, Os03g0216400, Os03g0216500, Os03g0216550, Os03g0216600, Os03g0216700, Os03g0216733, Os03g0216766, Os03g0216800, Os03g0216900, Os03g0217000, Os03g0217100, Os03g0217200, Os03g0217400, Os03g0217801, Os03g0217850, Os03g0217900, Os03g0218100, Os03g0218200, Os03g0218300, Os03g0218400, Os03g0218500, Os03g0218600, Os03g0218800, Os03g0219000, Os03g0219100, Os03g0219200, Os03g0219300, Os03g0219350, Os03g0219400, Os03g0219450, Os03g0219500, Os03g0219700, Os03g0219800, Os03g0219900, Os03g0220100, Os03g0220200, Os03g0220300, Os03g0220700, Os03g0221100, Os03g0221150, Os03g0221200, Os03g0221300, Os03g0221500, Os03g0221600, Os03g0221700, Os03g0221800, Os03g0222100, Os03g0222200, Os03g0222300, Os03g0222350, Os03g0222401, Os03g0222500 | 85 |
| 3.3 | Os03g0266800, Os03g0266900, Os03g0267000, Os03g0267100, Os03g0267200, Os03g0267300, Os03g0267400, Os03g0267450, Os03g0267500, Os03g0267550, Os03g0267600, Os03g0267700, Os03g0267800, Os03g0267850, Os03g0267901, Os03g0268000, Os03g0268050, Os03g0268100, Os03g0268200, Os03g0268250, Os03g0268300, Os03g0268400, Os03g0268450, Os03g0268501, Os03g0268600, Os03g0268750, Os03g0268900, Os03g0269000, Os03g0269100, Os03g0269300, Os03g0269500, Os03g0269700, Os03g0269800, Os03g0269900, Os03g0269950, Os03g0270000, Os03g0270200, Os03g0270300, Os03g0270400, Os03g0270500, Os03g0270600, Os03g0270700, Os03g0270800, Os03g0270900, Os03g0271100, Os03g0271200, Os03g0271300, Os03g0271350, Os03g0271400, Os03g0271500, Os03g0271600, Os03g0271800, Os03g0271900, Os03g0272200, Os03g0272300, Os03g0272400, Os03g0272650, Os03g0272900, Os03g0273200, Os03g0273800, Os03g0273900, Os03g0274000, Os03g0274300, Os03g0274350, Os03g0274400, Os03g0274700, Os03g0274800, Os03g0274950, Os03g0275100, Os03g0275300, Os03g0275350, Os03g0275400, Os03g0275450, Os03g0275500, Os03g0275600, Os03g0275700, Os03g0275750, Os03g0275800, Os03g0275900, Os03g0275950, Os03g0276000, Os03g0276300, Os03g0276400, Os03g0276500, Os03g0276600, Os03g0276700, Os03g0276800, Os03g0276850, Os03g0276900, Os03g0277000, Os03g0277100, Os03g0277300, Os03g0277400, Os03g0277500, Os03g0277600, Os03g0277650, Os03g0277700, Os03g0277800, Os03g0278000, Os03g0278066, Os03g0278132, Os03g0278200, Os03g0278250, Os03g0278300, Os03g0278333, Os03g0278366, Os03g0278400, Os03g0278500, Os03g0278533, Os03g0278566, Os03g0278600, Os03g0278650, Os03g0278700, Os03g0278800, Os03g0278900, Os03g0279000, Os03g0279200, Os03g0279400, Os03g0279500, Os03g0279600, Os03g0279700, Os03g0279801, Os03g0279816, Os03g0279832, Os03g0279900, Os03g0279950, Os03g0280000 | 127 |
| 3.5 | Os03g0570100, Os03g0570200, Os03g0570300, Os03g0570458, Os03g0570616, Os03g0570775, Os03g0570800, Os03g0571250, Os03g0571700, Os03g0571800, Os03g0571900, Os03g0572050, Os03g0572083, Os03g0572116, Os03g0572150, Os03g0572250, Os03g0572300, Os03g0572800, Os03g0572900, Os03g0572925, Os03g0572950, Os03g0573001, Os03g0573117, Os03g0573233, Os03g0573350 | 25 |
| 3.7 | Os03g0686500, Os03g0686600, Os03g0686700, Os03g0686800, Os03g0686900, Os03g0687000, Os03g0687100, Os03g0687200, Os03g0687400, Os03g0687600, Os03g0687700, Os03g0687800, Os03g0687900, Os03g0688000, Os03g0688100, Os03g0688200, Os03g0688250, Os03g0688300, Os03g0688450, Os03g0688600, Os03g0689100, Os03g0689300, Os03g0689350, Os03g0689400, Os03g0689544, Os03g0689688, Os03g0689833, Os03g0689866, Os03g0689900, Os03g0690000, Os03g0690500, Os03g0690600, Os03g0690700, Os03g0691050, Os03g0691400, Os03g0691500, Os03g0691600, Os03g0691800, Os03g0691900, Os03g0692000, Os03g0692100, Os03g0692300, Os03g0692350, Os03g0692400, Os03g0692700, Os03g0692850, Os03g0693000, Os03g0693400, Os03g0693500, Os03g0693600, Os03g0693650, Os03g0693700, Os03g0693800, Os03g0693900, Os03g0694000, Os03g0694250, Os03g0694500, Os03g0694600, Os03g0694650, Os03g0694700, Os03g0694800, Os03g0694900, Os03g0695000, Os03g0695100, Os03g0695200, Os03g0695300, Os03g0695400, Os03g0695425, Os03g0695450, Os03g0695500, Os03g0695600, Os03g0695700, Os03g0696000, Os03g0696150, Os03g0696300, Os03g0696500, Os03g0696850, Os03g0697200, Os03g0697333, Os03g0697466, Os03g0697600, Os03g0697833, Os03g0698066, Os03g0698300, Os03g0698350, Os03g0698400, Os03g0698500, Os03g0698800, Os03g0698900, Os03g0699000, Os03g0699100, Os03g0699200, Os03g0699300, Os03g0699400, Os03g0699600, Os03g0699700, Os03g0699750, Os03g0699800, Os03g0699950, Os03g0700100, Os03g0700150, Os03g0700200, Os03g0700301, Os03g0700350, Os03g0700400, Os03g0700450, Os03g0700500, Os03g0700600, Os03g0700700, Os03g0700750, Os03g0700800, Os03g0701000, Os03g0701100, Os03g0701200, Os03g0701300, Os03g0701400, Os03g0701500, Os03g0701550, Os03g0701600, Os03g0701700, Os03g0701750, Os03g0701800, Os03g0701900, Os03g0702000, Os03g0702100, Os03g0702133, Os03g0702400, Os03g0702500, Os03g0702600, Os03g0702700, Os03g0702800, Os03g0702900, Os03g0703000 | 127 |
| 3.8 | Os03g0741400, Os03g0741500, Os03g0741525, Os03g0741550, Os03g0741600, Os03g0741700, Os03g0741900, Os03g0741950, Os03g0742001, Os03g0742300, Os03g0742400, Os03g0742800, Os03g0742850, Os03g0742900, Os03g0743200, Os03g0743400, Os03g0743500, Os03g0743650, Os03g0743800, Os03g0743900, Os03g0744100, Os03g0744200, Os03g0744300, Os03g0744501, Os03g0744600, Os03g0744650, Os03g0744675, Os03g0744700, Os03g0744800, Os03g0744900, Os03g0745000, Os03g0745100, Os03g0745400, Os03g0745500, Os03g0745550, Os03g0745600, Os03g0745700 | 37 |
| 3.9 | Os03g0800500, Os03g0800700, Os03g0800800, Os03g0800900, Os03g0801000, Os03g0801300, Os03g0801500, Os03g0801600, Os03g0801700, Os03g0801800, Os03g0801900, Os03g0802000, Os03g0802050, Os03g0802100, Os03g0802200, Os03g0802300, Os03g0802400, Os03g0802500, Os03g0802600, Os03g0802650, Os03g0802700, Os03g0802800, Os03g0802900, Os03g0803000, Os03g0803033, Os03g0803066, Os03g0803100, Os03g0803200, Os03g0803250, Os03g0803300, Os03g0803500, Os03g0803600, Os03g0803700, Os03g0803800, Os03g0803900, Os03g0803950, Os03g0804000, Os03g0804100, Os03g0804200, Os03g0804250, Os03g0804300, Os03g0804400, Os03g0804500, Os03g0804600, Os03g0804700, Os03g0804800, Os03g0804900, Os03g0805100, Os03g0805200, Os03g0805300, Os03g0805350, Os03g0805400, Os03g0805500, Os03g0805600, Os03g0805700, Os03g0805733, Os03g0805766, Os03g0805800, Os03g0805850, Os03g0805900, Os03g0806100, Os03g0806300, Os03g0806400, Os03g0806500, Os03g0806600, Os03g0806700, Os03g0806800, Os03g0806900, Os03g0807000, Os03g0807100, Os03g0807150, Os03g0807200, Os03g0807400, Os03g0807500, Os03g0807600, Os03g0807700, Os03g0807800, Os03g0807900, Os03g0808000, Os03g0808100, Os03g0808150, Os03g0808175, Os03g0808200, Os03g0808250, Os03g0808300, Os03g0808350, Os03g0808400, Os03g0808500, Os03g0808600, Os03g0808750, Os03g0808900, Os03g0808950, Os03g0809000, Os03g0809100, Os03g0809150, Os03g0809200, Os03g0809300, Os03g0809400, Os03g0809700, Os03g0809800, Os03g0809900, Os03g0810100, Os03g0810300, Os03g0810500, Os03g0810600, Os03g0810700, Os03g0810750, Os03g0810800, Os03g0810850, Os03g0810900, Os03g0811100, Os03g0811200, Os03g0811300, Os03g0811400, Os03g0811500, Os03g0811550, Os03g0811600, Os03g0811700, Os03g0811800, Os03g0811850, Os03g0811900, Os03g0812000, Os03g0812100, Os03g0812200, Os03g0812300, Os03g0812400, Os03g0812500, Os03g0812800, Os03g0813100, Os03g0813200, Os03g0813300, Os03g0813450, Os03g0813500, Os03g0813700, Os03g0814200, Os03g0814500, Os03g0814550, Os03g0814600, Os03g0814800, Os03g0815100, Os03g0815200, Os03g0815266, Os03g0815332, Os03g0815400 | 144 |
| 3.10* | Os03g0824300, Os03g0824350, Os03g0824400, Os03g0824500, Os03g0824600, Os03g0824650, Os03g0824700, Os03g0824800, Os03g0824900, Os03g0825000, Os03g0825300 | 11 |
| 4.3 | Os04g0298700, Os04g0298850, Os04g0299500, Os04g0300100, Os04g0300400, Os04g0300600, Os04g0300950, Os04g0301225, Os04g0301500, Os04g0301700, Os04g0302300, Os04g0302400, Os04g0302900, Os04g0303100, Os04g0303250, Os04g0303900, Os04g0303950, Os04g0304000, Os04g0304200, Os04g0304250, Os04g0304301, Os04g0304400, Os04g0304600, Os04g0304750, Os04g0304900, Os04g0305300, Os04g0305700, Os04g0306300, Os04g0306350, Os04g0306400, Os04g0306550, Os04g0306700, Os04g0306750, Os04g0306800, Os04g0307200, Os04g0307300, Os04g0307400, Os04g0307433, Os04g0307466, Os04g0307500, Os04g0307567, Os04g0307634, Os04g0307701, Os04g0307800, Os04g0307900, Os04g0308000, Os04g0308100, Os04g0308400, Os04g0308500, Os04g0308600 | 50 |
| 4.5 | Os04g0421800, Os04g0421850, Os04g0421900, Os04g0422000, Os04g0422050, Os04g0422101, Os04g0422200, Os04g0422300, Os04g0422401, Os04g0422500, Os04g0422600, Os04g0422700, Os04g0422750, Os04g0422800, Os04g0422900, Os04g0422950, Os04g0423000, Os04g0423050, Os04g0423100, Os04g0423200, Os04g0423400, Os04g0423600, Os04g0423700, Os04g0423800, Os04g0424200, Os04g0424601, Os04g0425100, Os04g0425500, Os04g0425800, Os04g0426500, Os04g0426700, Os04g0426900 | 32 |
| 4.7 | Os04g0569300, Os04g0569400, Os04g0569500, Os04g0569900, Os04g0570000, Os04g0570125, Os04g0570250, Os04g0570500, Os04g0570600, Os04g0570650, Os04g0570700, Os04g0570800, Os04g0570850, Os04g0571200, Os04g0571300, Os04g0571450, Os04g0571600, Os04g0571700, Os04g0571800, Os04g0572100, Os04g0572200, Os04g0572400, Os04g0572500, Os04g0572600, Os04g0572700, Os04g0572800, Os04g0573000, Os04g0573100, Os04g0573200, Os04g0573300, Os04g0573500, Os04g0573700, Os04g0573900, Os04g0574000, Os04g0574100, Os04g0574200, Os04g0574350, Os04g0574500, Os04g0574600, Os04g0574700, Os04g0574800, Os04g0575801, Os04g0576200, Os04g0576250, Os04g0576300, Os04g0576400, Os04g0576500, Os04g0576600, Os04g0576700, Os04g0576800, Os04g0576850, Os04g0576900, Os04g0577000, Os04g0577200, Os04g0577300, Os04g0577337, Os04g0577375, Os04g0577450, Os04g0577500, Os04g0577600, Os04g0577700, Os04g0577800, Os04g0578000, Os04g0578200, Os04g0578250, Os04g0578300, Os04g0578400, Os04g0578500, Os04g0578550, Os04g0578600, Os04g0578700, Os04g0578800, Os04g0578833, Os04g0578900, Os04g0579000, Os04g0579200, Os04g0579600, Os04g0579700, Os04g0580001, Os04g0580300, Os04g0580350, Os04g0580400, Os04g0580450, Os04g0580500, Os04g0580700, Os04g0580800, Os04g0580866 | 87 |
| 5.1 | Os05g0165900, Os05g0166300, Os05g0166450, Os05g0166525, Os05g0166600, Os05g0166833, Os05g0166900, Os05g0167100, Os05g0167300, Os05g0167600, Os05g0167800, Os05g0168002, Os05g0168200, Os05g0168300, Os05g0168400, Os05g0168500, Os05g0168700, Os05g0168800, Os05g0169000, Os05g0169100, Os05g0169200, Os05g0169300, Os05g0169400, Os05g0169450, Os05g0169500, Os05g0169600, Os05g0169700, Os05g0169701, Os05g0169800, Os05g0169801, Os05g0169900, Os05g0170000, Os05g0170200, Os05g0170250, Os05g0170300, Os05g0170425, Os05g0170600, Os05g0170700, Os05g0170800, Os05g0170950, Os05g0171000, Os05g0171050, Os05g0171200, Os05g0171300, Os05g0171450, Os05g0171600, Os05g0171750, Os05g0171900, Os05g0172000, Os05g0172100, Os05g0172300, Os05g0172400, Os05g0172500, Os05g0172800, Os05g0173500, Os05g0173600, Os05g0173700, Os05g0173800, Os05g0173900, Os05g0175000, Os05g0175033 | 61 |
| 5.2 | Os05g0198950, Os05g0199100, Os05g0199301, Os05g0199500, Os05g0199700, Os05g0199800, Os05g0199950, Os05g0200100, Os05g0200160, Os05g0200220, Os05g0200280, Os05g0200340, Os05g0200400, Os05g0200500, Os05g0200601, Os05g0200700, Os05g0201300, Os05g0201333, Os05g0201366, Os05g0201400, Os05g0201700, Os05g0201800, Os05g0201900, Os05g0202000, Os05g0202100, Os05g0202200, Os05g0202300, Os05g0202500, Os05g0202550, Os05g0202600, Os05g0202800, Os05g0202900, Os05g0203000, Os05g0203150, Os05g0203300, Os05g0203400, Os05g0203500, Os05g0203800, Os05g0203900, Os05g0203912, Os05g0204037, Os05g0204163, Os05g0204400, Os05g0204600, Os05g0204750, Os05g0204900, Os05g0204950, Os05g0205000, Os05g0205050, Os05g0205100, Os05g0205400, Os05g0205500, Os05g0205800, Os05g0206000, Os05g0206100, Os05g0206200, Os05g0206300, Os05g0206400, Os05g0206500, Os05g0206600, Os05g0206800, Os05g0207000, Os05g0207200, Os05g0207300, Os05g0207350, Os05g0207400, Os05g0207500, Os05g0207600, Os05g0207700, Os05g0207900, Os05g0208000, Os05g0208050, Os05g0208100, Os05g0208500, Os05g0208550, Os05g0208600, Os05g0209000, Os05g0209100, Os05g0209200, Os05g0209301, Os05g0209400, Os05g0209500, Os05g0209600, Os05g0209683, Os05g0209766, Os05g0209950, Os05g0210100, Os05g0210201, Os05g0210300, Os05g0210400, Os05g0210450, Os05g0210500, Os05g0210525, Os05g0210550, Os05g0210600, Os05g0210700, Os05g0210900 | 9.7 |
| 5.3 | Os05g0228650, Os05g0228900, Os05g0229000, Os05g0229475, Os05g0229900, Os05g0229950, Os05g0230200, Os05g0230501, Os05g0230600, Os05g0230700, Os05g0230900, Os05g0231100, Os05g0231600, Os05g0231700, Os05g0231800, Os05g0231900, Os05g0232200, Os05g0232300, Os05g0232400, Os05g0232500, Os05g0232566, Os05g0232632, Os05g0232700, Os05g0232800, Os05g0233000, Os05g0233100, Os05g0233300, Os05g0234000, Os05g0234366, Os05g0234833 | 30 |
| 5.4 | Os05g0296900, Os05g0297001, Os05g0297150, Os05g0297300, Os05g0297400, Os05g0297600, Os05g0297800, Os05g0297850, Os05g0297900, Os05g0298050, Os05g0298200, Os05g0298300, Os05g0298700, Os05g0298800, Os05g0298900, Os05g0299000, Os05g0299100, Os05g0299101, Os05g0299200, Os05g0299250, Os05g0299300, Os05g0299500, Os05g0299600, Os05g0299651, Os05g0299700, Os05g0300700, Os05g0301500, Os05g0301600, Os05g0301700, Os05g0301825, Os05g0301950, Os05g0302075, Os05g0302200, Os05g0302300, Os05g0302350, Os05g0302400, Os05g0302600, Os05g0302700, Os05g0302750, Os05g0302833, Os05g0302916, Os05g0303000, Os05g0303133 | 43 |
| 5.6 | Os05g0399400, Os05g0399700, Os05g0399800, Os05g0399850, Os05g0399900, Os05g0400100, Os05g0400200, Os05g0400301, Os05g0400400, Os05g0400500, Os05g0400600, Os05g0400700, Os05g0400800, Os05g0401000, Os05g0401050, Os05g0401100, Os05g0401200, Os05g0401300, Os05g0401500, Os05g0402300, Os05g0402400, Os05g0402700, Os05g0402800, Os05g0402851, Os05g0402900, Os05g0403000, Os05g0403100, Os05g0403200, Os05g0403300, Os05g0403400, Os05g0403600 | 31 |
| 5.7 | Os05g0441500, Os05g0441625, Os05g0441750, Os05g0441875, Os05g0442000, Os05g0442100, Os05g0442400, Os05g0442550, Os05g0442700, Os05g0442750, Os05g0442800, Os05g0442900, Os05g0443200, Os05g0443300, Os05g0443400, Os05g0443500, Os05g0443700, Os05g0443800, Os05g0443850, Os05g0443900, Os05g0444200, Os05g0444300, Os05g0444500, Os05g0444750, Os05g0444875, Os05g0445000, Os05g0445100, Os05g0445200, Os05g0445500, Os05g0445650, Os05g0445800, Os05g0445900, Os05g0446300, Os05g0446500, Os05g0446600, Os05g0446700, Os05g0446800, Os05g0446900, Os05g0447000, Os05g0447200, Os05g0447250, Os05g0447300, Os05g0447400, Os05g0447500, Os05g0447540, Os05g0447580, Os05g0447620, Os05g0447660, Os05g0447680, Os05g0447700, Os05g0447800, Os05g0447900, Os05g0448000, Os05g0448300, Os05g0448650, Os05g0448675, Os05g0448700, Os05g0449100, Os05g0449200, Os05g0449250, Os05g0449300, Os05g0449400, Os05g0449500, Os05g0449600, Os05g0449750, Os05g0449900, Os05g0450200, Os05g0450300, Os05g0450600, Os05g0450733, Os05g0450866, Os05g0451100, Os05g0451200, Os05g0451300, Os05g0451601 | 7.5 |
| 5.8 | Os05g0485150, Os05g0485300, Os05g0485400, Os05g0485500, Os05g0485600, Os05g0485800, Os05g0485850, Os05g0485900, Os05g0486100, Os05g0486150, Os05g0486200, Os05g0486300, Os05g0486600, Os05g0486650, Os05g0486700, Os05g0486833, Os05g0486966, Os05g0487100, Os05g0487300, Os05g0487400, Os05g0487500, Os05g0487600, Os05g0487801, Os05g0488000, Os05g0488100, Os05g0488500, Os05g0488600, Os05g0488800, Os05g0488900, Os05g0489000, Os05g0489100, Os05g0489200, Os05g0489301, Os05g0489400, Os05g0489500, Os05g0489600, Os05g0489700, Os05g0489750, Os05g0489800, Os05g0489900, Os05g0490000, Os05g0490100, Os05g0490150, Os05g0490200, Os05g0490300, Os05g0490400, Os05g0490450, Os05g0490500, Os05g0490600, Os05g0490700, Os05g0490800, Os05g0490900, Os05g0491000, Os05g0491100, Os05g0491200, Os05g0491400, Os05g0491500, Os05g0491700, Os05g0491800, Os05g0491900, Os05g0492000, Os05g0492100, Os05g0492200, Os05g0492300, Os05g0492400, Os05g0492500, Os05g0492600, Os05g0493050, Os05g0493100, Os05g0493133, Os05g0493166, Os05g0493200, Os05g0493400, Os05g0493500, Os05g0493600, Os05g0493800, Os05g0494000, Os05g0494050, Os05g0494100, Os05g0494200, Os05g0494300, Os05g0494500, Os05g0494600, Os05g0494700, Os05g0494800, Os05g0494900 | 86 |
| 6.1 | Os06g0163200, Os06g0163300, Os06g0163350, Os06g0163400, Os06g0163450, Os06g0163500, Os06g0163600, Os06g0163701, Os06g0163900, Os06g0164000, Os06g0164100, Os06g0164300, Os06g0164400, Os06g0164500, Os06g0164600, Os06g0164800, Os06g0164900, Os06g0165000, Os06g0165100, Os06g0165201, Os06g0165300, Os06g0165500, Os06g0165600, Os06g0165700, Os06g0165800, Os06g0165900, Os06g0166000, Os06g0166050, Os06g0166100, Os06g0166200, Os06g0166400, Os06g0166500, Os06g0166800, Os06g0166900, Os06g0167000, Os06g0167100, Os06g0167125, Os06g0167150, Os06g0167200, Os06g0167400, Os06g0167500, Os06g0167600, Os06g0167800, Os06g0168000, Os06g0168150, Os06g0168300, Os06g0168400, Os06g0168500, Os06g0168600, Os06g0168700, Os06g0168800, Os06g0168901, Os06g0169001, Os06g0169100, Os06g0169600, Os06g0169700, Os06g0169800, Os06g0169900, Os06g0170000, Os06g0170100, Os06g0170200, Os06g0170366, Os06g0170500, Os06g0170700, Os06g0170750, Os06g0170766, Os06g0170800, Os06g0170833, Os06g0170866, Os06g0171400, Os06g0171450, Os06g0171500 | 72 |
| 6.2 | Os06g0335400, Os06g0335500, Os06g0335600, Os06g0335775, Os06g0335900, Os06g0335950, Os06g0335975, Os06g0336001, Os06g0336101, Os06g0336150, Os06g0336200, Os06g0336350, Os06g0336401, Os06g0336500, Os06g0337000, Os06g0337100, Os06g0337200, Os06g0337300, Os06g0337400, Os06g0337500, Os06g0338100, Os06g0338200, Os06g0338700, Os06g0338801, Os06g0338900, Os06g0339033, Os06g0339066, Os06g0339302, Os06g0339500, Os06g0339800, Os06g0340001, Os06g0340180, Os06g0340200, Os06g0340301, Os06g0340600, Os06g0340833, Os06g0341066, Os06g0341300, Os06g0341400, Os06g0341500, Os06g0341600, Os06g0341800, Os06g0341801, Os06g0342000, Os06g0342100, Os06g0342200, Os06g0342500, Os06g0342750, Os06g0342875, Os06g0343100, Os06g0343200, Os06g0343500, Os06g0343600, Os06g0343700, Os06g0343900, Os06g0344201, Os06g0344500, Os06g0344900, Os06g0345050, Os06g0345200, Os06g0345732, Os06g0346000, Os06g0346100, Os06g0346201, Os06g0346300, Os06g0346400, Os06g0346600 | 67 |
| 6.3 | Os06g0346900, Os06g0347000, Os06g0347100, Os06g0347200, Os06g0347300, Os06g0347700, Os06g0347966, Os06g0348750, Os06g0348800, Os06g0349700, Os06g0349750, Os06g0349800, Os06g0350200, Os06g0350400, Os06g0350600, Os06g0350700 | 16 |
| 7.1 | Os07g0153600, Os07g0153700, Os07g0154100, Os07g0154201, Os07g0154300, Os07g0154400, Os07g0154800, Os07g0154900, Os07g0155001, Os07g0155100, Os07g0155200, Os07g0155600, Os07g0156200, Os07g0156467, Os07g0156732, Os07g0156821, Os07g0156910, Os07g0157000, Os07g0157401, Os07g0157600, Os07g0157700, Os07g0157900, Os07g0158000, Os07g0158100, Os07g0158150, Os07g0158200, Os07g0158300, Os07g0158400, Os07g0158500, Os07g0158800, Os07g0158900, Os07g0159200, Os07g0159300, Os07g0159500, Os07g0159550, Os07g0159600, Os07g0159700, Os07g0159800, Os07g0159900, Os07g0160000, Os07g0160050, Os07g0160100, Os07g0160232, Os07g0160300, Os07g0160400, Os07g0160500, Os07g0160600, Os07g0160800, Os07g0161000, Os07g0161100, Os07g0161500, Os07g0161600, Os07g0161650, Os07g0161700, Os07g0161900, Os07g0162000, Os07g0162100, Os07g0162200, Os07g0162300, Os07g0162400, Os07g0162450, Os07g0162500, Os07g0162600, Os07g0162700, Os07g0162900, Os07g0163000, Os07g0163066, Os07g0163132, Os07g0163200, Os07g0163400, Os07g0163500, Os07g0163800, Os07g0163900, Os07g0164000, Os07g0164100, Os07g0164200, Os07g0164300, Os07g0164500, Os07g0164600, Os07g0164700, Os07g0164800, Os07g0164900, Os07g0165000, Os07g0165100, Os07g0165200, Os07g0165300, Os07g0165650, Os07g0165700, Os07g0165800, Os07g0165900, Os07g0166100, Os07g0166300, Os07g0166500, Os07g0166600, Os07g0166700 | 95 |
| 7.2 | Os07g0179700, Os07g0179950, Os07g0180000, Os07g0180100, Os07g0180300, Os07g0180700, Os07g0180800, Os07g0180900, Os07g0181000, Os07g0181100, Os07g0181200, Os07g0181400, Os07g0181500, Os07g0181700, Os07g0181750, Os07g0181800, Os07g0182000, Os07g0182100, Os07g0182200, Os07g0182400, Os07g0182501, Os07g0182601, Os07g0182900, Os07g0183000, Os07g0183050, Os07g0183100, Os07g0183200, Os07g0183350, Os07g0183400, Os07g0183500, Os07g0183650, Os07g0183700, Os07g0183866, Os07g0184032, Os07g0184200, Os07g0184300, Os07g0184500, Os07g0184633, Os07g0184800, Os07g0184850, Os07g0184900, Os07g0184950, Os07g0185000, Os07g0185100, Os07g0185200, Os07g0185300, Os07g0185366, Os07g0185401, Os07g0185432, Os07g0185500, Os07g0185600, Os07g0185700, Os07g0185800, Os07g0185900, Os07g0186000, Os07g0186100, Os07g0186150, Os07g0186200, Os07g0186400, Os07g0186500, Os07g0187000, Os07g0187001 | 62 |
| 7.3 | Os07g0191650, Os07g0191700, Os07g0191801, Os07g0191900, Os07g0192000, Os07g0192050, Os07g0192100, Os07g0192300, Os07g0192400, Os07g0192550, Os07g0192700, Os07g0192800, Os07g0192900, Os07g0193000, Os07g0193100, Os07g0193200, Os07g0193400, Os07g0193500, Os07g0193600, Os07g0193701, Os07g0193800, Os07g0193900, Os07g0194000, Os07g0194100, Os07g0194300, Os07g0194351, Os07g0194400, Os07g0194500, Os07g0194550, Os07g0194675, Os07g0194800, Os07g0194950, Os07g0195100, Os07g0195200, Os07g0195300, Os07g0195350, Os07g0195400, Os07g0195500, Os07g0195600, Os07g0195800, Os07g0196000, Os07g0196100, Os07g0196200, Os07g0196300, Os07g0196500, Os07g0196600, Os07g0196700, Os07g0196800, Os07g0196900, Os07g0197000, Os07g0197100, Os07g0197300, Os07g0197350, Os07g0197400, Os07g0197500, Os07g0198000, Os07g0198300, Os07g0198475, Os07g0198650, Os07g0199000, Os07g0199350, Os07g0199700, Os07g0199850, Os07g0200000, Os07g0200500, Os07g0200700, Os07g0200801, Os07g0200900, Os07g0201000, Os07g0201100, Os07g0201300, Os07g0201402, Os07g0201500, Os07g0201650, Os07g0201800, Os07g0201901, Os07g0202000, Os07g0202100, Os07g0202300, Os07g0202400, Os07g0202600, Os07g0202900, Os07g0203000, Os07g0203100, Os07g0203200, Os07g0203250, Os07g0203300, Os07g0203400, Os07g0203500, Os07g0203600, Os07g0203700, Os07g0203900, Os07g0203925, Os07g0203950, Os07g0204000, Os07g0204100, Os07g0204250, Os07g0204400, Os07g0204500, Os07g0204650, Os07g0204800, Os07g0204900, Os07g0205000, Os07g0205200, Os07g0205350, Os07g0205500, Os07g0205600, Os07g0205700, Os07g0205800, Os07g0205900, Os07g0206300, Os07g0206400, Os07g0206500, Os07g0206600, Os07g0206650, Os07g0206700, Os07g0206750, Os07g0206800, Os07g0206850, Os07g0206900, Os07g0207100, Os07g0207200, Os07g0207301, Os07g0207400, Os07g0207600, Os07g0207700, Os07g0207800, Os07g0207900, Os07g0208000, Os07g0208050, Os07g0208100, Os07g0208200, Os07g0208500, Os07g0208533, Os07g0208566, Os07g0208600, Os07g0208700 | 137 |
| 7.4 | Os07g0228900, Os07g0229100, Os07g0229200, Os07g0229500, Os07g0229700, Os07g0229800, Os07g0229900, Os07g0230400, Os07g0230500, Os07g0230600, Os07g0230700, Os07g0231050, Os07g0231133, Os07g0231216, Os07g0231300, Os07g0231400, Os07g0231500, Os07g0231700, Os07g0231800, Os07g0231900, Os07g0232000, Os07g0232100, Os07g0232200, Os07g0232300, Os07g0232450, Os07g0232600, Os07g0232800, Os07g0232850, Os07g0232900, Os07g0232950, Os07g0233000, Os07g0233200, Os07g0233300, Os07g0233500, Os07g0234100, Os07g0234600, Os07g0234700, Os07g0234900, Os07g0235200, Os07g0235400, Os07g0235600, Os07g0235625, Os07g0235650, Os07g0235700, Os07g0235800, Os07g0236200, Os07g0236300, Os07g0236700, Os07g0236800, Os07g0237100, Os07g0237133, Os07g0237166, Os07g0237200, Os07g0237700, Os07g0237900, Os07g0238000, Os07g0238500, Os07g0238600, Os07g0238700, Os07g0238800, Os07g0239000, Os07g0239200, Os07g0239400, Os07g0239500, Os07g0239600, Os07g0239667, Os07g0239734, Os07g0239802, Os07g0240200, Os07g0240300, Os07g0240400, Os07g0240600, Os07g0241050, Os07g0241500, Os07g0241600, Os07g0241700, Os07g0241751, Os07g0241800, Os07g0241866, Os07g0242000, Os07g0242050, Os07g0242166, Os07g0242282, Os07g0242400, Os07g0242600, Os07g0242700 | 86 |
| 7.5 | Os07g0419650, Os07g0419800, Os07g0419850, Os07g0419900, Os07g0420000, Os07g0420400, Os07g0420700, Os07g0420900, Os07g0421000, Os07g0421100, Os07g0421150, Os07g0421200, Os07g0421300, Os07g0421450, Os07g0421600, Os07g0421800, Os07g0421866, Os07g0421932, Os07g0422000, Os07g0422100, Os07g0422250, Os07g0422280, Os07g0422400, Os07g0422700, Os07g0422800, Os07g0423000, Os07g0423350, Os07g0423550, Os07g0423700, Os07g0424000, Os07g0424300, Os07g0424400, Os07g0424600, Os07g0425000, Os07g0425450, Os07g0426833, Os07g0428216, Os07g0429600, Os07g0429700, Os07g0430501, Os07g0431160, Os07g0432100, Os07g0432201, Os07g0432333, Os07g0432566, Os07g0432800, Os07g0434100, Os07g0434125, Os07g0434183, Os07g0434241, Os07g0434300, Os07g0434401, Os07g0434500, Os07g0434700, Os07g0435000, Os07g0435100, Os07g0435200 | 57 |
| 7.6 | Os07g0438700, Os07g0438800, Os07g0438900, Os07g0439000, Os07g0439100, Os07g0439750, Os07g0440000, Os07g0440100, Os07g0440200, Os07g0440300, Os07g0440501, Os07g0440700, Os07g0440800, Os07g0440900, Os07g0441300, Os07g0442000, Os07g0442201, Os07g0442401, Os07g0442800, Os07g0442900, Os07g0443101, Os07g0443302, Os07g0443500, Os07g0443700, Os07g0444000, Os07g0444650, Os07g0445300, Os07g0445450, Os07g0445600, Os07g0445800, Os07g0446000, Os07g0446050, Os07g0446100, Os07g0446400, Os07g0446600, Os07g0446800, Os07g0446900, Os07g0447000, Os07g0447200, Os07g0447501 | 40 |
| 7.7 | Os07g0454100, Os07g0454200, Os07g0454400, Os07g0454550, Os07g0454700, Os07g0455100, Os07g0456000, Os07g0456033, Os07g0456066, Os07g0456400, Os07g0456500, Os07g0456600, Os07g0456700, Os07g0456800, Os07g0456900, Os07g0457100, Os07g0457200, Os07g0457300, Os07g0457400, Os07g0457500, Os07g0457550, Os07g0458025, Os07g0458500, Os07g0458700, Os07g0458800, Os07g0459000, Os07g0459050, Os07g0459100, Os07g0459200, Os07g0459400, Os07g0459500, Os07g0459600, Os07g0460900, Os07g0461466, Os07g0461500, Os07g0461550, Os07g0461600, Os07g0461700, Os07g0461800, Os07g0461900, Os07g0461950, Os07g0462000, Os07g0462066, Os07g0462132, Os07g0462200, Os07g0462450, Os07g0462500, Os07g0462600, Os07g0462700, Os07g0462800, Os07g0463100, Os07g0463400, Os07g0463500, Os07g0463600, Os07g0463800, Os07g0463900, Os07g0464001, Os07g0464050, Os07g0464099, Os07g0464148, Os07g0464200, Os07g0464400, Os07g0464600, Os07g0464700, Os07g0465100, Os07g0465200, Os07g0465400 | 67 |
| 7.8 | Os07g0489500, Os07g0489650, Os07g0489800, Os07g0489950, Os07g0490100, Os07g0490200, Os07g0490300, Os07g0490400, Os07g0490450, Os07g0490500, Os07g0490600, Os07g0490700, Os07g0490800, Os07g0491500, Os07g0491600, Os07g0491700, Os07g0491800, Os07g0491900, Os07g0491950, Os07g0492000, Os07g0492100, Os07g0492150, Os07g0492200, Os07g0492250, Os07g0492300, Os07g0492500, Os07g0492700, Os07g0492800, Os07g0492900, Os07g0492966, Os07g0493033, Os07g0493100, Os07g0493200, Os07g0493332, Os07g0493400, Os07g0493601, Os07g0493800, Os07g0493825, Os07g0493850, Os07g0494200, Os07g0494300, Os07g0494500, Os07g0494700, Os07g0494800, Os07g0494900, Os07g0495000, Os07g0495100, Os07g0495150, Os07g0495175, Os07g0495200, Os07g0495250, Os07g0495300, Os07g0495900, Os07g0495950, Os07g0496000, Os07g0496200, Os07g0496250, Os07g0496300, Os07g0496401, Os07g0496500, Os07g0496600, Os07g0496700, Os07g0496900, Os07g0497100, Os07g0497400, Os07g0497500, Os07g0497800, Os07g0498000, Os07g0498100, Os07g0498150, Os07g0498300 | 71 |
| 7.9 | Os07g0552900, Os07g0553000, Os07g0553100, Os07g0553300, Os07g0553400, Os07g0553450, Os07g0553600, Os07g0553633, Os07g0553700, Os07g0553800 | 10 |
| 8.1 | Os08g0135700, Os08g0135800, Os08g0135900, Os08g0136001, Os08g0136100, Os08g0136300, Os08g0136366, Os08g0136432, Os08g0136500, Os08g0136600, Os08g0136700, Os08g0136725, Os08g0136750, Os08g0136800, Os08g0137000, Os08g0137050, Os08g0137100, Os08g0137200, Os08g0137225, Os08g0137250, Os08g0137300, Os08g0137350, Os08g0137400, Os08g0137600, Os08g0137800, Os08g0137900, Os08g0138100, Os08g0138200, Os08g0138301, Os08g0138400, Os08g0138500, Os08g0138600, Os08g0138700, Os08g0138900, Os08g0139000, Os08g0139100, Os08g0139200, Os08g0139300, Os08g0139400, Os08g0139500, Os08g0139600, Os08g0139650, Os08g0139700, Os08g0140000, Os08g0140300, Os08g0140500, Os08g0140600, Os08g0140700, Os08g0140850, Os08g0140925, Os08g0141000, Os08g0141100, Os08g0141200, Os08g0141300, Os08g0141400, Os08g0141500, Os08g0141600, Os08g0141700, Os08g0142000, Os08g0142100, Os08g0142200, Os08g0142300, Os08g0142400, Os08g0142500, Os08g0142900, Os08g0143150, Os08g0143300, Os08g0143400, Os08g0143500, Os08g0143600, Os08g0143700, Os08g0143900, Os08g0144000, Os08g0144050, Os08g0144100, Os08g0144400, Os08g0145501, Os08g0145600, Os08g0146001, Os08g0146400, Os08g0146701, Os08g0147001, Os08g0147300, Os08g0148100, Os08g0148200, Os08g0148267, Os08g0148300, Os08g0148400, Os08g0148500, Os08g0148533, Os08g0148566, Os08g0148600, Os08g0149000, Os08g0149100 | 94 |
| 8.2 | Os08g0210300, Os08g0211025, Os08g0211750, Os08g0212475, Os08g0213200, Os08g0213400, Os08g0213816, Os08g0214200, Os08g0214233, Os08g0214300, Os08g0214600, Os08g0214900, Os08g0215200, Os08g0215300, Os08g0215400, Os08g0215500, Os08g0215900, Os08g0216000, Os08g0216050, Os08g0216200, Os08g0216300, Os08g0216450, Os08g0216600, Os08g0216900, Os08g0217050, Os08g0217200, Os08g0217350, Os08g0217800, Os08g0218000, Os08g0218700, Os08g0218900, Os08g0218950, Os08g0219100, Os08g0219200 | 34 |
| 8.3 | Os08g0227400, Os08g0227500, Os08g0227600, Os08g0227750, Os08g0227900, Os08g0228100, Os08g0228200, Os08g0228550, Os08g0228900, Os08g0229100, Os08g0229150, Os08g0229200, Os08g0229500, Os08g0229601, Os08g0230000, Os08g0230100, Os08g0230200, Os08g0230300, Os08g0230350, Os08g0230400, Os08g0230500, Os08g0230600, Os08g0230800, Os08g0230900, Os08g0231100, Os08g0231400, Os08g0231801, Os08g0232000, Os08g0232100, Os08g0232201, Os08g0232700, Os08g0233000, Os08g0233300, Os08g0233400, Os08g0233600, Os08g0233900, Os08g0234000, Os08g0234050, Os08g0234100, Os08g0234200, Os08g0234400, Os08g0234700, Os08g0234900, Os08g0235100, Os08g0235183, Os08g0235266, Os08g0235333, Os08g0235400, Os08g0235500, Os08g0235550, Os08g0235600, Os08g0235650, Os08g0235651, Os08g0235700, Os08g0235800, Os08g0236000, Os08g0236400, Os08g0236700, Os08g0236800, Os08g0236866, Os08g0236900, Os08g0237000, Os08g0237100, Os08g0237200, Os08g0237500 | 65 |
| 8.4 | Os08g0267000, Os08g0267300, Os08g0267425, Os08g0267450, Os08g0267600, Os08g0267700, Os08g0267800, Os08g0268000, Os08g0268550, Os08g0268900, Os08g0269000, Os08g0269300, Os08g0269500, Os08g0269600, Os08g0269700, Os08g0269800, Os08g0270000, Os08g0270200, Os08g0270400, Os08g0270500, Os08g0270700, Os08g0270800, Os08g0270900, Os08g0271150, Os08g0271400, Os08g0271600, Os08g0271800, Os08g0272000, Os08g0272200, Os08g0272400, Os08g0272601, Os08g0272800, Os08g0272900, Os08g0272950, Os08g0273000, Os08g0273600, Os08g0273783, Os08g0273966, Os08g0274150, Os08g0274700, Os08g0274775, Os08g0274850, Os08g0275000, Os08g0275200, Os08g0275600, Os08g0276000, Os08g0276100, Os08g0276200, Os08g0276400, Os08g0276801, Os08g0277200, Os08g0277250, Os08g0277300, Os08g0277900, Os08g0278100, Os08g0278400 | 56 |
| 8.5 | Os08g0282400, Os08g0282500, Os08g0282600, Os08g0282700, Os08g0283000, Os08g0283300, Os08g0283600, Os08g0283900, Os08g0284200, Os08g0284500, Os08g0284800, Os08g0285100, Os08g0285200, Os08g0285301, Os08g0285350, Os08g0285600, Os08g0286100, Os08g0286300, Os08g0286500, Os08g0287200, Os08g0287700, Os08g0287800, Os08g0288000, Os08g0288050, Os08g0288100, Os08g0288200, Os08g0288300, Os08g0288400, Os08g0288500, Os08g0288600, Os08g0288800, Os08g0288901, Os08g0289000, Os08g0289133, Os08g0289266, Os08g0289400, Os08g0290000, Os08g0290100, Os08g0290200, Os08g0290300, Os08g0290400, Os08g0290500, Os08g0290700, Os08g0290900, Os08g0291100, Os08g0291700, Os08g0292000, Os08g0292400, Os08g0292600, Os08g0292750, Os08g0292900, Os08g0293000, Os08g0293100, Os08g0293200, Os08g0293250, Os08g0293300, Os08g0293400, Os08g0294300, Os08g0294600, Os08g0294800, Os08g0294900, Os08g0295000, Os08g0295100, Os08g0295200, Os08g0295300, Os08g0295900, Os08g0296600, Os08g0296700, Os08g0296900, Os08g0297500, Os08g0297650, Os08g0297800, Os08g0298500, Os08g0298700, Os08g0298750, Os08g0298800, Os08g0298901, Os08g0299000 | 78 |
| 8.6 | Os08g0357700, Os08g0358050, Os08g0358425, Os08g0358800, Os08g0358900, Os08g0359000, Os08g0359050, Os08g0359100, Os08g0359200, Os08g0359300, Os08g0359400, Os08g0359500, Os08g0359550, Os08g0359600, Os08g0359900, Os08g0360000, Os08g0360100, Os08g0360150, Os08g0360200, Os08g0360201, Os08g0360300, Os08g0360350, Os08g0360400, Os08g0360500, Os08g0360700, Os08g0360800, Os08g0361000, Os08g0361300, Os08g0361400, Os08g0361501, Os08g0361600, Os08g0362816, Os08g0362832, Os08g0362850, Os08g0363000, Os08g0363200, Os08g0363800, Os08g0363900, Os08g0364100, Os08g0364250, Os08g0364300, Os08g0364400, Os08g0364500, Os08g0364900, Os08g0365050, Os08g0365200, Os08g0365500, Os08g0365800, Os08g0365900, Os08g0366000, Os08g0366050, Os08g0366100, Os08g0366200, Os08g0366300, Os08g0366425, Os08g0366550, Os08g0366675, Os08g0366800, Os08g0367300, Os08g0367400, Os08g0367625, Os08g0367850, Os08g0368000, Os08g0368166, Os08g0368333, Os08g0368555, Os08g0368777, Os08g0369000, Os08g0369066, Os08g0369132, Os08g0369200, Os08g0369300, Os08g0369400, Os08g0369600, Os08g0369700, Os08g0369800, Os08g0371200, Os08g0371608, Os08g0371800, Os08g0372166, Os08g0372401, Os08g0372700, Os08g0372900, Os08g0373000, Os08g0373400, Os08g0373900 | 86 |
| 8.7 | Os08g0395400, Os08g0395500, Os08g0395700, Os08g0395800, Os08g0396200, Os08g0396301, Os08g0396401, Os08g0396500, Os08g0396700, Os08g0397250, Os08g0397800, Os08g0397900, Os08g0398000, Os08g0398300, Os08g0398350, Os08g0398400, Os08g0398450, Os08g0398500, Os08g0398700, Os08g0398800, Os08g0399050, Os08g0399300, Os08g0399400, Os08g0399500, Os08g0399600, Os08g0399900, Os08g0400000, Os08g0400200, Os08g0400300, Os08g0400800, Os08g0401100, Os08g0401200, Os08g0401301, Os08g0401500, Os08g0401800, Os08g0401901, Os08g0402001, Os08g0402100, Os08g0402234, Os08g0402366, Os08g0402500, Os08g0402600, Os08g0402650, Os08g0402700, Os08g0402800, Os08g0403300, Os08g0403733, Os08g0404000, Os08g0404200, Os08g0404300, Os08g0404350, Os08g0404400, Os08g0404500, Os08g0404700, Os08g0404900, Os08g0405100, Os08g0405150, Os08g0405275, Os08g0405400, Os08g0405700, Os08g0406150, Os08g0406400, Os08g0406450, Os08g0406500, Os08g0406600, Os08g0406700, Os08g0406900, Os08g0407000, Os08g0407101, Os08g0407200, Os08g0407400, Os08g0407600, Os08g0407700, Os08g0408100, Os08g0408200, Os08g0408300, Os08g0408500, Os08g0408700, Os08g0409100, Os08g0409233, Os08g0409366, Os08g0409500, Os08g0409700, Os08g0409900, Os08g0410100, Os08g0410233, Os08g0410266, Os08g0410350, Os08g0410500, Os08g0410800, Os08g0410851, Os08g0410900, Os08g0410950, Os08g0411000, Os08g0411100, Os08g0411200, Os08g0411250, Os08g0411300, Os08g0411500, Os08g0411800, Os08g0411850, Os08g0411900, Os08g0412001, Os08g0412100, Os08g0412200 | 105 |
| 9.5 | Os09g0462550, Os09g0462700, Os09g0462850, Os09g0462875, Os09g0462901, Os09g0463100, Os09g0463166, Os09g0463232, Os09g0463300, Os09g0463450, Os09g0463600, Os09g0463700, Os09g0463800, Os09g0463900, Os09g0464000, Os09g0464033, Os09g0464066, Os09g0464100, Os09g0464300, Os09g0464350, Os09g0464400, Os09g0464800, Os09g0465200, Os09g0465400, Os09g0465500, Os09g0465600, Os09g0465800, Os09g0466100, Os09g0466201, Os09g0466300, Os09g0466400, Os09g0466800, Os09g0466900, Os09g0467100, Os09g0467200, Os09g0467300, Os09g0467400, Os09g0467700, Os09g0468000, Os09g0468150, Os09g0468300, Os09g0468600, Os09g0468650, Os09g0468700, Os09g0468800, Os09g0468850, Os09g0468900, Os09g0469300, Os09g0469400, Os09g0469600, Os09g0469900, Os09g0469950, Os09g0470000 | 53 |
| 9.6 | Os09g0489500, Os09g0489800, Os09g0490200, Os09g0490400, Os09g0490750, Os09g0491100, Os09g0491238, Os09g0491376, Os09g0491516, Os09g0491532, Os09g0491564, Os09g0491596, Os09g0491612, Os09g0491628, Os09g0491644, Os09g0491652, Os09g0491660, Os09g0491676, Os09g0491692, Os09g0491708, Os09g0491724, Os09g0491740, Os09g0491756, Os09g0491772, Os09g0491780, Os09g0491788, Os09g0491789, Os09g0491804, Os09g0491820, Os09g0491822, Os09g0491852, Os09g0491868, Os09g0491884, Os09g0492700, Os09g0492733, Os09g0492800, Os09g0492900, Os09g0493000, Os09g0493200, Os09g0493400, Os09g0493500, Os09g0493600, Os09g0493700, Os09g0493800, Os09g0494200, Os09g0494300, Os09g0494500, Os09g0494600, Os09g0494800, Os09g0495000, Os09g0495100, Os09g0495200, Os09g0495300, Os09g0495500, Os09g0496250, Os09g0497000, Os09g0497100, Os09g0497400, Os09g0497500, Os09g0497600, Os09g0497700, Os09g0497900, Os09g0498000, Os09g0498100, Os09g0498200, Os09g0498300, Os09g0498400, Os09g0498500, Os09g0498600, Os09g0498700, Os09g0498800, Os09g0499000, Os09g0499201, Os09g0499300, Os09g0499400, Os09g0499450, Os09g0499500, Os09g0499600, Os09g0500100, Os09g0500151, Os09g0500200, Os09g0500300, Os09g0500600 | 83 |
| 9.7 | Os09g0500900, Os09g0500966, Os09g0501032, Os09g0501100, Os09g0501150, Os09g0501200, Os09g0501600, Os09g0501650, Os09g0501700, Os09g0501825, Os09g0501850, Os09g0502000, Os09g0502033, Os09g0502066, Os09g0502100, Os09g0502150, Os09g0502200, Os09g0502350, Os09g0502500, Os09g0502566, Os09g0502600, Os09g0502700, Os09g0502800, Os09g0502900, Os09g0502950, Os09g0503000, Os09g0503051, Os09g0503100, Os09g0503250, Os09g0503400, Os09g0503700, Os09g0503850, Os09g0504000, Os09g0504400, Os09g0504700, Os09g0504800, Os09g0504900, Os09g0505000, Os09g0505050, Os09g0505100, Os09g0505200, Os09g0505300, Os09g0505400, Os09g0505600, Os09g0505700, Os09g0505800, Os09g0505850, Os09g0505900, Os09g0505950, Os09g0506000, Os09g0506150, Os09g0506301, Os09g0506450, Os09g0506525, Os09g0506600, Os09g0506700, Os09g0506750, Os09g0506800, Os09g0506900, Os09g0507100, Os09g0507200, Os09g0507300, Os09g0507350, Os09g0507400, Os09g0507500, Os09g0507550, Os09g0507600, Os09g0507701, Os09g0507800, Os09g0508000, Os09g0508200, Os09g0508250, Os09g0508300, Os09g0508401, Os09g0508500, Os09g0508900, Os09g0508951, Os09g0509000, Os09g0509050, Os09g0509100, Os09g0509200, Os09g0509300, Os09g0509350, Os09g0509400, Os09g0509450, Os09g0509500, Os09g0509700, Os09g0509800, Os09g0509900, Os09g0510000, Os09g0510200, Os09g0510500, Os09g0510700, Os09g0510800, Os09g0510900, Os09g0511000, Os09g0511200, Os09g0511300, Os09g0511500, Os09g0511600, Os09g0511650, Os09g0511700, Os09g0511900, Os09g0512000, Os09g0512066, Os09g0512132, Os09g0512200, Os09g0512300, Os09g0512450, Os09g0512600, Os09g0512700, Os09g0512750, Os09g0512800, Os09g0512900, Os09g0512950, Os09g0513000, Os09g0513100, Os09g0513200, Os09g0513400, Os09g0513500, Os09g0513600, Os09g0513700, Os09g0513800, Os09g0513850, Os09g0513900, Os09g0514001, Os09g0514100, Os09g0514200, Os09g0514300, Os09g0514350, Os09g0514400, Os09g0514500, Os09g0514550, Os09g0514600 | 134 |
| 10.1 | Os10g0202200, Os10g0202350, Os10g0202501, Os10g0202900, Os10g0203000, Os10g0203100, Os10g0203600, Os10g0204000, Os10g0204100, Os10g0204200, Os10g0204201, Os10g0204300, Os10g0204400, Os10g0205200, Os10g0205300, Os10g0205400, Os10g0205500, Os10g0205700, Os10g0205802, Os10g0206000, Os10g0206400, Os10g0206500, Os10g0206650, Os10g0206800, Os10g0207101, Os10g0207300, Os10g0207400, Os10g0207500, Os10g0207600, Os10g0207700, Os10g0208200, Os10g0208250, Os10g0208300, Os10g0208500, Os10g0208600, Os10g0208750, Os10g0208900, Os10g0209100, Os10g0209300, Os10g0209433, Os10g0209566, Os10g0209700, Os10g0210500, Os10g0211800, Os10g0211900, Os10g0212100, Os10g0213100, Os10g0213700, Os10g0213800, Os10g0213966, Os10g0214132, Os10g0214300, Os10g0214400, Os10g0214501, Os10g0233310, Os10g0254720 | 56 |
| 10.2 | Os10g0321700, Os10g0321866, Os10g0322032, Os10g0322166, Os10g0322300, Os10g0322600, Os10g0323000, Os10g0323400, Os10g0323500, Os10g0323600, Os10g0323900, Os10g0324100, Os10g0324301, Os10g0324600, Os10g0324900, Os10g0325150, Os10g0325400, Os10g0325750, Os10g0326100, Os10g0326200, Os10g0326400, Os10g0326800, Os10g0326900, Os10g0327000, Os10g0327050, Os10g0327100, Os10g0327200, Os10g0327201, Os10g0327300, Os10g0327325, Os10g0327351, Os10g0327400, Os10g0327500, Os10g0327600, Os10g0327700, Os10g0327800, Os10g0327901, Os10g0328001, Os10g0328100, Os10g0328600, Os10g0328700, Os10g0328900, Os10g0329300, Os10g0329400, Os10g0329401, Os10g0329900, Os10g0330000, Os10g0330300, Os10g0330400, Os10g0330600, Os10g0330675, Os10g0330750, Os10g0330825, Os10g0330900, Os10g0331400, Os10g0331500, Os10g0331600, Os10g0331733, Os10g0331866, Os10g0332000, Os10g0332300, Os10g0332600, Os10g0332733, Os10g0332866, Os10g0333000, Os10g0333100, Os10g0333401, Os10g0333700, Os10g0334500, Os10g0334750, Os10g0335000, Os10g0335050, Os10g0335100, Os10g0335200 | 74 |
| 11.1 | Os11g0116200, Os11g0116300, Os11g0116400, Os11g0116500, Os11g0116550, Os11g0116600, Os11g0116900, Os11g0117300, Os11g0117400, Os11g0117450, Os11g0117500, Os11g0117600, Os11g0117700, Os11g0117801, Os11g0117900, Os11g0118000, Os11g0118200, Os11g0118300, Os11g0118350, Os11g0118400, Os11g0118500, Os11g0118600, Os11g0118800, Os11g0118875, Os11g0118950, Os11g0119025, Os11g0119100, Os11g0119200, Os11g0119311, Os11g0119422, Os11g0119533, Os11g0119700, Os11g0119800, Os11g0119900, Os11g0120100, Os11g0120200, Os11g0120300, Os11g0120600, Os11g0120725, Os11g0120850, Os11g0121000, Os11g0121300, Os11g0121400, Os11g0121450, Os11g0121500, Os11g0121600, Os11g0121800, Os11g0122150, Os11g0122800, Os11g0123001, Os11g0123023, Os11g0123033, Os11g0123066, Os11g0123100, Os11g0123250, Os11g0123400, Os11g0123500, Os11g0123600, Os11g0123950, Os11g0124300, Os11g0124500, Os11g0124700, Os11g0124800, Os11g0124900, Os11g0125100, Os11g0125500, Os11g0125601, Os11g0125700, Os11g0125900, Os11g0126100, Os11g0126250, Os11g0126400, Os11g0126500, Os11g0126800, Os11g0126900, Os11g0127000, Os11g0127600, Os11g0127700, Os11g0127800, Os11g0127900, Os11g0127951, Os11g0128000, Os11g0128300, Os11g0128400, Os11g0128500, Os11g0128600, Os11g0128700, Os11g0128800, Os11g0128932, Os11g0129000, Os11g0129101, Os11g0129200, Os11g0129301, Os11g0129400, Os11g0129500, Os11g0129600, Os11g0129700, Os11g0129800, Os11g0130000, Os11g0130100, Os11g0130200, Os11g0130300, Os11g0130400, Os11g0130500, Os11g0130600, Os11g0130700, Os11g0130800, Os11g0130900, Os11g0131100, Os11g0131200, Os11g0131300, Os11g0131400, Os11g0131500, Os11g0131600 | 114 |
| 11.3 | Os11g0275400, Os11g0275500, Os11g0275600, Os11g0275800, Os11g0275850, Os11g0275900, Os11g0276000, Os11g0276100, Os11g0276300, Os11g0276550, Os11g0276700, Os11g0277100, Os11g0278200, Os11g0278300, Os11g0278900, Os11g0279750, Os11g0280600, Os11g0282100, Os11g0282300, Os11g0282600, Os11g0282700, Os11g0282800, Os11g0282900, Os11g0283150, Os11g0283500, Os11g0283766, Os11g0284032, Os11g0284300, Os11g0284400, Os11g0284500, Os11g0284600, Os11g0284900, Os11g0284950, Os11g0285000, Os11g0285466, Os11g0285932, Os11g0286216, Os11g0286400, Os11g0286500, Os11g0286800, Os11g0287000, Os11g0287100, Os11g0288350, Os11g0289700, Os11g0290150, Os11g0290600, Os11g0291000, Os11g0291201, Os11g0291400, Os11g0291450, Os11g0291475, Os11g0291500 | 52 |
| 11.4 | Os11g0421800, Os11g0422000, Os11g0422100, Os11g0423200, Os11g0423501, Os11g0423800, Os11g0424200, Os11g0424400 | 8 |
| 11.5 | Os11g0524051, Os11g0524300, Os11g0524400, Os11g0524601, Os11g0524900, Os11g0525200, Os11g0525500, Os11g0525600, Os11g0525700, Os11g0525800, Os11g0525900, Os11g0526000, Os11g0526200, Os11g0526400, Os11g0526601, Os11g0526800, Os11g0526900, Os11g0527000, Os11g0527100, Os11g0527150, Os11g0527200, Os11g0527250, Os11g0527300, Os11g0527400, Os11g0527500, Os11g0527600, Os11g0527700, Os11g0527701, Os11g0527900, Os11g0528001, Os11g0528200, Os11g0528300, Os11g0528400, Os11g0528500, Os11g0528700, Os11g0528812, Os11g0528924, Os11g0529036, Os11g0529100, Os11g0529125, Os11g0529150, Os11g0529500, Os11g0529550, Os11g0529700, Os11g0529800, Os11g0529833, Os11g0529900, Os11g0530000, Os11g0530050, Os11g0530600, Os11g0530650, Os11g0530700, Os11g0531000, Os11g0531150, Os11g0531300, Os11g0531600, Os11g0531700, Os11g0531800, Os11g0532000, Os11g0532200, Os11g0532600, Os11g0532751, Os11g0532900, Os11g0533000, Os11g0533100, Os11g0533400, Os11g0533500, Os11g0533550, Os11g0533600, Os11g0533700, Os11g0533800, Os11g0533900, Os11g0534100, Os11g0534300, Os11g0534500, Os11g0535100, Os11g0535525, Os11g0535600, Os11g0536000, Os11g0536400, Os11g0536800, Os11g0537300, Os11g0537350, Os11g0537375, Os11g0537400 | 85 |
| 12.1 | Os12g0211200, Os12g0211301, Os12g0211400, Os12g0211500, Os12g0211600, Os12g0211900, Os12g0212100, Os12g0212200, Os12g0212300, Os12g0212366, Os12g0212400, Os12g0212600, Os12g0214600, Os12g0214700, Os12g0215100, Os12g0215700, Os12g0215800, Os12g0215900, Os12g0215925, Os12g0215950, Os12g0216000, Os12g0216500, Os12g0216766, Os12g0217032, Os12g0217300, Os12g0217400, Os12g0217500, Os12g0217600, Os12g0217800, Os12g0218100, Os12g0218300, Os12g0218500, Os12g0218600, Os12g0218701, Os12g0218800, Os12g0218900, Os12g0219100, Os12g0219300, Os12g0219700, Os12g0219900, Os12g0220001, Os12g0220100, Os12g0220800, Os12g0220833, Os12g0220866, Os12g0220900, Os12g0221000, Os12g0221100, Os12g0221400, Os12g0221501, Os12g0221600, Os12g0221700, Os12g0221800, Os12g0222300 | 54 |
| 12.2 | Os12g0287500, Os12g0287550, Os12g0287600, Os12g0287800, Os12g0288000, Os12g0288266, Os12g0288400, Os12g0288600, Os12g0288801, Os12g0288900, Os12g0289301, Os12g0289600, Os12g0289666, Os12g0289800, Os12g0289833, Os12g0289866, Os12g0289900, Os12g0289901, Os12g0290100, Os12g0290150, Os12g0290200, Os12g0290400, Os12g0290501, Os12g0290600, Os12g0290800, Os12g0291000, Os12g0291033, Os12g0291066, Os12g0291100, Os12g0291200, Os12g0291301, Os12g0291400, Os12g0291733, Os12g0292200, Os12g0292301, Os12g0292400, Os12g0292900, Os12g0293100, Os12g0293601, Os12g0294100, Os12g0295600, Os12g0295700, Os12g0295900, Os12g0296200, Os12g0297400, Os12g0297500, Os12g0297901, Os12g0298300, Os12g0298600, Os12g0298950 | 50 |
| 12.3 | Os12g0563600, Os12g0563700, Os12g0564000, Os12g0564100, Os12g0564400, Os12g0564600, Os12g0564701, Os12g0564750, Os12g0564800, Os12g0564866, Os12g0564932, Os12g0565000, Os12g0565100, Os12g0565133, Os12g0565166, Os12g0565200, Os12g0565300, Os12g0565450, Os12g0565800, Os12g0566000, Os12g0566050, Os12g0566100, Os12g0566200, Os12g0566250, Os12g0566300, Os12g0566400, Os12g0566500, Os12g0566600, Os12g0566700, Os12g0566800, Os12g0566900, Os12g0567000, Os12g0567100, Os12g0567150, Os12g0567200, Os12g0567300, Os12g0567500, Os12g0567700, Os12g0567800, Os12g0567900, Os12g0568100, Os12g0568166, Os12g0568200, Os12g0568275, Os12g0568350, Os12g0568500, Os12g0568600, Os12g0568700, Os12g0568750 | 49 |
