# Supplementary material for: Genomic dissection of iron toxicity tolerance in rice identifies key loci, candidate genes, and associated haplotypes
Source: Sci Rep. 2026 Mar 9;16:12767. doi: 10.1038/s41598-026-38841-9 (PMC13096636; doi:10.1038/s41598-026-38841-9)
Supplement: Supplementary file 9 — Supplementary Material 9 [file 41598_2026_38841_MOESM9_ESM.docx]

**Table: Transcription factors identified among 284 CGs**

| **Locus ID** | **Gene Symbol** | **Oryzabase Gene Name Synonym(s)** |
| --- | --- | --- |
| Os11g0117600 | WRKY50 | Rice WRKY gene50 |
| Os11g0117400 | WRKY104 | collar region-preferential gene 32 |
| Os01g0571800 | - | - |
| Os09g0489500 | BZIP74 | b-ZIP transcription factor 74, TGA factor 10, TGACGTCA cis-element-binding protein 10 |
| Os11g0126900 | NAC122 | NAC domain-containing protein 10, NAC domain-containing protein 122 |
| Os11g0127600 | NAC45 | NAC domain-containing protein 045, NAC domain-containing protein 45, DLN repressor 243, DLN motif protein 243, No Apical Meristem |
| Os11g0116900 | WRKY46 | WRKY GENE 91 |
| Os01g0571300 | HSFA7 | Heat stress transcription factor A7, Heat stress transcription factor A-6a, Heat stress transcription factor 1, endosperm-specific gene 9 |
| Os02g0266800 | RITA1 | b-ZIP transcription factor 20, rice seed b-Zipper 3, rice transcription activator-1 |
| Os05g0442700 | NAC12 | NAC domain-containing protein 012, NAC domain-containing protein 12 |
| Os02g0698800 | WRKY66 | Rice WRKY gene66 |
| Os07g0182000 | RISBZ1 | bZIP transcription factor 58, rice seed b-Zipper 1, endosperm-specific gene 92, seed maturation factor 1, rice seed basic leucine zipper 1 |
| Os03g0741400 | WRKY121 |  |
| Os03g0802900 | OsbHLH089 | basic helix-loop-helix protein 089 |
| Os08g0408500 | ERF48 | ethylene response factor 48, APETALA2/ethylene-responsive element binding protein 170, Drought Responsive AP2/EREBP gene 1 |
| Os09g0509700 | BBX28 | B-box-containing protein 28, CCT domain-containing gene 32, CCT (CO, CO-LIKE and TOC1) domain protein 32, CCT domain protein 32 |
| Os05g0488600 | DLN147 | DLN repressor 147, DLN motif protein 147 |
| Os05g0444200 | DLN142, ZOS5-08 | DLN repressor 142, DLN motif protein 142, zinc-finger protein TFIIIA class of Oryza sativa 5-08, ZPT of Oryza sativa 5-08 |
| Os02g0624300 | MYB30 | Myb transcription factor 4 paralog, OsMyb4 paralog, R2R3-MYB Transcription Factor 26, R2R3-MYB transcription factor 2-29 |
| Os07g0443500 | MYB family transcription factor | MYB family transcription factor |
| Os12g0564100 | 2R_MYB97 | R2R3-MYB Transcription Factor 97, R2R3-MYB transcription factor 2-115 |
