# Supplementary material for: Genomic dissection of iron toxicity tolerance in rice identifies key loci, candidate genes, and associated haplotypes
Source: Sci Rep. 2026 Mar 9;16:12767. doi: 10.1038/s41598-026-38841-9 (PMC13096636; doi:10.1038/s41598-026-38841-9)
Supplement: Supplementary file 11 — Supplementary Material 11 [file 41598_2026_38841_MOESM11_ESM.docx]

| Table S3. List of 284 CGs underlying 63 M-QTLs | | | | |
| --- | --- | --- | --- | --- |
| RAP locus ID | **Description** | **Gene Symbol (RAP-DB)** | **Gene Symbol (CGSNL)** | **Gene Symbol (Oryzabase)** |
| Os01g0266800 | Cystinosin/ERS1p repeat containing protein. (Os01t0266800-01) |  |  |  |
| Os01g0155000 | Alpha/beta hydrolase fold-3 domain containing protein. (Os01t0155000-02) |  | HSR203J | Hsr203j, OsHsr203j |
| Os01g0503400 | Similar to (Rice Genome Annotation Project) metal transporter Nramp6. (Os01t0503400-01);Similar to cDNA clone:J013164K10, full insert sequence. (Os01t0503400-02);Iron and manganese transporter, Negative regulation of disease resistance to the rice blast fungus (Os01t0503400-03);Iron and manganese transporter, Target of osa-miR7695, Negative regulation of disease resistance to the rice blast fungus (Os01t0503400-04);Similar to cDNA clone:J013164K10, full insert sequence. (Os01t0503400-05) | NRAMP6, l-Nramp6, Nramp6, OsNramp6.8, s-Nramp6 | NRAMP6 | OsNRAMP6, Nramp6, l-NRAMP6, s-NRAMP6, OsNramp6.8, OsNramp1 |
| Os01g0609300 | PDR-type ABC transporter 9 (Os01t0609300-01);Hypothetical conserved gene. (Os01t0609300-02) | OsPDR9 | PDR9 | OsPDR9, ospdr9, OsPDR3, PDR3, OsABCG36/OsPDR9, OsABCG36 |
| Os01g0151500 | Similar to GGT4 (GAMMA-GLUTAMYL TRANSPEPTIDASE 4); gamma-glutamyltransferase/ glutathione gamma-glutamylcysteinyltransferase. (Os01t0151500-00) |  | | |
| Os01g0606000 | MtN3 and saliva related transmembrane protein family protein. (Os01t0606000-00) |  | SWEET6A | OsSWEET6a, SWEET6a |
| Os01g0606900 | Heat shock protein DnaJ, N-terminal domain containing protein. (Os01t0606900-01) |  | BPH33 | OsDjC10, DjC10, Bph33(t), Bph33 |
| Os01g0152000 | LRR-receptor-like kinase (LRR-RLK) family protein (Os01t0152000-01) |  |  |  |
| Os01g0823100 | Alpha-expansin OsEXPA2. (Os01t0823100-01) |  | EXPA2 | OsEXP2, OsEXP2(Os-EXP2), Os-EXP2, EXPA2, OsEXPA2, OsaEXPa1.23, RiExB, RiExC, Os-EXPA2 |
| Os01g0823300 | Similar to Ribosomal protein S26. (Os01t0823300-00) |  | RPS26 | OsRPS26 |
| Os01g0266500 | Phenazine biosynthesis PhzC/PhzF protein family protein. (Os01t0266500-01) |  |  |  |
| Os01g0269000 | Similar to Hydroxymethylglutaryl-CoA lyase. (Os01t0269000-01) |  |  |  |
| Os01g0369700 | Similar to Glutathione S-transferase GST 8 (EC 2.5.1.18). (Os01t0369700-01);Similar to Glutathione S-transferase GST 8 (EC 2.5.1.18). (Os01t0369700-03) |  | GSTF5 | OsGSTF5, OsGST F5 |
| Os01g0371400 | Similar to Glutathione s-transferase gstf2. (Os01t0371400-00) |  | GSTF9 | OsGSTF9 |
| Os01g0371500 | Similar to Glutathione-S-transferase 19E50. (Os01t0371500-01) |  | GSTF10 | OsGSTF10 |
| Os01g0393400 | Similar to MDR-like ABC transporter. (Os01t0393400-00) |  |  |  |
| Os01g0444100 | Conserved hypothetical protein. (Os01t0444100-00) |  |  |  |
| Os01g0498300 | Xylan 2-O-xylosyl/2-O-arabinosyl transferase 1 (Os01t0498300-01) | OsXXAT1 | OsXXAT1 |  |
| Os01g0501700 | Sec-independent translocase protein (Os01t0501700-01) | OsTATC | TATC | OsTATC, OsTatC, TatC |
| Os01g0571166 | Similar to gibberellin responsive1. (Os01t0571166-00) |  |  |  |
| Os01g0571300 | Heat shock transcription factor, Salt and drought tolerance (Os01t0571300-01) | OsHsfA7 | HSFA7 | OsHsfA7, OsHsf-01, HSFA6B, HSF01, OsHSF1, HSF1, OsEnS-9, OsHsfA7a, HsfA7a |
| Os01g0571800 | Lateral organ boundaries, LOB domain containing protein. (Os01t0571800-00) |  |  |  |
| Os01g0607900 | LRR-receptor-like kinase (LRR-RLK) family protein (Os01t0607900-01) |  | OsRPK1, OsJNipponRPK1, OsI219RPK1, OsI9311RPK1 | OsRPK1, OsJNipponRPK1, OsI219RPK1, OsI9311RPK1 |
| Os01g0608300 | Similar to aspartic proteinase nepenthesin-2. (Os01t0608300-00) |  |  |  |
| Os01g0608400 | Nucleotide-binding LRR receptor (NLR) family protein (Os01t0608400-01) |  |  |  |
| Os01g0609000 | Similar to Pleiotropic drug resistance protein 3. (Os01t0609000-00) |  | PDR10 | OsABCG34, OsPDR10, OsPDR1 |
| Os01g0609900 | Similar to Pleiotropic drug resistance protein 4. (Os01t0609900-01);Similar to PDR-like ABC transporter (PDR4 ABC transporter). (Os01t0609900-02) |  | PDR8 | OsPDR8, PDR4, P0410E03.16, OsABCG37, OsPDR4 |
| Os01g0822200 | Protein kinase, core domain containing protein. (Os01t0822200-01) |  | RLCK47 | OsRLCK47 |
| Os01g0823500 | OVATE family protein, Regulation of growth and development, Resistance to drought and cold stresses (Os01t0823500-01) | OsOFP6 | OFP4 | OsOFP4, OsOFP06, OsOFP6, OFP6 |
| Os02g0550600 | Alkaline/neutral invertase, Root cell development, Pollen development, Regulation of floral transition (Os02t0550600-01) | OsCYT-INV1, OsCyt-inv1 | NIN8 | OsNIN8 |
| Os02g0270200 | Similar to Subtilase. (Os02t0270200-01) |  | SUB14 | OsSub14 |
| Os02g0618100 | Glutaredoxin (Grx) family protein, Arsenic (As) stress response, Drought tolerance, Facilitation of iron utilization (Os02t0618100-01) | OsGRX9, OsGrx_C2.1 | GRX9 | OsGRX9, OsGrx_C2.1, Grx_C2.1 |
| Os02g0689900 | Low-affinity nitrate transporter, Ortholog of Arabidopsis NRT1.5/AtNPF7.3, Mediation of nitrate allocation (Os02t0689900-01) | OsNPF7.9 | NRT1.5A | OsNRT1.5A |
| Os02g0551100 | Serine/threonine protein kinase, Dehydration-inducible SNF1-related protein kinase 2, Hyperosmotic stress response, Abscisic acid (ABA) signaling (Os02t0551100-01) | SAPK6, OsSAPK6, OSRK1 | SAPK6 | OsSAPK6, OSRK1, OsSnRK2.6, SnRK2.6 |
| Os02g0690000 | Conserved hypothetical protein. (Os02t0690000-01) |  |  |  |
| Os02g0697500 | Similar to Selenium-binding protein-like. (Os02t0697500-01);Pentatricopeptide repeat-containing protein (Os02t0697500-02) |  | | |
| Os02g0699600 | Similar to 60S ribosomal protein L12. (Os02t0699600-01) |  |  |  |
| Os02g0265900 | Reticulon family protein. (Os02t0265900-01);Hypothetical conserved gene. (Os02t0265900-02);Reticulon family protein. (Os02t0265900-03);Similar to Reticulon. (Os02t0265900-04) |  | | |
| Os02g0266000 | Similar to N-(5'-phosphoribosyl)anthranilate isomerase. (Os02t0266000-01) |  |  |  |
| Os02g0266300 | Similar to Transient receptor potential cation channel subfamily A member 1 (Ankyrin-like with transmembrane domains protein 1) (Transformation sensitive-protein p120). (Os02t0266300-01);Similar to Transient receptor potential cation channel subfamily A member 1 (Ankyrin-like with transmembrane domains protein 1) (Transformation sensitive-protein p120). (Os02t0266300-02) |  | | |
| Os02g0266800 | bZIP transcriptional activator, Tolerance to salt and drought stresses (Os02t0266800-01) | RISBZ3, RITA1, RITA-1, OsbZIP20 | RITA1 | OsbZIP20, bZIP20, RITA-1, RISBZ3 |
| Os02g0267200 | Alpha-expansin OsEXPA13. (Os02t0267200-00) |  | EXPA13 | OsEXPA13, OsEXP13, osaEXPa1.13 |
| Os02g0269600 | Similar to Subtilase. (Os02t0269600-00) |  | SUB13 | OsSub13 |
| Os02g0270350 | Hypothetical protein. (Os02t0270350-00) |  |  |  |
| Os02g0312600 | Rac-like GTP-binding protein 7. (Os02t0312600-01) |  | RAC7 | OSRAC7, OsRac7, ROP5, OsRop5 |
| Os02g0313900 | Similar to Plasma membrane H+-ATPase. (Os02t0313900-01) |  |  |  |
| Os02g0549200 | Similar to Ser Thr specific protein kinase-like protein. (Os02t0549200-01) |  | RLCK73 | OsRLCK73 |
| Os02g0552400 | Similar to electron carrier/ ubiquinol-cytochrome-c reductase. (Os02t0552400-00) |  |  |  |
| Os02g0620400 | RmlC-like jelly roll fold domain containing protein. (Os02t0620400-01) |  |  |  |
| Os02g0621300 | Homologous protein of CER1, Very-long-chain (VLC) alkane biosynthesis, Regulation of anther development and plastids differentiation (Os02t0621300-01) | OsCER1 | LTR1 | OsCER1, CER1, OsGL1-4, GL1-4, Os CER1, OsLTR1 |
| Os02g0624300 | R2R3-type MYB transcription factor, Negative regulation of cold tolerance, Maintenance of Pi homeostasis (Os02t0624300-01) | OsMYB30, OsMYB5P | MYB30 | OsMYB30, OsMYB5P, MYB5P, OsMYB13a, MYB13a, Os2R_MYB26, 2R_MYB26 |
| Os02g0626100 | Similar to Phenylalanine ammonia-lyase. (Os02t0626100-01);Similar to Phenylalanine ammonia-lyase. (Os02t0626100-02);Similar to Phenylalanine ammonia-lyase. (Os02t0626100-03);Non-protein coding transcript. (Os02t0626100-04) |  | PAL1 | OsPAL, Pal1*, Pal1, PAL, Pal_1, PalI, PAL2, OsPAL01, PAL01, OSPAL, CP-1, GP-1, OsPAL1, PAL3, OsPAL3 |
| Os02g0690600 | Zinc finger, RING/FYVE/PHD-type domain containing protein. (Os02t0690600-01) |  | PUB36 | OsPUB36, PUB36 |
| Os02g0694800 | Similar to ATP binding / protein kinase. (Os02t0694800-01) |  |  |  |
| Os02g0695600 | Conserved hypothetical protein. (Os02t0695600-01) |  |  |  |
| Os02g0697400 | 4-coumarate:coenzyme A ligase, Flavonoid biosynthesis (Os02t0697400-01) | 4CL2, Os4CL2 | 4CL2 | Os4CL2 |
| Os02g0697600 | AAA-ATPase family protein, Resistance to blast fungus (Os02t0697600-01);Similar to ATPase family AAA domain-containing protein 3. (Os02t0697600-02) | OsAAA-ATPase4 | |  |
| Os02g0698800 | WRKY transcription factor 66. (Os02t0698800-01) |  | WRKY66 | OsWRKY66 |
| Os02g0700600 | Similar to GAMYB-binding protein. (Os02t0700600-01);Similar to Cyclin-dependent kinase F-4. (Os02t0700600-02);Similar to cDNA clone:J013158B11, full insert sequence. (Os02t0700600-03);Similar to Cyclin-dependent kinase F-4. (Os02t0700600-04);Non-protein coding transcript. (Os02t0700600-05) |  | CDKF;4 | CDKF;4, Orysa;CDKF;4 |
| Os02g0702000 | Pentatricopeptide repeats protein, Splicing of chloroplast mRNA, Regulation of chloroplast development (Os02t0702000-01) | OSOTP51, SSA1 | SSA1 | OSOTP 51, OSOTP51, OTP51, OsOTP51, OsSSA1 |
| Os03g0570100 | Similar to Cytochrome P450 79A1 (EC 1.14.13.41) (Tyrosine N-monooxygenase) (Cytochrome P450Tyr). (Os03t0570100-01) |  | | |
| Os03g0154000 | Aromatic-ring hydroxylase family protein. (Os03t0154000-01) |  |  |  |
| Os03g0212800 | Beta-glucosidase, ABA recycling, Drought tolerance, Control of photosynthesis (Os03t0212800-01) | Os3BGlu6 | BGLU6 | Os3bglu6, Os3BGlu6, 3BGlu6 |
| Os03g0267000 | Small heat shock protein, Heavy metal resistance, Regulation of Cu tolerance, Regulation of bacterial leaf streak (BLS) and bacterial blight (BB) resistance (Os03t0267000-01) | OsMSR3, OsHsp18.0-CI | HSP18.0 | OsHSP18.0-CI, HSP18.0-CI, OsHsp18.0, OsHSP18.0, Oshsp18.0, OsSHSP1, SHSP1, OsMSR3, MSR3, OsHSP20, HSP20, OsHSP18.6, HSP18.6 |
| Os03g0277300 | Heat shock protein 70. (Os03t0277300-01) |  | HSP70 | OsHSP70, OsEnS-45, EnS-45 |
| Os03g0692700 | Similar to Pherophorin-S precursor. (Os03t0692700-01);Hypothetical conserved gene. (Os03t0692700-03);Similar to predicted protein. (Os03t0692700-04);Similar to predicted protein. (Os03t0692700-05) |  | | |
| Os03g0802500 | AAA-ATPase family protein, Salicylic acid (SA)-mediated defense response against blast fungus M. oryzae (Os03t0802500-01) | OsAAA-ATPase1 | |  |
| Os03g0809000 | Plant disease resistance response protein family protein. (Os03t0809000-01) |  | DIR11 | OsDIR11, OsjDIR11 |
| Os03g0815200 | Similar to Methylenetetrahydrofolate reductase (EC 1.5.1.20). (Os03t0815200-01);Similar to Methylenetetrahydrofolate reductase. (Os03t0815200-02);Similar to Methylenetetrahydrofolate reductase. (Os03t0815200-03);Similar to Methylenetetrahydrofolate reductase (Fragment). (Os03t0815200-04) |  | | |
| Os03g0219900 | Similar to 50S ribosomal protein L15, chloroplast precursor (CL15) (Fragment). (Os03t0219900-01) |  | |  |
| Os03g0699300 | Similar to Adenylosuccinate synthetase, chloroplast precursor (EC 6.3.4.4) (IMP-- aspartate ligase) (AdSS) (AMPSase). (Os03t0699300-01) |  | ASS1 | OsASS1 |
| Os03g0701200 | Similar to Sugar-starvation induced protein (Fragment). (Os03t0701200-01) |  |  |  |
| Os03g0806600 | Conserved hypothetical protein. (Os03t0806600-01) |  |  |  |
| Os03g0807000 | Conserved hypothetical protein. (Os03t0807000-01) |  |  |  |
| Os03g0812000 | Probable DNA gyrase subunit A (Os03t0812000-01) |  |  |  |
| Os03g0815400 | Ribosomal protein L17-like protein. (Os03t0815400-01) |  |  |  |
| Os03g0150800 | Pi transporter, Pi homeostasis, Selenite uptake (Os03t0150800-01) | PT2, OsPT2, PTH1-2, OsPht1;2 | PT2 | OsPT2, PTH1-2, OsPht1;2, PHT1;2, OsPHT1;2 |
| Os03g0152000 | Heavy metal transport/detoxification protein domain containing protein. (Os03t0152000-01) |  | HIPP36 | OsHMP18, HMP18, OsHIPP36, OsaHIP36, HIP36 |
| Os03g0152400 | Similar to 4-coumarate--CoA ligase-like 1. (Os03t0152400-01) |  | OsACS1 | OsACS1 |
| Os03g0152700 | Pseudouridine (Ψ) synthase, Chloroplast development at low temperature, Cold tolerance (Os03t0152700-01) | TCD3, OsPUS1 | TCD3 | OsTCD3, OsPUS1, PUS1 |
| Os03g0153900 | Aromatic-ring hydroxylase family protein. (Os03t0153900-01) |  |  |  |
| Os03g0156700 | Zebra-necrosis like protein, Nickel/cobalt transporter, high-affinity domain containing protein (Os03t0156700-01) | OsZNL | OsZNL | OsZNL |
| Os03g0157300 | Ca2+-permeable mechanosensitive channel, Regulation of plasma membrane Ca2+ influx, ROS generation induced by hypo-osmotic stress (Os03t0157300-01);Protein of unknown function Cys-rich family protein. (Os03t0157300-02) | OsMCA1 | DMT1 | OsMCA1, MCA1, OsDMT1, OsDMT1/OsMCA1 |
| Os03g0214400 | Similar to Digalactosyldiacylglycerol synthase 2. (Os03t0214400-01) |  | DGD2A | OsDGD2alpha DGD2alpha, OsDGD2A, OsDGD1, DGD1 |
| Os03g0215000 | Similar to integral membrane family protein. (Os03t0215000-01);Similar to integral membrane family protein. (Os03t0215000-02);Similar to integral membrane family protein. (Os03t0215000-03) |  | | |
| Os03g0218400 | Monosaccharide transporter 4, Monosaccharides supply for seed development during the course of grain filling (Os03t0218400-01) | OsMST4 | MST4 | OsMST4, OsSTP4, STP4 |
| Os03g0218500 | Similar to 70kD heat shock protein. (Os03t0218500-01) |  |  |  |
| Os03g0219100 | Phosphatidylinositol transfer protein, Class III SEC14 protein (Os03t0219100-01) | OsSec14-26 | SEC14-26 | OsSec14-26, Sec14-26 |
| Os03g0219700 | DEAD-like helicase, N-terminal domain containing protein. (Os03t0219700-01) |  | OsRH47B | OsRH47B |
| Os03g0220700 | Similar to (RAP Annotation release2) Peptidase. (Os03t0220700-01);Conserved hypothetical protein. (Os03t0220700-02) |  | | |
| Os03g0221200 | Similar to Homocysteine S-methyltransferase 1 (EC 2.1.1.10) (S- methylmethionine:homocysteine methyltransferase 1) (SMM:Hcy S- methyltransferase 1) (ZmHMT-1). (Os03t0221200-01) |  | HMT3 | OsaHMT3, OsHMT3 |
| Os03g0221700 | S-Domain receptor like kinase-9, Response to submergence and B. glumae in resistant genotype CL 161 at flowering stage (Os03t0221700-02) | SDRLK-9 | SDRLK9 | SDRLK-9, OsSDRLK-9, OsSDRLK9 |
| Os03g0266900 | 17.4 kDa class I small heat shock protein, Heat tolerance (Os03t0266900-02) | OsHsp17.4 | HSP17.3 | OsHsp17.3, OsHSP17.3, Oshsp17.3 |
| Os03g0267800 | Ubiquitin-interacting motif-containing ubiquitin receptor, Homologue of Arabidopsis DA1, Positive regulation of grain size and weight (Os03t0267800-01) | HDR3 | HDR3 | OsHDR3 |
| Os03g0268600 | Similar to Protein phosphatase type 2C. (Os03t0268600-01) |  | PP2C30 | OsPP2C30, OsPP48, PP48 |
| Os03g0269100 | Short chain alcohol dehydrogenase-like. (Os03t0269100-01);Similar to tropinone reductase. (Os03t0269100-02) |  | OsSAG13 | OsSAG13 |
| Os03g0270500 | Protein of unknown function DUF668 family protein. (Os03t0270500-01) |  | DUF668-4 | OsDUF668-4 |
| Os03g0277700 | Protein of unknown function DUF26 domain containing protein. (Os03t0277700-01) |  |  |  |
| Os03g0279200 | Similar to Histone H2A. (Os03t0279200-01) |  |  |  |
| Os03g0279400 | Similar to predicted protein. (Os03t0279400-01);Similar to Arginine biosynthesis bifunctional protein argJ 1 [Includes: Glutamate N-acetyltransferase (EC 2.3.1.35) (Ornithine acetyltransferase) (Ornithine transacetylase) (OATase); Amino-acid acetyltransferase (EC 2.3.1.1) (N-acetylglutamate synthase) (AGS)] [Contains: Arginine biosynthesis bifunctional protein argJ1 alpha chain; Arginine biosynthesis bifunctional protein argJ1 beta chain]. (Os03t0279400-02) |  | | |
| Os03g0571900 | Phenolics efflux transporter, Essential for the utilization of apoplasmic precipitated iron in the stele (Os03t0571900-01) | PEZ1 | PEZ1 | OsPEZ1, OsMATE14, MATE14 |
| Os03g0687000 | Similar to predicted protein. (Os03t0687000-01);Nitrate transporter, Peptide transporter, osa-miR168a target gene, Acquisition and long-distance transport of nitrate, Regulation of seed vigor (Os03t0687000-02) | OsNPF2.4, NPF2.4, OsPTR2, PTR2 | NPF2.4 | OsNPF2.4, OsPTR2, PTR2 |
| Os03g0693800 | Oxalate oxidase 2, Positive regulation of panicle blast resistance (Os03t0693800-01) | OsOXO2 | OXO2 | Osoxo4, OsOxO2, OsGLP3-4, GLP3-4 |
| Os03g0694000 | Oxalate oxidase 4, Positive regulation of panicle blast resistance (Os03t0694000-01) | OsOXO4 | OXO4 | OsOxO4, OsGLP3-6, GLP3-6 |
| Os03g0697200 | Similar to carboxy-lyase. (Os03t0697200-00) |  | LOGL4 | LOGL4 |
| Os03g0700700 | Lipoxygenase, Tolerance to wounding and BPH attack (Os03t0700700-01);Similar to Lipoxygenase. (Os03t0700700-02);Similar to Lipoxygenase. (Os03t0700700-03);Similar to Lipoxygenase. (Os03t0700700-04) | OsLOX1 | LOX1 | OsLOX1, OsLOX5 |
| Os03g0700750 | Hypothetical gene. (Os03t0700750-00) |  |  |  |
| Os03g0741400 | Similar to SUSIBA2. (Os03t0741400-01) |  | WRKY121 | OsWRKY121 |
| Os03g0741600 | Prenylated rab acceptor PRA1 family protein. (Os03t0741600-00) |  |  |  |
| Os03g0743500 | Calcium-binding EF-hand domain containing protein. (Os03t0743500-01);Calmodulin-like protein 4, Salt resistance (Os03t0743500-02) | OsCML4 | CML4 | OsCML4 |
| Os03g0745100 | Similar to cytokinin-O-glucosyltransferase 1. (Os03t0745100-00) |  |  |  |
| Os03g0802900 | Similar to MYC1. (Os03t0802900-01) |  | OsbHLH089 | OsbHLH089, bHLH089, bHLH89 |
| Os03g0803700 | Hypothetical conserved gene. (Os03t0803700-01) |  |  |  |
| Os03g0805400 | Phosphatidic acid phosphatase type 2/haloperoxidase domain containing protein. (Os03t0805400-01);Phosphatidic acid phosphatase type 2/haloperoxidase domain containing protein. (Os03t0805400-02);Similar to phosphoric ester hydrolase. (Os03t0805400-03) |  | | |
| Os03g0805766 | Hypothetical protein. (Os03t0805766-00) |  |  |  |
| Os03g0806700 | Protein of unknown function DUF868, plant family protein. (Os03t0806700-01);Protein of unknown function DUF868, plant family protein. (Os03t0806700-02) |  | | |
| Os03g0807700 | Protein of unknown function DUF642 domain containing protein. (Os03t0807700-01);Similar to predicted protein. (Os03t0807700-02) |  | | |
| Os03g0808100 | Cellulose synthase A2 (Os03t0808100-01);Similar to Cellulose synthase BoCesA5. (Os03t0808100-02);Similar to Cellulose synthase BoCesA5. (Os03t0808100-03) | OsCESA2 | CESA2 | OsCesA2, OsCESA2, OS_CESA02 |
| Os03g0808600 | Calcium-dependent protein kinase 8 (Os03t0808600-01) | OsCPK8 | CDPK8 | OsCDPK8, OsCPK8 |
| Os03g0811100 | Magnesium-chelatase subunit ChlD, Chlorophyll synthesis, Chloroplast development (Os03t0811100-01);Similar to Chloroplast Mg-chelatase subunit XANTHA-G. (Os03t0811100-02) | OsChlD, ChlD, YGL98, Ygl7 | YGL98 | OsChlD, ChlD, CHLD, OsCHLD, Ygl7, YGL3, OsvWA12, vWA12 |
| Os04g0573000 | SPX-MFS protein, Phosphate transporter, Pi homeostasis (Os04t0573000-01) | OsSPX-MFS1 | SPX-MFS1 | OsSPX-MFS1 |
| Os04g0298700 | Clp, N-terminal domain containing protein. (Os04t0298700-01) |  |  |  |
| Os04g0301700 | Glycosyl transferase, family 14 protein. (Os04t0301700-01) |  |  |  |
| Os04g0304200 | Similar to Nonphototropic hypocotyl protein 1 (EC 2.7.1.37) (Phototropin). (Os04t0304200-01);Rice nonphototropic hypocotyl 1 (NPH1) homologue, Phototropin, Ion homeostasis, Salt stress response (Os04t0304200-02) | PHOT2, OsPHOT2, OsNPH1b | NPH1B | PHOT2, OsPHOT2, OsNPH1b, OsAGC9, AGC9 |
| Os04g0306400 | Ribose 5-phosphate isomerase family protein. (Os04t0306400-01) |  | OsRPI, RPI | OsRPI, RPI |
| Os04g0423800 | Peroxidase (EC 1.11.1.7). (Os04t0423800-01) |  | prx54 | prx54 |
| Os04g0569900 | IQ calmodulin-binding region domain containing protein. (Os04t0569900-01);Similar to OSIGBa0111L12.1 protein. (Os04t0569900-02) |  | | |
| Os04g0572500 | Similar to OSIGBa0147H17.2 protein. (Os04t0572500-00) |  |  |  |
| Os04g0573900 | Putative cytochrome P450, CYP704A3, Target gene of miRNA (osa-miRf10422-akr), Control of seed length (Os04t0573900-01);Similar to cDNA clone:001-036-C03, full insert sequence. (Os04t0573900-02);Similar to cytochrome P450. (Os04t0573900-03) | CYP704A3 | CYP704A3 | OsCYP704A3 |
| Os04g0578000 | ACC synthase, Ethylene biosynthesis (Os04t0578000-01) | ACS2, OsACS2, Os-ACS2 | ACS2 | OsACS2, Os-ACS2 |
| Os04g0578600 | Similar to H0404F02.15 protein. (Os04t0578600-01);Ferric reductase oxidase, Iron homeostasis, "Drought, heat, salinity stress response" (Os04t0578600-02) | OsFRO2, OsFRO1 | FRO2 | OsFRO2, OsFRO1 |
| Os04g0579700 | Similar to Predicted protein. (Os04t0579700-01) |  | H-BTB3 | OsH-BTB3 |
| Os05g0166600 | S-Domain receptor like kinase-36 (Os05t0166600-00) | SDRLK-36 | SDRLK36 | SDRLK-36, OsSDRLK-36, OsSDRLK36 |
| Os05g0202800 | Similar to Metallothionein-like protein 3B. (Os05t0202800-00) |  | MTI3B | MTd, OsMT-I-3b, OsMT3b, MT-I-3b, MT3b |
| Os05g0444200 | Similar to T6J4.5 protein (WIP6 protein). (Os05t0444200-01) |  | DLN142 | OsDLN142 |
| Os05g0485300 | TRAM, LAG1 and CLN8 homology domain containing protein. (Os05t0485300-01) |  |  |  |
| Os05g0298200 | Ankyrin repeat containing protein. (Os05t0298200-01);Hypothetical conserved gene. (Os05t0298200-02) |  | |  |
| Os05g0490800 | Similar to Proteasome subunit alpha type. (Os05t0490800-01) |  |  |  |
| Os05g0490900 | Conserved hypothetical protein. (Os05t0490900-01) |  |  |  |
| Os05g0166300 | S-Domain receptor like kinase-35, Response to drought and submergence, Response to short-term cold stress in tolerant genotype Volano, Response to long-term chilling stress in tolerant Li-Jiang-Xin-Tuan-He-Gu (LTH) genotype, Response to Xanthomonas oryzae pv. Oryzae (Os05t0166300-01) | SDRLK-35 | SDRLK35 | SDRLK-35, OsSDRLK-35, OsSDRLK35 |
| Os05g0169700 | Similar to Sugar transporter family protein, expressed. (Os05t0169700-00) |  |  |  |
| Os05g0171200 | Embryo-specific 3 family protein. (Os05t0171200-01) |  |  |  |
| Os05g0171900 | Glyoxalase/bleomycin resistance protein/dioxygenase domain containing protein. (Os05t0171900-01);Similar to Lactoylglutathione lyase. (Os05t0171900-02) |  | GLYI6 | OsGLYI6, OsGLYI6.1, OsGLYI6.2, OsGLYI6.3, OsGLYI6.4, OsGLYI6.5 |
| Os05g0199100 | Similar to NDR1/HIN1-Like protein 2. (Os05t0199100-01) |  |  |  |
| Os05g0200400 | Cytochrome P450 CYP90D5, Pesticide detoxification and degradation, Degradation of herbicides isoproturon and acetochlor (Os05t0200400-01) | CYP90D5 | CYP90D5 | OsCYP90D5 |
| Os05g0209500 | Conserved hypothetical protein. (Os05t0209500-01) |  |  |  |
| Os05g0209600 | GDSL esterase/lipase protein (Os05t0209600-01) | OsGELP63 | GELP63 | OsGELP63 |
| Os05g0210500 | Timeless protein domain containing protein. (Os05t0210500-00) |  |  |  |
| Os05g0210600 | Molecular chaperone, heat shock protein, Hsp40, DnaJ domain containing protein. (Os05t0210600-01) |  | |  |
| Os05g0229475 | Similar to OSIGBa0112G01.4 protein. (Os05t0229475-00) |  |  |  |
| Os05g0232200 | Conserved hypothetical protein. (Os05t0232200-01) |  |  |  |
| Os05g0299500 | Protein of unknown function DUF914, eukaryotic family protein. (Os05t0299500-01) |  |  |  |
| Os05g0401200 | Similar to predicted protein. (Os05t0401200-01) |  |  |  |
| Os05g0402700 | Similar to Fructose-bisphosphate aldolase, cytoplasmic isozyme (EC 4.1.2.13). (Os05t0402700-01) |  | ALDC2 | GSC 233, AldC-2, OsAldC-2, OsFBA, FBA |
| Os05g0402900 | Similar to EDGP. (Os05t0402900-01) |  | OsBphi262 | OsBphi262 |
| Os05g0442700 | No apical meristem (NAM) protein domain containing protein. (Os05t0442700-01) |  | NAC12 | ONAC012, ONAC12 |
| Os05g0444300 | Protein of unknown function DUF914, eukaryotic family protein. (Os05t0444300-01) |  |  |  |
| Os05g0445100 | Cytochrome P450 94C4, Cytochrome P450 of the CYP94 subfamily, Salt stress response (Os05t0445100-01);Cytochrome P450 domain containing protein. (Os05t0445100-02) | CYP94C4, OsCYP94C4 | CYP94C4 | OsCYP94C4 |
| Os05g0488600 | Similar to Phosphate starvation regulator protein (Regulatory protein of P- starvation acclimation response Psr1). (Os05t0488600-01) |  | DLN147 | OsDLN147, OsDLN147a, OsDLN147b, DLN147a, DLN147b |
| Os05g0489900 | Ca/calmodulin-dependent protein kinase, Calcium/calmodulin-dependent protein kinase, Bacterial and fungal symbioses, Common symbiosis signaling (SYM) pathway, Regulation of saline-alkaline tolerance in root (Os05t0489900-01) | DMI3, OsDMI3, OsCCaMK1, OsCCaMK, OsCCAMK, CCAMK | DMI3 | OsDMI3, OsCCaMK1, OsCCaMK, OsCCAMK, CCAMK |
| Os05g0494200 | Similar to Cystatin (Fragment). (Os05t0494200-01);Cysteine proteinase inhibitor (cystatin), Oryzacystatin-II (Os05t0494200-02) | OC-II, OC-III | OC2 | Oc2*, Oc2, OC-II, OC-III, OCII, OsCYS2 |
| Os06g0338200 | Copper amine oxidase family protein. (Os06t0338200-01) |  | DAO1 | OsDAO1, OsCuDAO1, CuDAO1 |
| Os06g0338700 | Similar to Primary amine oxidase. (Os06t0338700-00) |  |  |  |
| Os06g0163300 | Harpin-induced 1 domain containing protein. (Os06t0163300-01) |  |  |  |
| Os06g0165500 | S-Domain receptor like kinase-37, Response to drought in tolerant genotypes (Os06t0165500-00) | SDRLK-37 | RLCK198 | OsRLCK198, SDRLK-37, OsSDRLK-37, SDRLK37, OsSDRLK37 |
| Os06g0166000 | Cyclin-like F-box domain containing protein. (Os06t0166000-01);Similar to F-box domain containing protein. (Os06t0166000-02) |  | OsFbox298 | OsFbox298, Os_F0486, OsFBL28, FBL28 |
| Os06g0166500 | Aux/IAA protein, Mediation of abiotic stress tolerance, Drought and salt tolerance (Os06t0166500-01) | OsIAA20 | IAA20 | OsIAA20 |
| Os06g0168000 | Glutathione S-transferase, C-terminal-like domain containing protein. (Os06t0168000-01) |  |  |  |
| Os06g0168500 | Qa-SNARE (soluble N-ethylmaleimide sensitive factor attachment protein receptor), t-SNARE, Regulation of arbuscular mycorrhizal (AM) symbiosis (Os06t0168500-01) | OsSYP131b | SYP131 | OsSYP131, OsSYP131b, SYP131b |
| Os06g0168600 | Ribonucleotide reductase, Chloroplast biogenesis (Os06t0168600-01) | V3, v3, RNRL1, rnrl1 | V3 | v3, RNRL1, rnrl1, RNR1, RNRL, Osv3, OsRNRL1, OsRNR1, OsRNRL |
| Os06g0169001 | GOS9 protein. (Os06t0169001-01) |  | JRL15 | OsJRL15 |
| Os06g0335500 | AUX/IAA protein family protein. (Os06t0335500-01);Similar to Auxin-responsive protein IAA21. (Os06t0335500-02) |  | IAA21 | OsIAA21 |
| Os06g0336200 | Delta-tonoplast intrinsic protein. (Os06t0336200-01);Similar to cDNA clone:001-035-G09, full insert sequence. (Os06t0336200-02) |  | TIP2;2 | OsTIP2;2, TIP2-2 |
| Os06g0343100 | Similar to ATP-dependent helicase DHX8 (RNA helicase HRH1) (DEAH-box protein 8). (Os06t0343100-01);Similar to predicted protein. (Os06t0343100-02) |  | | |
| Os07g0158300 | Similar to RNA binding protein. (Os07t0158300-01) |  | CRP3 | OsCRP3, cpRNP3 |
| Os07g0162400 | Alpha/beta hydrolase fold-3 domain containing protein. (Os07t0162400-01) |  | OsCDAP1, GID1L2 | OsCDAP1, GID1L2 |
| Os07g0182000 | Basic leucine zipper transcriptional activator, Grain filling (Os07t0182000-01) | RISBZ1, OsbZIP58, OsEnS-92 | RISBZ1 | OsbZIP58, bZIP58, OsEnS-92, OsSMF1, SMF1, OsRISBZ1, RISBZ1/bZIP58 |
| Os07g0192000 | ATPase, AAA-type, core domain containing protein. (Os07t0192000-01) |  |  |  |
| Os07g0195100 | Similar to Ras-related protein ARA-3. (Os07t0195100-01);Similar to Ras-related protein ARA-3. (Os07t0195100-02) |  | OsRab8B1 | OsRab8B1 |
| Os07g0205500 | Protein of unknown function DUF239, plant domain containing protein. (Os07t0205500-01) |  |  |  |
| Os07g0164900 | Similar to ABA aldehyde oxidase. (Os07t0164900-01) |  | AAO | OsAAO, OsAAO1, AAO1 |
| Os07g0180900 | Similar to 60S ribosomal protein L4. (Os07t0180900-01) |  |  |  |
| Os07g0490500 | Xanthine/uracil/vitamin C permease family protein. (Os07t0490500-01) |  | NAT | NAT |
| Os07g0159800 | Phosphatidylinositol transfer protein, Class IV ML protein, Important roles in rice immunity, Response to M. oryzae infection (Os07t0159800-01);Similar to ML domain protein. (Os07t0159800-02) | OsMl-1, OsML-1 | ML-1 | OsML-1, ML1, OsML1 |
| Os07g0162700 | Alpha/beta hydrolase fold-3 domain containing protein. (Os07t0162700-01) |  | OsCDAP2 | OsCDAP2 |
| Os07g0182100 | Similar to Tryptophan synthase alpha chain. (Os07t0182100-01) |  | TSA | OsTSA, pOsTSA, TSbeta, TSA1, OsTSA1 |
| Os07g0184800 | AT-hook DNA-binding protein, AT-hook transcription factor, Transcriptional regulation of Prx family genes, Modulation of peroxidase-mediated ROS immune response (Os07t0184800-01) | OsATH1 | ATH1 | OsATH1 |
| Os07g0186000 | H-type thioredoxin, Regulation of the apoplastic reactive oxygen species, Stress response (Os07t0186000-02) | TRXH, trxh, Trx-H, OsTRXh1, OsTrxh1, Os1, OsTrx23, RPP13-1 | TRXH | trxh, Trx-H, OsTRXh1, TRXh1, OsTrxh1, Os1, OsTrx23, Trx23, RPP13-1 |
| Os07g0193000 | HIPL1 protein precursor. (Os07t0193000-01) |  |  |  |
| Os07g0195300 | Hypothetical conserved gene. (Os07t0195300-00) |  | OsFbox347, Os_F0072, OsFBX220, FBX220 | OsFbox347, Os_F0072, OsFBX220, FBX220 |
| Os07g0196300 | Cyclin-like F-box domain containing protein. (Os07t0196300-01) |  | OsFbox348, Os_F0035, OsFBX221, FBX221 | OsFbox348, Os_F0035, OsFBX221, FBX221 |
| Os07g0201300 | UDP-glucuronosyl/UDP-glucosyltransferase domain containing protein. (Os07t0201300-00) |  |  |  |
| Os07g0204100 | Conserved hypothetical protein. (Os07t0204100-01) |  |  |  |
| Os07g0204900 | Similar to Zeta-carotene desaturase (Fragment). (Os07t0204900-01);Similar to Zeta-carotene desaturase. (Os07t0204900-02);Similar to Zeta-carotene desaturase. (Os07t0204900-03) |  | ZDS | OsZDS |
| Os07g0206700 | Cycloartenol-C-24-methyltransferase 1 (EC 2.1.1.41) (24-sterol C- methyltransferase 1) (Sterol C-methyltransferase 1). (Os07t0206700-01);Similar to Cycloartenol-C-24-methyltransferase 1. (Os07t0206700-02) |  | SMT1-1 | Ossmt1-1, OsSMT1-1 |
| Os07g0207100 | Protein kinase, core domain containing protein. (Os07t0207100-01) |  |  |  |
| Os07g0230500 | Similar to endo/excinuclease amino terminal domain-containing protein. (Os07t0230500-00) |  |  |  |
| Os07g0230600 | Hypothetical conserved gene. (Os07t0230600-00) |  |  |  |
| Os07g0232800 | Zinc transporter, Zn uptake and distribution (Os07t0232800-01);Similar to Zinc transporter protein ZIP1. (Os07t0232800-02) | OsZIP8 | ZIP8 | OsZIP8, OsZIP1 |
| Os07g0235200 | Protein of unknown function DUF296 domain containing protein. (Os07t0235200-01);AT-hook motif nuclear-localized (AHL) gene family protein 13, Response to drought and salt stress (Os07t0235200-02) | OsAHL13 | AHL13 | OsAHL13 |
| Os07g0241500 | UDP-glucuronosyl/UDP-glucosyltransferase family protein. (Os07t0241500-01) |  | UGT710C2 | OsUGT710C2 |
| Os07g0431160 | Similar to Thionin-like peptide. (Os07t0431160-00) |  |  |  |
| Os07g0443500 | Molecular chaperone, heat shock protein, Hsp40, DnaJ domain containing protein. (Os07t0443500-00) |  | MYB family transcription factor | |
| Os08g0148600 | Similar to Endoribonuclease Dicer homolog 3a. (Os08t0148600-01);Similar to Endoribonuclease Dicer homolog 3a. (Os08t0148600-02) |  | | |
| Os08g0137800 | Cupredoxin domain containing protein. (Os08t0137800-01) |  | UCL24 | OsUCL24 |
| Os08g0138200 | Similar to blue copper protein. (Os08t0138200-00) |  | UCL27 | OsUCL27 |
| Os08g0230800 | S-Domain receptor like kinase-56 (Os08t0230800-01) | SDRLK-56 | SDRLK56 | OsLecRK paralog, SDRLK-56, OsSDRLK-56, OsSDRLK56 |
| Os08g0404900 | Hypothetical conserved gene. (Os08t0404900-00) |  |  |  |
| Os08g0234000 | Similar to 60S ribosomal protein L7-2. (Os08t0234000-01);Similar to 60S ribosomal protein L7-2. (Os08t0234000-02) |  | | |
| Os08g0269700 | Conserved hypothetical protein. (Os08t0269700-01) |  |  |  |
| Os08g0407600 | Protein of unknown function DUF581 family protein. (Os08t0407600-01) |  | FLZ24 | OsFLZ24 |
| Os08g0137900 | Similar to Chemocyanin precursor (Basic blue protein) (Plantacyanin). (Os08t0137900-01) |  | UCL25 | OsUCL25 |
| Os08g0139100 | Multiple organellar RNA editing factor, Biogenesis of chloroplast ribosomes, Chloroplast development, Seedling survival (Os08t0139100-01) | OsMORF9 | MORF9 | OsMORF9 |
| Os08g0139200 | Similar to F-171-b1_1 (Fragment). (Os08t0139200-01) |  |  |  |
| Os08g0140300 | Aromatic L-amino acid decarboxylase (AADC), Senescence-induced serotonin biosynthesis (Os08t0140300-01) | TDC1, OsTDC, TDC2 | TDC1 | OsTDC, TDC, TDC2, OsTDC1, OsTDC4 |
| Os08g0141400 | Similar to mRNA, clone: RTFL01-03-I20. (Os08t0141400-01) |  | NDB3 | OsNDB3 |
| Os08g0143700 | Protein of unknown function DUF676, hydrolase-like domain containing protein. (Os08t0143700-01) |  | |  |
| Os08g0236400 | S-Domain receptor like protein-5 (Os08t0236400-00) | SDRLP-5 | RLCK248 | OsRLCK248, SDRLP-5, OsSDRLP-5, SDRLP5, OsSDRLP5 |
| Os08g0237000 | Xyloglucan endotransglucosylases/hydrolase, Cell wall modification processes during rice growth and development (Os08t0237000-01) | OsXTH8 | XTH17 | OsXTH17, OsXTH8, XTH8 |
| Os08g0267300 | Peptidase A1 domain containing protein. (Os08t0267300-00) |  |  |  |
| Os08g0276400 | Serine/threonine protein kinase domain containing protein. (Os08t0276400-01) |  |  |  |
| Os08g0360100 | RNA-binding, CRM domain domain containing protein. (Os08t0360100-01) |  | CRS1 | OsCRS1 |
| Os08g0395700 | Conserved hypothetical protein. (Os08t0395700-01) |  |  |  |
| Os08g0395800 | Protein of unknown function DUF247, plant family protein. (Os08t0395800-01) |  |  |  |
| Os08g0398400 | Hypersensitive induced reaction protein, Bacterial pathogen resistance (Os08t0398400-01) | OsHIR1, OsHSI1 | HIR1 | OsHIR1, OsHSI1 |
| Os08g0403300 | Heavy metal transport/detoxification protein domain containing protein. (Os08t0403300-00) |  | HIPP46 | OsHMP39, HMP39, OsHIPP46, OsaHIP46, HIP46 |
| Os08g0404500 | C1-like domain containing protein. (Os08t0404500-01);C1-like domain containing protein. (Os08t0404500-02) |  |  |  |
| Os08g0408500 | Drought-inducible transcriptional activator, ERF transcription factor, Regulation of calmodulin-like protein, Drought tolerance, Salt tolerance (Os08t0408500-01) | OsERF48, OsDRAP1 | ERF48 | OsERF#048, OsERF048, OsERF48, AP2/EREBP#170, AP2/EREBP170, OsDRAP1, DRAP1, OsERF48/OsDRAP1 |
| Os08g0410500 | Similar to carbohydrate transporter/ sugar porter/ transporter. (Os08t0410500-01) |  | PTR | PTR |
| Os09g0509500 | Diacylglycerol acyltransferase domain containing protein. (Os09t0509500-00) |  |  |  |
| Os09g0505300 | Similar to Br FatA1. (Os09t0505300-01) |  | FATA | OsFatA, FatA |
| Os09g0507800 | 60S ribosomal protein L7a. (Os09t0507800-01) |  | surf-3 | surf-3 |
| Os09g0509700 | Zinc finger, B-box domain containing protein. (Os09t0509700-01) |  | BBX28 | OsBBX28, OsCCT32, OsQ, CCT32 |
| Os09g0463300 | Domain of unknown function DUF547 domain containing protein. (Os09t0463300-01);Hypothetical conserved gene. (Os09t0463300-02) |  | | |
| Os09g0464000 | Similar to Carbonate dehydratase-like protein. (Os09t0464000-01);Similar to Carbonate dehydratase-like protein. (Os09t0464000-02) |  | BETACA2 | OsbetaCA2, betaCA2 |
| Os09g0464033 | Hypothetical gene. (Os09t0464033-01) |  |  |  |
| Os09g0467300 | Similar to PUP1. (Os09t0467300-01);Protein of unknown function DUF6, transmembrane domain containing protein. (Os09t0467300-02) |  | PUP2 | OsPUP2 |
| Os09g0467400 | Similar to ATPUP3. (Os09t0467400-02);Protein of unknown function DUF6, transmembrane domain containing protein. (Os09t0467400-03) |  | PUP3 | OsPUP3 |
| Os09g0468000 | Multi antimicrobial extrusion protein MatE family protein. (Os09t0468000-01) |  | MATE35 | OsMATE35 |
| Os09g0468300 | Hypothetical conserved gene. (Os09t0468300-00) |  | ATL101 | OsATL101, OsRING304, RING304 |
| Os09g0468900 | Conserved hypothetical protein. (Os09t0468900-01) |  |  |  |
| Os09g0489500 | bZIP transcription factor, Tapetum development (Os09t0489500-01);Similar to DNA binding protein. (Os09t0489500-02) | OsTGA10 | BZIP74 | OsbZIP74, OsTGA10, TGA10 |
| Os09g0490400 | Beta-glucosidase 29. (Os09t0490400-00) |  | BGLU29 | Os9bglu29, OsBGlu29, OsBGLU29 |
| Os09g0491740 | Auxin efflux carrier domain containing protein. (Os09t0491740-01) |  | PILS1 | OsPILS1 |
| Os09g0491780 | Hypothetical protein. (Os09t0491780-00) |  |  |  |
| Os09g0491852 | NAD(P)-binding domain containing protein. (Os09t0491852-01) |  | CCR1, OsCCR1, OsCCR14, CCR14 | CCR1, OsCCR1, OsCCR14, CCR14 |
| Os09g0493400 | Domain of unknown function DUF547 domain containing protein. (Os09t0493400-01) |  |  |  |
| Os09g0508000 | Hypothetical protein. (Os09t0508000-01) |  |  |  |
| Os09g0511200 | Conserved hypothetical protein. (Os09t0511200-00) |  |  |  |
| Os09g0511600 | Transglucosidase (Os09t0511600-01);Non-protein coding transcript. (Os09t0511600-02);Glycoside hydrolase, family 1 protein. (Os09t0511600-03);Glycoside hydrolase, family 1 protein. (Os09t0511600-04) | Os9BGlu31 | BGLU31 | Os9bglu31, Os9BGlu31 |
| Os09g0511700 | Similar to Prunasin hydrolase isoform PH C precursor (EC 3.2.1.118). (Os09t0511700-01) |  | BGLU32 | Os9bglu32, Os9Bglu32 |
| Os09g0512800 | Similar to snRK1-interacting protein 1. (Os09t0512800-00) |  | OsSTA231 | OsSTA231 |
| Os09g0513100 | Similar to Phospholipase A1. (Os09t0513100-01);Similar to phospholipase A1. (Os09t0513100-02) |  | LCAT3 | OsLCAT3 |
| Os09g0513200 | Similar to Solute carrier family 35, member F1. (Os09t0513200-00) |  |  |  |
| Os10g0206800 | Multidrug and toxic compound extrusion (MATE) protein, Al-induced secretion of citrate (Os10t0206800-01);Similar to predicted protein. (Os10t0206800-02) | OsFRDL2 | OsFRDL2 | OsFRDL2 |
| Os10g0333700 | Plant disease resistance response protein domain containing protein. (Os10t0333700-00) |  | DIR30 | OsDIR30, OsjDIR30 |
| Os10g0330000 | Conserved hypothetical protein. (Os10t0330000-01) |  |  |  |
| Os10g0204400 | Similar to Phosphoenolpyruvate carboxykinase (Fragment). (Os10t0204400-01) |  | PEPCK, OsPEPCK | PEPCK, OsPEPCK |
| Os10g0329400 | Conserved hypothetical protein. (Os10t0329400-00) |  |  |  |
| Os10g0335000 | Dirigent protein, Lignin biosynthesis (Os10t0335000-01) |  | DIR32 | OsDIR32, OsjDIR32 |
| Os11g0533100 | Similar to CarD-like transcriptional regulator family protein, expressed. (Os11t0533100-01);ATP-dependent DNA helicase (Os11t0533100-02) |  | | |
| Os11g0126900 | NAC-domain protein, Drought tolerance (Os11t0126900-01) | NAC122, OsNAC10, ONAC122 | NAC122 | OsNAC10, NAC10, ONAC122, NAC122, OsSNAC10, SNAC10 |
| Os11g0127600 | NAC transcription factor, Drought and salt tolerance (Os11t0127600-01) | ONAC045 | NAC45 | ONAC045, ONAC45, DLN243, OsDLN243, OsNAM, NAM |
| Os11g0531700 | NUDIX hydrolase domain containing protein. (Os11t0531700-01);Similar to nudix hydrolase 13. (Os11t0531700-02) |  | NUDX13 | OsNUDX13 |
| Os11g0127900 | Similar to 40S ribosomal protein S16. (Os11t0127900-01) |  | OsRP1 | OsRP1 |
| Os11g0130200 | Protein of unknown function DUF309 family protein. (Os11t0130200-01) |  |  |  |
| Os11g0526200 | Similar to predicted protein. (Os11t0526200-01) |  |  |  |
| Os11g0116900 | WRKY transcription factor 46, Disease resistance to Magnaporthe oryzae (Os11t0116900-01) | OsWRKY46 | WRKY46 | OsWRKY46, WRKY91, OsWRKY91 |
| Os11g0117400 | WRKY transcription factor, Ultraviolet B tolerance, Disease resistance (Os11t0117400-01) | OsWRKY89, OsWRKY104 | WRKY104 | OsWRKY104, CRPG32, OsCRPG32 |
| Os11g0117600 | WRKY transcription factor 50, Transcription repressor, Mediation of ABA-dependent seed germination and seedling growth, Salt stress tolerance (Os11t0117600-01) | OsWRKY50 | WRKY50 | OsWRKY50 |
| Os11g0117900 | Similar to Hydrolase, alpha/beta fold family protein, expressed. (Os11t0117900-01) |  |  |  |
| Os11g0119100 | Similar to Catalytic/ hydrolase. (Os11t0119100-01) |  |  |  |
| Os11g0127800 | Similar to FHA domain containing protein, expressed. (Os11t0127800-01);Similar to FHA domain containing protein, expressed. (Os11t0127800-02) |  | FHA domain containing protein | |
| Os11g0283500 | TGF-beta receptor, type I/II extracellular region family protein. (Os11t0283500-01) |  | PTR | PTR |
| Os11g0528500 | Similar to Rubredoxin 1 (Rd-1). (Os11t0528500-01) |  |  |  |
| Os11g0528700 | Indole-3-acetic acid (IAA)-amido synthetase, Plant architecture establishment, Drought tolerance (Os11t0528700-01);Indole-3-acetic acid (IAA)-amido synthetase (Os11t0528700-02) | GH3-13, OsGH3-13, TLD1/OsGH3.13, TLD1, OsGH3.13, TLD2 | TLD1 | OsGH3-13, GH3-13, TLD1/OsGH3.13, TLD1, OsGH3.13, TLD2 |
| Os12g0564100 | Similar to Myb-like DNA-binding domain containing protein, expressed. (Os12t0564100-01);R2R3-MYB transcription factor 97 (Os12t0564100-02) | Os2R_MYB97 | 2R_MYB97 | R2R3-MYB, Os2R_MYB97 |
| Os12g0211400 | A/G-specific adenine glycosylase MutY, bacterial form domain containing protein. (Os12t0211400-00) |  | |  |
| Os12g0211900 | Hypothetical conserved gene. (Os12t0211900-01) |  |  |  |
| Os12g0218100 | Similar to cDNA, clone: J065028F04, full insert sequence. (Os12t0218100-01) |  |  |  |
| Os12g0291100 | Similar to Petunia ribulose 1,5-bisphosphate carboxylase small subunit mRNA (clone pSSU 51), partial cds. (Fragment). (Os12t0291100-01);Similar to Ribulose bisphosphate carboxylase small chain. (Os12t0291100-02);Non-protein coding transcript. (Os12t0291100-03) |  | RBCS3 | RbcS3, OsRBCS3, OsRbcS3, rbcS |
| Os12g0292400 | Similar to Petunia ribulose 1,5-bisphosphate carboxylase small subunit mRNA (clone pSSU 51), partial cds. (Fragment). (Os12t0292400-01) |  | RBCS4 | RbcS, RbcS4, OsRBCS4, OsRbcS4 |
| Os12g0564400 | Similar to Thylakoid lumenal 21.5 kDa protein, chloroplast precursor. (Os12t0564400-01);Similar to PsbP. (Os12t0564400-02) |  | PsbP | PsbP |
| Os12g0567800 | Plant metallothionein, family 15 protein. (Os12t0567800-01) |  | MT1F | OsMT1f, OsMT1c, MT1f, MT1c, MT1Lb, OsMT1Lb |
